# Efficient strategy for introducing large and multiple changes in plasmid DNA

**DOI:** 10.1038/s41598-018-20169-8

**Published:** 2018-01-29

**Authors:** Fanli Zeng, Suhua Zhang, Zhimin Hao, Shixin Duan, Yanan Meng, Pan Li, Jingao Dong, Yibin Lin

**Affiliations:** 1 College of Life Sciences, Hebei Agricultural University, Baoding, 071001 China; 2 Institute of Biophysics, Hebei University of Technology, Tianjin, 300401 China; 3 Department of Biochemistry and Molecular Biology, University of Texas Health Science Center at Houston McGovern Medical School, 6431 Fannin Street, Houston, TX 77030 USA

## Abstract

While the QuikChange site-directed mutagenesis method and its later modifications are extremely useful and simple, they suffer from several drawbacks. Here, we propose a new method, named LFEAP mutagenesis (Ligation of Fragment Ends After PCR) for creating various mutations in plasmid by leveraging three existing concepts: inverse PCR, single primer PCR, and sticky-end assembly. The first inverse PCR on the target plasmid yielded linearized DNA fragments with mutagenic ends, and a second single primer PCR resulted in complementary single-stranded DNA fragments with the addition of overhangs at the 5′ end of each strand. The resulting single strands were then annealed to produce double-stranded DNA with free 5′ single-stranded DNA tails. These products with compatible sticky ends were efficiently assembled into a circular, mutagenized plasmid. With this strategy, multiple simultaneous changes (up to 15) and mutations in large plasmids (up to 50 kb) were achieved with high efficiency and fidelity. LFEAP mutagenesis is a versatile method that offers significant advantages for introducing large and multiple changes in plasmid DNA.

## Introduction

Polymerase chain reaction (PCR)-based site-directed mutagenesis is an invaluable technique for altering genes and hence the structure and activity of individual proteins in a systematic way, opening up opportunities for investigating the structure-function relationships of protein, enzyme specificity and selectivity, or protein engineering^1–3^.

In the past decade, a number of strategies and commercial kits have been developed for introducing mutational changes in plasmid DNA, such as base substitutions and base additions or deletions. Among them, Stratagene’s QuikChange site-directed mutagenesis kit is extremely useful and simple, and probably one of the most favored^4^. It requires a high-fidelity DNA polymerase that minimizes unwanted mutations, such as KOD hot start DNA polymerase, Pfu DNA polymerase, or Phusion^®^ high-fidelity DNA polymerase, to amplify the whole plasmid with complementary primer pairs, carrying the desired mutation in the form of mismatches to the original plasmid. The parental DNA template is eliminated by treating with *Dpn*I, which destroys the methylated template DNA^5^. The resulting nicked DNA is transformed into competent *E. coli* cells for nick repair.

Despite its widespread use, the QuikChange system has limitations. The fact that the primers are completely complementary, and hence favor self-annealing limits the PCR product yield and gives rise to false positives^6^. The complementary primer pairs favor “primer-dimer” formation by partial annealing of a primer with the second primer in the reaction, instead of primer annealing to the template with mismatches, which causes low PCR amplification efficiency, and may lead to the formation of tandem primer repeats in resulting PCR products and hence a reduction in fidelity^7,8^. The complementary primer design results in the mutated plasmid containing staggered nicks, and thus the newly synthesized DNA cannot be used as a template for subsequent amplification^4^. In addition, the originally developed QuikChange method requires the altered nucleotides to be introduced in the middle of both primers, limiting the introduction of multiple mutations^4^ as well as large changes^9^.

To circumvent these limitations, many modified versions of the QuikChange site-directed mutagenesis method have been developed^4,10–12^. These methods use partially overlapping primers to reduce the formation of primer dimers and hence improve PCR amplification efficiency. Despite high efficiency, these approaches require primers containing the desired mutations in the template annealing regions, which limits the introduction of large changes required in some functional studies. Recently, several labs reported alternatives, such as overlap extension PCR (OE-PCR)^13–16^ and homologous recombination-based methods^17–25^, for creating mutations *in vitro* or *in vivo*. While OE-PCR provides efficient methods for introducing multiple and large changes, they involve multiple rounds of PCR and DNA purification, limiting the creation of multiple mutations simultaneously. Homologous recombination-based approaches rely on *in vitro* enzymatic treatment of DNA fragments for assembly. They always suffer from lower efficiency and fidelity when introducing mutations at more than five sites since the simultaneous assembly of more than five fragments is difficult, contributing to lower efficiency^26^ and non-specific recombination events^24^. Given these limitations, we aimed to develop more flexible protocols for making specific mutations.

Inspired by the concept of restriction-free cloning^27^ and recent advances in DNA sequence assembly^28^, we developed a new system for generating large and multiple changes in plasmid DNA. This system requires two rounds of PCR and subsequent annealing to generate mutated DNA fragments with compatible “sticky hands” at their 5′ ends for “hand-in-hand” assembly. Since the system requires two rounds of PCR followed by ligation of the sticky ends of the resulting DNA fragments, we named the method LFEAP mutagenesis (Ligation of Fragment Ends After PCR). Using this method, we can create a variety of DNA modifications, such as point mutations, substitutions, deletions, insertions, and multiple-site mutations in vectors in a cost-efficient manner with high efficiency and fidelity.

## Results

### Method overview

**Figure 1:**
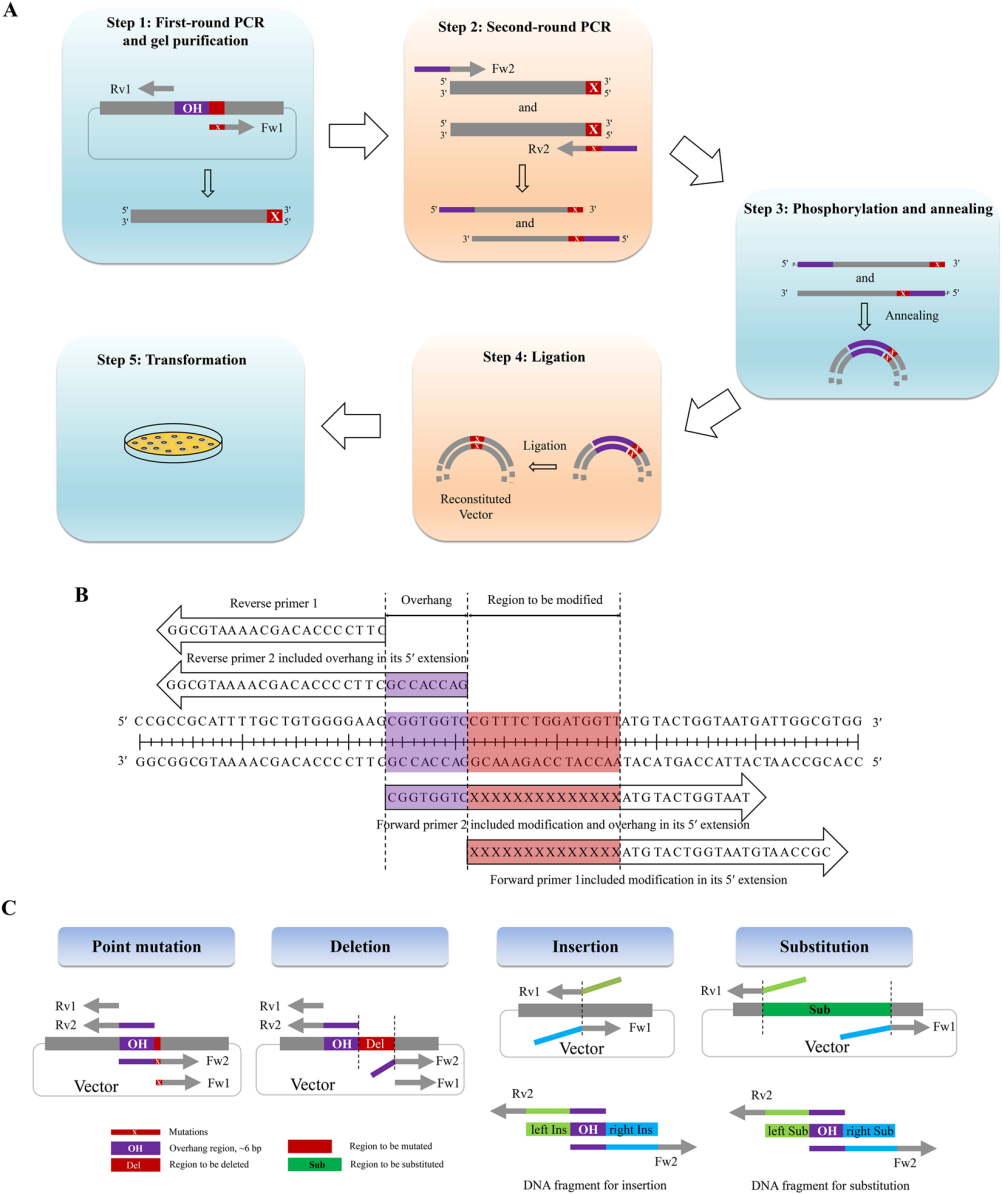
Schematic representation of the LFEAP mutagenesis procedure for basic mutations. (**A**) The mutagenesis reaction requires five steps. Step 1: first-round PCR to introduce mutation using primers Fw1 and Rv1. Step 2: second-round PCR to introduce overhang using DNA fragments generated in the first-round PCR as templates and primer Fw2 or Rv2. Step 3: phosphorylation and annealing of two single-strand DNA fragments generated in the second-round PCR. Step 4: ligation of annealed fragments generated in step 3. Step 5: transformation of ligation products into competent *E. coli* cells. (**B**) An example showing primer design, overhang region, and region to be mutated. (**C**) The mechanism of LFEAP mutagenesis for generating a point mutation, deletion, insertion, and substitution. Fw: forward primer, Rv: reverse primer, OH: overhang region, Del: region to be deleted, Ins: DNA sequence to be inserted, Sub: region to be substituted.

The mechanism of LFEAP mutagenesis for generating basic mutations, such as point mutations, substitutions, deletions, and insertions, is shown in Fig. 1. To generate basic mutations, LFEAP mutagenesis requires an “overhang” region and four primers. As shown in Fig. 1B and C, the overhang sequence can be a short sequence at the 5′ terminus of the region to be mutated (for point mutation, deletion, and insertion and substitution of short DNA sequences), or inside the region to be modified (for insertion and substitution of long DNA sequences). The primers designed for basic mutations are shown in Fig. 1B and C. Forward primer 1 (Fw1) and reverse primer 1 (Rv1) were designed to flank the overhang region. Fw1 contained mutations at its 5′ end that were incorporated into the first-round PCR products. Forward primer 2 (Fw2) and reverse primer 2 (Rv2) were designed to have additional overhang sequence at the 5′ ends that were incorporated into the second-round PCR products. All types of basic mutations proceeded similarly (Fig. 1A). (i) The first-round PCR was the exponential amplification of the target vector using Fw1 and Rv1. Fw1 contained mutations at its 5′ extension, and so the resulting PCR products contained mutations at the 5′ extension. The PCR products were gel purified to remove primers and templates. (ii) The second-round PCR used the DNA products generated in the first-round PCR as templates and Fw2 or Rv2 alone to generate single-stranded DNA fragments. The Fw2 and Rv2 contained overhangs at their 5′ extension. The resulting PCR products contained an overhang at the 5′ terminus. (iii) After treating with polynucleotide kinase (PNK) for 5′ phosphorylation, the two complementary single-stranded DNAs generated in the second-round PCR were then annealed to form double-stranded DNA with 5′ protruding ends. (iv) The double-stranded DNAs with sticky ends were joined using DNA ligase. (v) These ligated products were transformed into competent *E. coli* cells, and the presence of modifications was confirmed by DNA sequencing.

**Figure 2:**
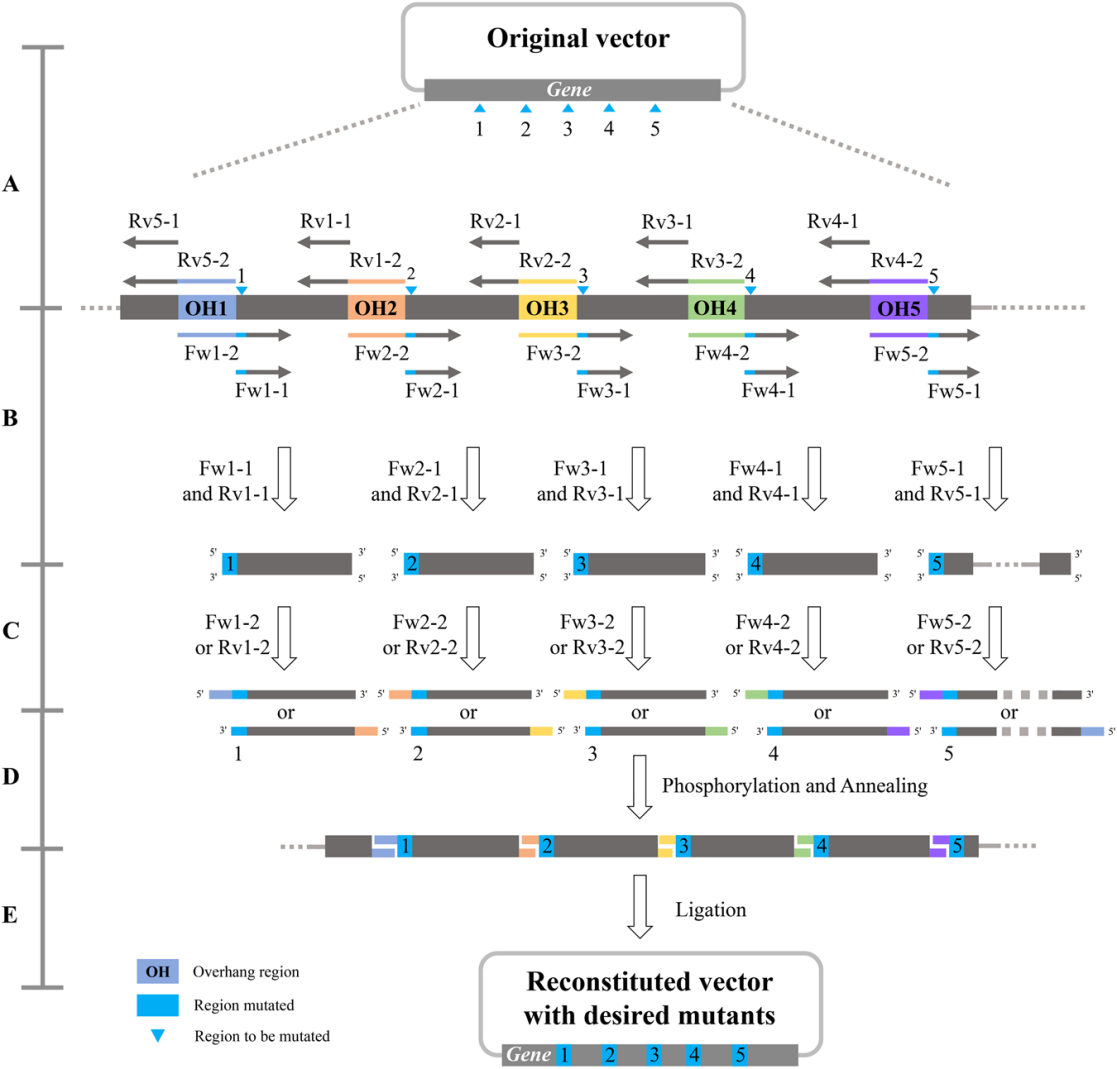
Schematic details of the generation of multiple mutations with LFEAP mutagenesis. (**A**) Primer design. For each modification, a 6–10 nucleotide region that is adjacent to the 5′ end of the mutation region is assigned as an overhang region. The mutagenesis reaction requires four steps: (**B**) first-round PCR to introduce mutations using primer pairs; (**C**) second-round PCRs to incorporate overhangs at the 5′ ends of resulting DNA products using DNA products generated in the first-round PCR as templates and single primers; (**D**) phosphorylation and annealing of two complementary single-stranded DNA fragments generated in the second-round PCR; and (**E**) ligation of the annealed multi-part DNAs with sticky ends and transformation into competent *E. coli* cells. Fw: forward primer, Rv: reverse primer, OH 1–5: overhang regions.

The procedure for introducing mutations at multiple sites is shown in Fig. 2. The multiple-site mutagenesis can be considered as a combination of many basic mutations. Each site mutation requires an overhang region and four primers as for basic mutations (Fig. 2A). The procedure for introducing multiple changes with LFEAP mutagenesis required four steps. (i) In the first-round PCR, five PCRs in parallel were performed to generate five double-stranded DNA fragments using primer pairs Fw1-1 and Rv1-1, Fw2-1 and Rv2-1, Fw3-1 and Rv3-1, Fw4-1 and Rv4-1, and Fw5-1 and Rv5-1. The resulting PCR products contained the desired mutations at their 5′ extension. (ii) In the second-round PCR, two single primer PCRs in parallel were performed to generate two complementary single-stranded DNA fragments using each fragment generated in the first-round PCR as the template and single primers of Fw1-2 or Rv1-2, Fw2-2 or Rv2-1, Fw3-2 or Rv3-2, Fw4-2 or Rv4-2, and Fw5-1 or Rv5-1. (iii) After treating with PNK, the complementary single-stranded DNA products were then annealed to form double-stranded DNAs with sticky ends. (iv) The annealed multi-part DNAs with sticky ends were sealed by DNA ligase to form a transformable plasmid.

### Optimal overhang adapter sequence

**Figure 3:**
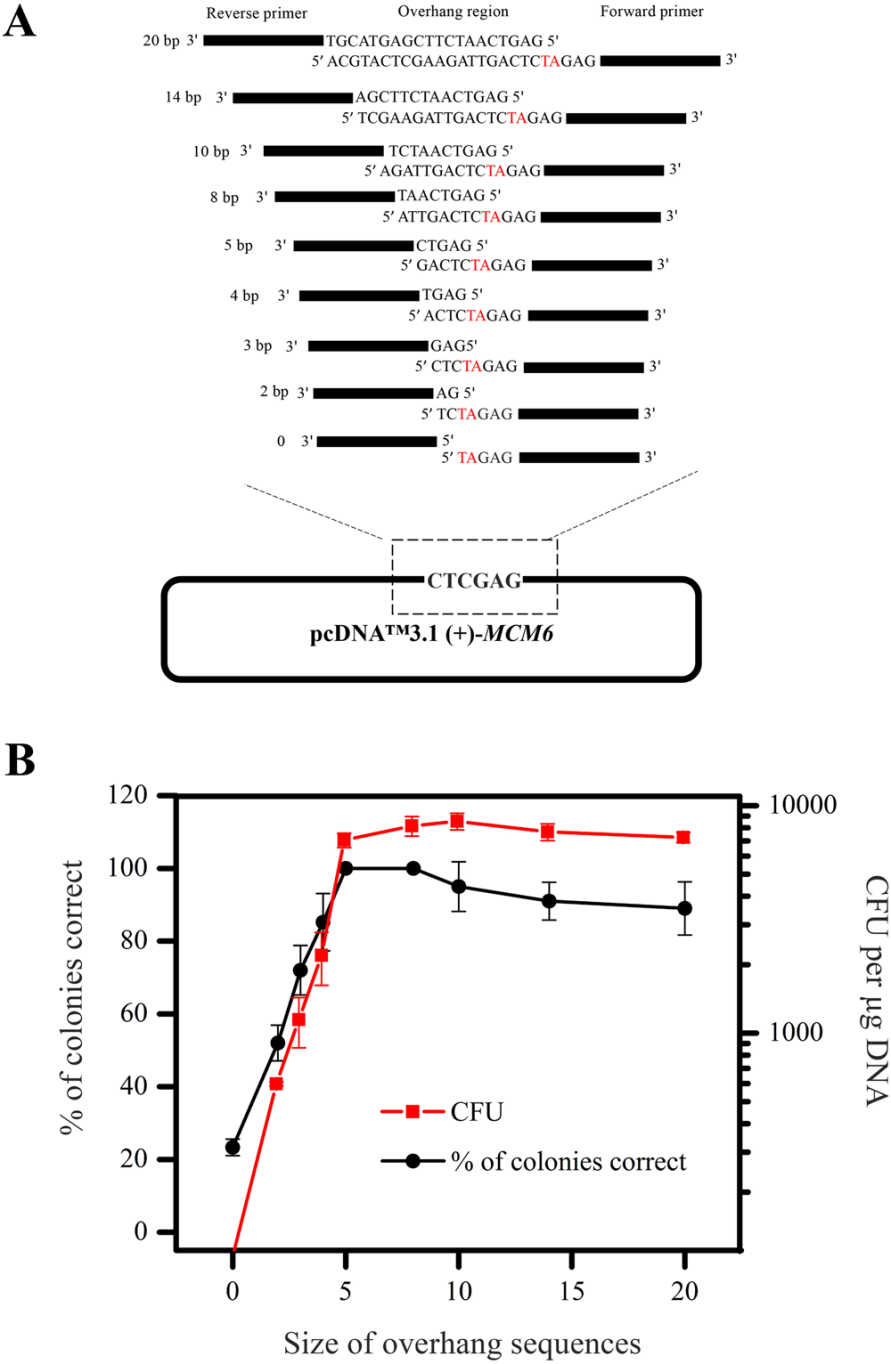
Effect of overhang size on LFEAP mutagenesis efficiency. (**A**) Schematic details show the primer design for determining optimal overhang size. The mutations are highlighted with red letters. (**B**) The overhang size is plotted against the achieved efficiencies and fidelities. Reported results are the mean ± s.d. of three independent experiments. Overhangs of 6–10 nucleotides give the maximum efficiency and fidelity, while a decrease in efficiency and fidelity is observed when longer overhangs are used.

To identify the optimal overhang sequence required for LFEAP mutagenesis, we followed the procedure as shown in Fig. 1A to add two nucleotides (TA) in the middle of the *Xho*I restriction site (CTCGAG) in pcDNA™3.1 (+)-*MCM6* plasmid, thereby disrupting the restriction site^29^ using a series of primers with 5′ overhangs ranging from 0 to 20 nucleotides (see Fig. 3A for primer design). We evaluated the performance of LFEAP mutagenesis by determining the efficiency (colony forming units (CFUs) per microgram of ligated DNA after transformation) and the fidelity (percentage of clones containing the desired mutations). The mutations were carried out by LFEAP mutagenesis, and the resulting plasmids were extracted from the transformed *E. coli*. An overhang sequence of 0 to 3 nucleotides in length in the resulting PCR products was insufficient for efficient mutagenesis (Fig. 3B). Overhangs of four or more nucleotides resulted in the efficiency and fidelity of mutagenesis reactions increasing sharply up to 10 nucleotides, with a maximum efficiency of approximately 8,000 CFUs and fidelity of 100%. Interestingly, no further improvement in efficiency and fidelity was observed when continually increasing the length of the overhang sequence. On the contrary, the efficiency and fidelity suffered a slight decrease when longer overhang sequences were used (Fig. 3B). We conclude that an overhang of 6–10 nucleotides is optimal for LFEAP mutagenesis.

### Basic mutations

To investigate the capability of LFEAP mutagenesis for basic mutations, we provided examples from our work using genes (*yaaU*, *ileS*, *talB* and *apaG* cloned from the *E. coli* genome and *GAST*, *MCM6*, *PRRT2*, and *SLC18A2* cloned from a human cDNA library) cloned into pNGFP-BC or pCGFP-EU vector, and ran LFEAP mutagenesis to produce mutations at desired sites (Fig. 1C; see Supplementary Methods for detailed experimental procedures). The primers used for creating mutations were designed following the rules shown in Fig. 1B and are listed in Supplementary Table S1. The DNA products resulting from LFEAP mutagenesis were evaluated by 1% agarose gel electrophoresis, and the presence of desired mutations was verified via DNA sequencing (see Supplementary Figure S1 for point mutations, Supplementary Figure S2 for substitutions, Supplementary Figure S3 for deletions, and Supplementary Figure S4 for insertions).

1. Point mutations. As examples, we performed seven point mutations within plasmid coding sequences: *yaaU* (R205A), *ileS* (K581A), *talB* (K193C), *apaG* (R26A), *GAST* (K75A), *MCM6* (Q641A), and *SLC18A2* (K354A). Under our test conditions, 8,000–9,000 CFUs and an average fidelity of 98.5% were achieved with LFEAP mutagenesis (Table 1). By contrast, commercial QuikChange mutagenesis yielded fewer CFUs (7,000–8,000) and a lower average fidelity of 86.2% (Supplementary Table S5).

**Table 1:** The efficiency and fidelity of creating point mutations with LFEAP mutagenesis.

Gene	Gene ID	Size (bp)	Vector	Mutation	CFUs/µg DNA^a^	Positive (%)^b^
*yaaU*	944766	1,332	pNGFP-BC	R205A	9672 ± 593	96.7 ± 5.8
*ileS*	944761	2,817	pCGFP-BC	K581A	8254 ± 782	90.0 ± 10.0
*talB*	944748	954	pNGFP-BC	K193C	8833 ± 714	93.3 ± 11.5
*apaG*	944772	378	pCGFP-BC	R26A	9755 ± 868	96.7 ± 5.8
*GAST*	2520	306	pNGFP-EU	K75A	8636 ± 874	90.0 ± 10.0
*MCM6*	4175	2,466	pNGFP-EU	Q641A	8571 ± 883	96.7 ± 5.8
*SLC18A2*	6571	1,545	pCGFP-EU	K354A	8931 ± 993	100.0 ± 0.0

^a^Reported results are the mean ± s.d. of three independent experiments.

^b^For each independent experiment, ten of colonies were checked by DNA sequencing.
2. Substitutions. As examples, we performed i) substitution of six nucleotides in *yaaU* and nine nucleotides in *GAST*, and ii) substitution of 30 nucleotides in *yaaU* and 36 nucleotides in *GAST*. Accordingly, thousands of colonies and nearly 100% fidelity were obtained (Table 2).

**Table 2:** The efficiency and fidelity of creating substitutions with LFEAP mutagenesis.

Gene	Gene ID	Size (bp)	Vector	Mutation	CFU/µg DNA^a^	Positive (%)^b^
*yaaU*	944766	1,332	pNGFP-BC	DE224AA	7375 ± 18	100 ± 0
*GAST*	2520	306	pNGFP-EU	SQQ27AAA	7296 ± 16	96.7 ± 5.8
*yaaU*	944766	1,332	pNGFP-BC	RKGRVKECEE202AAAAAAAAAA	8178 ± 21	100 ± 0
*GAST*	2520	306	pNGFP-EU	EQQGPASHHRRQ48AAAAAAAAAAAA	7827 ± 15	100 ± 0

^a^Reported results are the mean ± s.d. of three independent experiments.

^b^For each independent experiment, ten of colonies were checked by DNA sequencing.
3. Deletions. As examples, we performed (i) deletion of single nucleotides, i.e., *yaaU* 909 A, *ileS* 2096 T, *talB* 552 C, *apaG* 253 G, *GAST* 183 A, *MCM6* 1745T, *PPRT2* 741 C, and *SLC18A2* 1415 G, ii) deletion of 12 nucleotides in selected genes resulting in yaaU (Del F28–G31), ileS (Del R202–R205), talB (Del Q28–D31), apaG (Del G63–G66), GAST (Del H55–R58), MCM6 (Del D202–K205), PPRT2 (Del D43–E45), and SLC18A2 (Del D73–Q76) mutants, and iii) deletion of longer nucleotide sequences, i.e., 1,272 nucleotides from *yaaU*, 2,748 nucleotides from *ileS*, 885 nucleotides from *talB*, 309 nucleotides from *apaG*, 238 nucleotides from *GAST*, 2,397 nucleotides from *MCM6*, 954 nucleotides from *PPRT2*, and 1,476 nucleotides from *SLC18A2*. We obtained high efficiency and fidelity as verified by DNA sequencing (Table 3).

**Table 3:** The efficiency and fidelity of creating deletions with LFEAP mutagenesis.

Gene	Gene ID	Size (bp)	Vector	Mutations	CFU/µg DNA^a^	Positive (%)^b^
*yaaU*	944766	1,332	pNGFP-BC	Del 909 A	8089 ± 824	100 ± 0
*ileS*	944761	2,817	pCGFP-BC	Del 2096 T	7132 ± 717	100 ± 0
*talB*	944748	954	pNGFP-BC	Del 552 C	8259 ± 725	96.7 ± 5.8
*apaG*	944772	378	pCGFP-BC	Del 253 G	7433 ± 813	90 ± 10
*GAST*	2520	306	pNGFP-EU	Del 183 A	8319 ± 712	100 ± 0
*MCM6*	4175	2,466	pCGFP-EU	Del 1745T	6559 ± 749	96.7 ± 5.8
*PPRT2*	112476	1,023	pNGFP-EU	Del 741 C	7539 ± 721	100 ± 0
*SLC18A2*	6571	1,545	pCGFP-EU	Del 1415 G	7975 ± 816	100 ± 0
*yaaU*	944766	1,332	pNGFP-BC	Del F28-G31	6938 ± 717	90 ± 10
*ileS*	944761	2,817	pCGFP-BC	Del R202-R205	6712 ± 711	90 ± 10
*talB*	944748	954	pNGFP-BC	Del Q28-D31	9976 ± 810	96.7 ± 5.8
*apaG*	944772	378	pCGFP-BC	Del Q63-G66	9655 ± 819	100 ± 0
*GAST*	2520	306	pNGFP-EU	Del H55-R58	8575 ± 821	96.7 ± 5.8
*MCM6*	4175	2,466	pCGFP-EU	Del D202-K205	5956 ± 712	100 ± 0
*PPRT2*	112476	1,023	pNGFP-EU	Del D43-E45	7742 ± 611	100 ± 0
*SLC18A2*	6571	1,545	pCGFP-EU	Del D73-Q76	6938 ± 713	90 ± 10
*yaaU*	944766	1,332	pNGFP-BC	Del K11-N434	8675 ± 14	96.7 ± 5.8
*ileS*	944761	2,817	pCGFP-BC	Del G14-A929	7426 ± 622	90 ± 10
*talB*	944748	954	pNGFP-BC	Del V14-K308	7494 ± 819	100 ± 0
*apaG*	944772	378	pCGFP-BC	Del V14-F116	6335 ± 723	100 ± 0
*GAST*	2520	306	pNGFP-EU	Del G14-L92	9162 ± 810	96.7 ± 5.8
*MCM6*	4175	2,466	pCGFP-EU	Del Q14-V812	8494 ± 717	100 ± 0
*PPRT2*	112476	1,023	pNGFP-EU	Del V14-S331	7796 ± 725	90 ± 10
*SLC18A2*	6571	1,545	pCGFP-EU	Del E14-I505	8176 ± 821	97.6 ± 5.8

^a^Reported results are the mean ± s.d. of three independent experiments.

^b^For each independent experiment, ten of colonies were checked by DNA sequencing.
4. Insertions. As examples, we performed i) insertion of a single nucleotide into target genes yielding *yaaU* (909 A), *ileS* (2096 T), *talB* (552 C), *apaG* (253 G), *GAST* (183 A), *MCM6* (1745 T), *PPRT2* (741 C), and *SLC18A2* (1415 G) mutants, ii) insertion of 12 nucleotides into target genes, producing *yaaU* (Ins F28-AAAA), *ileS* (Ins E201-AAAA), *talB* (Ins Q28-AAAA), *apaG* (Ins Q63-AAAA), *GAST* (Ins H55-AAAA), *MCM6* (Ins D202-AAAA), *PPRT2* (Ins D43-AAAA), and *SLC18A2* (Ins D73-AAAA) mutants, and iii) insertion of 60 nucleotides into *yaaU* and *SLC18A2* yielding mutants of *yaaU* (Ins F28-VEESPKVPGEGPGHSEAETG) and SLC18A2 (Ins D73-VEESPKVPGEGPGHSEAETG). Large colony numbers and high fidelity were achieved (Table 4).

**Table 4:** The efficiency and fidelity of creating insertions with LFEAP mutagenesis.

Gene	Gene ID	Size (bp)	Vector	Mutation	CFU/µg DNA^a^	Positive (%)^b^
*yaaU*	944766	1,332	pNGFP-BC	Ins 909 A	8671 ± 56	100 ± 0
*ileS*	944761	2,817	pCGFP-BC	Ins 2096 T	7453 ± 12	100 ± 0
*talB*	944748	954	pNGFP-BC	Ins 552 C	8246 ± 22	96.7 ± 5.7
*apaG*	944772	378	pCGFP-BC	Ins 253 G	7983 ± 21	100 ± 0
*GAST*	2520	306	pNGFP-EU	Ins 183 A	7864 ± 33	100 ± 0
*MCM6*	4175	2,466	pCGFP-EU	Ins 1745T	7519 ± 19	100 ± 0
*PPRT2*	112476	1,023	pNGFP-EU	Ins 741 C	7884 ± 32	90 ± 10
*SLC18A2*	6571	1,545	pCGFP-EU	Ins 1415 G	8696 ± 12	100 ± 0
*yaaU*	944766	1,332	pNGFP-BC	Ins F28-AAAA	9515 ± 24	100 ± 0
*ileS*	944761	2,817	pCGFP-BC	Ins E201-AAAA	8473 ± 16	100 ± 0
*talB*	944748	954	pNGFP-BC	Ins Q28-AAAA	8297 ± 25	96.7 ± 5.7
*apaG*	944772	378	pCGFP-BC	Ins Q63-AAAA	8157 ± 33	96.7 ± 5.7
*GAST*	2520	306	pNGFP-EU	Ins H55-AAAA	7819 ± 18	100 ± 0
*MCM6*	4175	2,466	pCGFP-EU	Ins D202-AAAA	6468 ± 21	100 ± 0
*PPRT2*	112476	1,023	pNGFP-EU	Ins D43-AAAA	8411 ± 19	96.7 ± 5.7
*SLC18A2*	6571	1,545	pCGFP-EU	Ins D73-AAAA	7612 ± 26	100 ± 0
*yaaU*	944766	1,332	pNGFP-BC	Ins F28-VEESPKVPGEGPGHSEAETG	8536 ± 32	90 ± 10
*SLC18A2*	6571	1,545	pCGFP-EU	Ins D73- VEESPKVPGEGPGHSEAETG	9445 ± 27	96.7 ± 5.7

^a^Reported results are the mean ± s.d. of three independent experiments.

^b^For each independent experiment, ten of colonies were checked by DNA sequencing.

### Multiple-site mutations

The experiments described above demonstrated that LFEAP mutagenesis is an efficient and precise method for introducing single as well as large changes in plasmids. To test the feasibility of LFEAP mutagenesis for simultaneous introduction of multiple mutations in plasmid, we performed experiments to generate 3 (E52A, R309A, and Q668A), six (E52A, D160A, D253A, D362A, E461A, and D564A), 10 (E52A, D160A, R207A, R309A, D362A, R416A, D511A, D564A, Q668A, and D784A), and 15 (E52A, E103A, D160A, R207A, D253A, R309A, D362A, R416A, E461A, D511A, D564A, R619A, Q668A, E719A, and D784A) point mutations in the *MCM6* gene in the pNGFP-EU-*MCM6* plasmid (see Fig. 2 for a schematic detailing the procedure).

**Figure 4:**
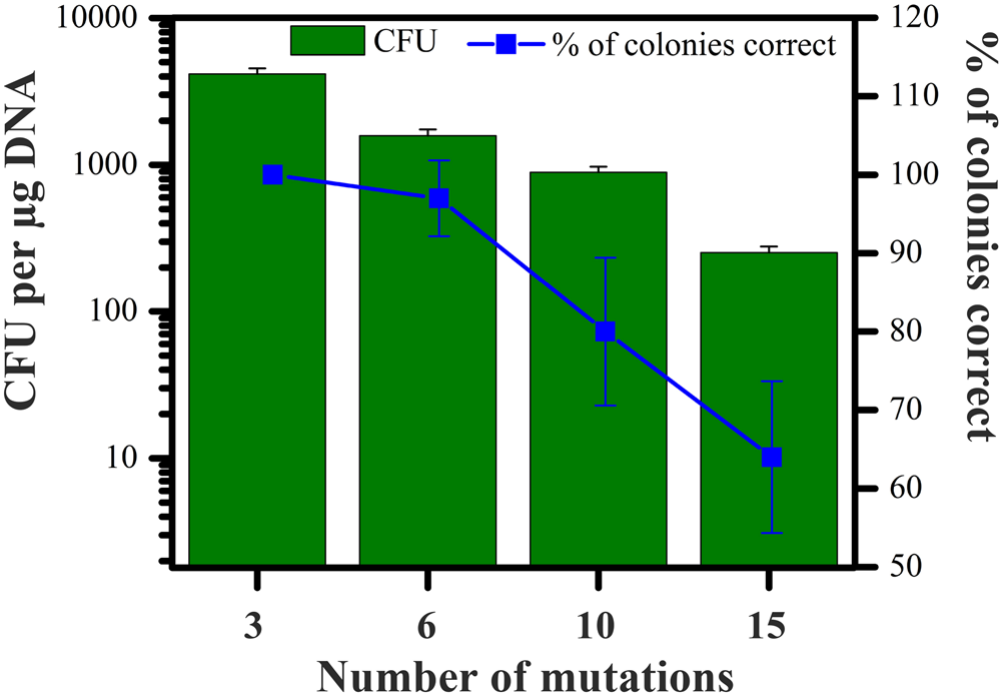
Mutations at multiple sites with LFEAP mutagenesis. The number of mutations is plotted against the achieved efficiencies and fidelities. Results are the mean ± s.d. of three independent experiments.

Multiple-site mutagenesis was performed by following the procedure as shown in Fig. 2. Accordingly, all mutations of interest were introduced into the 5′ ends of fragments in the first-round PCR step. After gel purification, these fragments were used as templates for second-round PCR to add overhang adapter sequences onto the 5′ ends, followed by annealing and ligating to form plasmids with the desired mutations. The presence of mutations was verified by DNA sequencing. Figure 4 shows the efficiency and fidelity of the generation of multiple-site modifications with LFEAP mutagenesis. As we expected, the efficiency of mutagenesis decreased with increasing number of mutations. The CFU per of DNA dipped to around 250 when simultaneously creating 15 mutations (Fig. 4). By contrast, the fidelity dropped slightly but remained above 60% even for 15 mutations. Overall, the method performs well in multiple-site mutagenesis.

### Mutations in large plasmids

**Figure 5:**
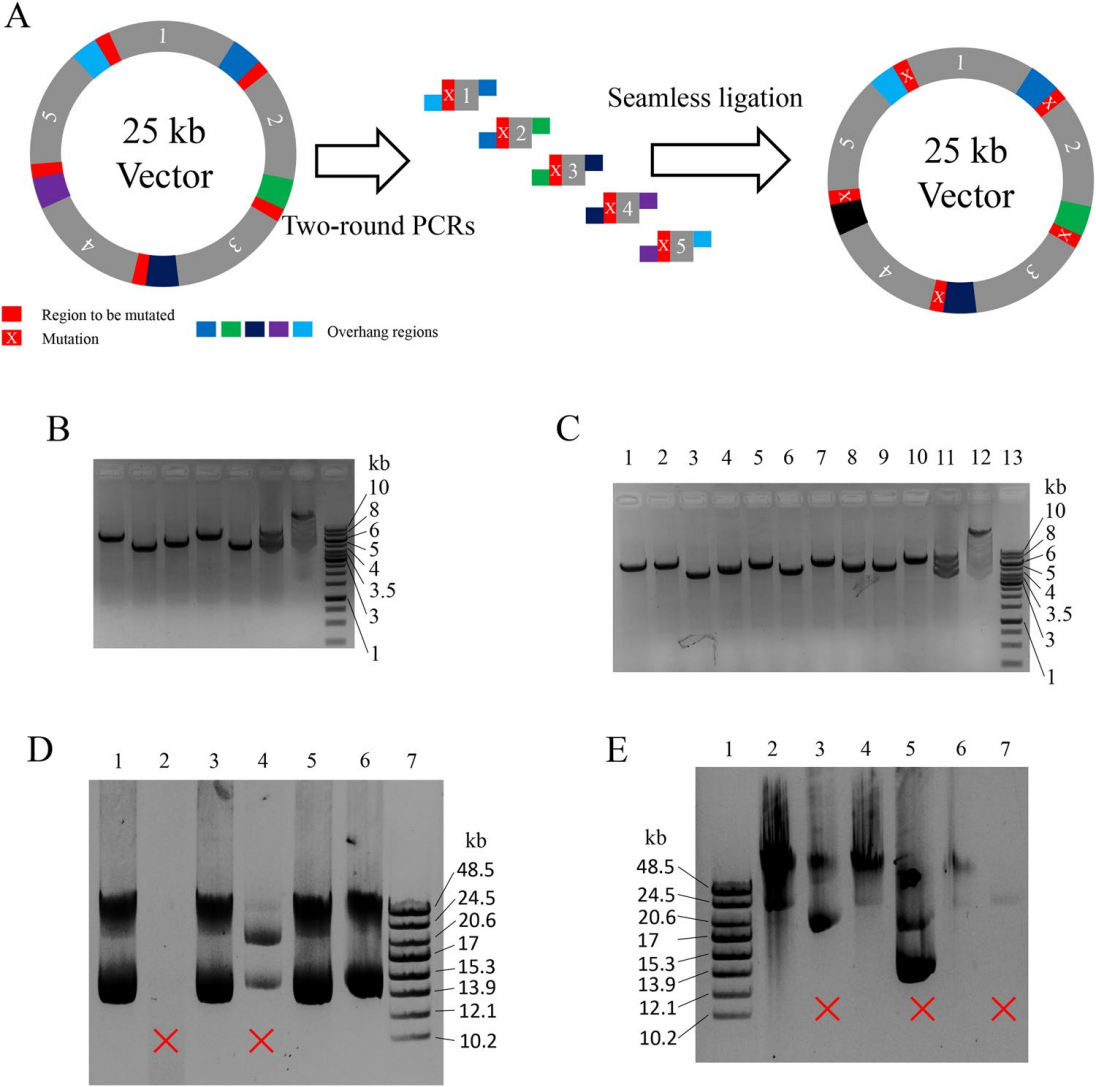
Mutations in larger plasmids. (**A**) Schematic representation of LFEAP mutagenesis in large plasmids. The first-round PCRs cut large plasmids into small pieces (~5 kb) with mutagenic ends. The second-round PCRs and the subsequent annealing yield multi-part DNAs with sticky ends, which can be seamlessly joined by T4 DNA ligase simultaneously. (**B**) Introduction of mutations in a 25 kb plasmid. Electrophoresis on a 1% agarose gel shows the DNA products generated by the procedure described in the Supplementary Methods. Lanes 1–5: DNA fragments 1 to 5 generated by first-round PCRs. Lane 6: mixture of annealed multi-part DNAs with sticky ends generated by second-round PCRs and the subsequent annealing. Lane 7: the mixture as shown in lane 6 treated with T4 DNA ligase. Lane 8: 1 kb DNA ladder. (**C**) Introduction of mutations in a 50 kb plasmid. Electrophoresis on a 1% agarose gel shows the DNA products generated by the procedure shown in the Supplementary Methods. Lanes 1–10: DNA fragments 1 to 10 generated by first-round PCRs. Lane 11: mixture of annealed multi-part DNAs with sticky ends generated by second-round PCRs and the subsequent annealing. Lane 12: the mixture as shown in lane 11 treated with T4 DNA ligase. Lane 13: 1 kb DNA ladder. (**D**) Electrophoresis on a 0.5% agarose gel of a 25 kb plasmid. Lane 1: 25 kb plasmid before introducing mutations. Lanes 2–6: 25 kb plasmids after introducing mutations propagated from five single colonies. Lane 7: GeneRuler high range DNA ladder (Thermo Fisher Scientific). Incorrect patterns are marked with a ‘×’. (**E**) Electrophoresis on a 0.5% agarose gel of a 50 kb plasmid. Lane 1: GeneRuler high range DNA ladder (Thermo Fisher Scientific). Lane 2: 50 kb plasmid before introducing mutations. Lanes 2–7: 50 kb plasmids after introducing mutations propagated from five single colonies. Incorrect patterns are marked with a ‘×’. The full-length agarose gels of 25 kb and 50 kb plasmids are presented in Supplementary Figure S11.

As almost DNA polymerases cannot amplify long templates with high efficiency and fidelity, LFEAP mutagenesis uses a new strategy in which the large DNA is split into small pieces. The procedure for introducing mutations into large plasmids (Fig. 5A) was similar to that for introducing multiple-site mutations (Fig. 2). The first-round PCR cut the large plasmid into small pieces (~5 kb each) with mutagenic ends, followed by the second-round PCR and the subsequent annealing that yielded DNA fragments with compatible ends. These were simultaneously joined to each other using T4 DNA ligase, yielding the mutagenized plasmid.

As examples, we first performed experiments to create five point mutations in a 25 kb plasmid (see Supplementary Figure S5 for the plasmid structure and the primer design, and Supplementary Information for the plasmid sequence) with such an approach (see Supplementary Methods for the detailed experimental procedure). Accordingly, this 25 kb plasmid was cut into five small fragments (6, 4, 5, 6, and 4 kb) in the first-round PCRs (Fig. 5B, lanes 1, 2, 3, 4, and 5). After treating with PNK, the DNA products generated by the second-round PCR and the subsequent annealing were mixed at a 1:1:1:1:1 molar ratio of fragments 1 to 5 (Fig. 5B, lane 6), followed by ligation to seal nicks between each fragment, causing the DNA band to shift upwards to a higher molecular weight on the agarose gel (Fig. 5B, lane 7). After transforming chemically competent *E. coli* host cells with these ligated DNAs, 1,848 ± 165 CFUs (n = 3) per µg of DNA were obtained. To evaluate the fidelity of LFEAP mutagenesis for a 25 kb plasmid, we randomly chose 20 colonies from each transformation and isolated using the QIAGEN^®^ Large-Construct Kit. We then performed DNA electrophoresis of these 25 kb plasmids before and after introducing mutations by LFEAP to separate those damaged during the cloning procedure. The plasmids were propagated from single colonies. About 60% of newly constructed plasmids were damaged (Fig. 5D, six isolated plasmids are shown). The positive plasmids shown on the agarose gel were chosen and further confirmed by DNA sequencing of full DNA plasmid or each joining site (Supplementary Figure S6 and Supplementary Figure S7). Few unwanted mutations were found in the plasmids after introducing mutations by LFEAP mutagenesis (~70% fidelity), and most unwanted mutations were found within the joining sites.

Furthermore, we performed experiments to create a point mutation in a 50 kb plasmid (see Supplementary Figure S8 for the plasmid structure and the primer design, Supplementary Information for the plasmid sequence, and Supplementary Methods for the detailed experimental procedure). Accordingly, this 50 kb plasmid was cut into 10 DNA fragments (6, 6, 4, 5, 5.5, 6, 6, 5, 5, and 6) in the first-round PCR (Fig. 5C, lane 1, 2, 3, 4, 5, 6, 7, 8, 9, and 10, respectively). The second-round PCR and the subsequent annealing yielded DNA products with overhang sequences that were joined up by T4 DNA ligase (Fig. 5C, lane 12). After transformation, 526 ± 58 CFUs (n = 3) per µg of DNA were obtained. We randomly chose 20 colonies from each transformation, and the plasmids propagated from single colonies were isolated and then subjected to agarose gel electrophoresis (Fig. 5E, six isolated plasmids are shown). The positive plasmids shown on the agarose gel were chosen and further confirmed by DNA sequencing of the full DNA plasmid or each joining site (Supplementary Figure S9 and Supplementary Figure S10). Similar to the 25 kb plasmids, few unwanted mutations were found in the plasmids after introducing mutations by LFEAP mutagenesis (~50%) and most unwanted mutations were found within the joining sites.

In summary, LFEAP mutagenesis is efficient in introducing mutations into large plasmids of up to 50 kb in our test conditions.

## Discussion

Our newly developed method provides several advantages over existing technologies for DNA mutagenesis. LFEAP mutagenesis is based on a two-round PCR procedure, followed by ligation of the resulting DNA fragments. The primers for LFEAP mutagenesis are designed such that all modified nucleotides and overhang regions are introduced at the 5′ end of the template annealing regions, which greatly reduces the complementary region of the primers and allows full displacement of the modified nucleotides outside the template annealing region. These strategies lead to several advantages of LFEAP mutagenesis. (1) The primer design strategy eliminates primer-dimer formation and mispriming, which ensures exponential amplification for high PCR efficiency and facilitates the introduction of long mutation sequences. (2) LFEAP mutagenesis uses linear PCR to generate overhang cohesive ends for direct ligation; hence only the most common lab enzymes like high-fidelity DNA polymerase, T4 DNA ligase, and PNK are required. No more special enzymes, plasmids, kits, or host strains are required. (3) Our two-round PCR design dilutes parental templates, which reduces the background and improves the efficiency of mutagenesis. (4) LFEAP mutagenesis efficiently assembles the modified DNA fragments *in vitro* by a traditional ligation reaction that can be accurately manipulated and monitored by agarose gel electrophoresis directly.

Furthermore, LFEAP mutagenesis provides a versatile method for handling different types of mutagenesis, such as point mutations, insertions, deletions, substitutions, and multiple-site changes. While most widely used PCR-based mutagenesis methods, such as QuikChange site-directed mutagenesis and its variations, are effective for producing single or a few nucleotide changes in a small plasmid, larger or multiple-site changes are more difficult^4,9–12,30^. LFEAP mutagenesis overcomes this limitation and can incorporate large nucleotides changes since the modified nucleotides are introduced at the 5′ end of the template annealing regions rather than in the middle of longer mutagenic primers. While many strategies, based on either homologous recombination^24,31^ or OE^4,15,16^, have been reported and developed for multiple-site mutations, their efficiency and fidelity drop precipitously when more than five sites are targeted simultaneously. LFEAP mutagenesis can simultaneously create up to 15 mutations with higher efficiency since the assembly of each fragment with desired mutations is guided by overhang adapter sequences, which greatly improves assembly efficiency, and hence mutagenesis efficiency.

LFEAP mutagenesis also offers an efficient method to introduce mutations into large plasmid. Introduction of mutations in larger plasmids is a slow and labor-intensive process, especially for multiple mutations^9^. Low efficiency is one of the limitations for long-range PCR due to the high error rate. Most commercially available DNA polymerases can only amplify DNA up to 20 kb with high fidelity. To overcome this limitation, LFEAP mutagenesis splits large plasmids into small pieces that are within the range of recommended values for most high-fidelity polymerases for maintaining the accurate DNA sequence during amplification. The mutations are added to the 5′ ends of the resulting DNA fragments, which are then joined up with overhang adapter sequences yielding plasmid with the desired mutations. Our experiments show that this strategy has high efficiency and fidelity for creating changes in large plasmids.

One of the limiting factors in LFEAP mutagenesis is the PCR itself. LFEAP mutagenesis requires amplification of the entire plasmid, which may introduce unwanted mutations by off-target polymerase errors, especially when working with large plasmids. In our experience, plasmid truncations are sometimes found in large plasmids (Fig. 5D and E). This is common in plasmids over 10 kb and inevitable because larger plasmids are likely to be damaged during purification and handling^32^. LFEAP mutagenesis requires large plasmids to be divided into small DNA fragments of 4–6 kb. Most commercially available high-fidelity DNA polymerases can perform PCR in this range with ultra-low error rates (e.g., 4.4 × 10^−7^ for Phusion^®^ High-Fidelity DNA Polymerase as reported by Finnzymes/Thermo Scientific). For our method, most of the DNA operations are concentrated in joining site zones. These characteristics of LFEAP mutagenesis may explain why lower unwanted mutation rates were found in the plasmids after introducing mutations by LFEAP mutagenesis and most unwanted mutations were found in the joining sites. Hence, our method is sufficient for most routine mutagenesis. The other disadvantage associated with LFEAP mutagenesis is that it needs two rounds of PCR. Luckily, primer synthesis is no longer costly, and the extra time required for a second PCR reaction is compensated for since there is no need for treatment with restriction enzymes, with is time consuming. Due to high stability and efficiency, we always obtained the desired mutants in one attempt, there by saving time and labor.

**Figure 6:**
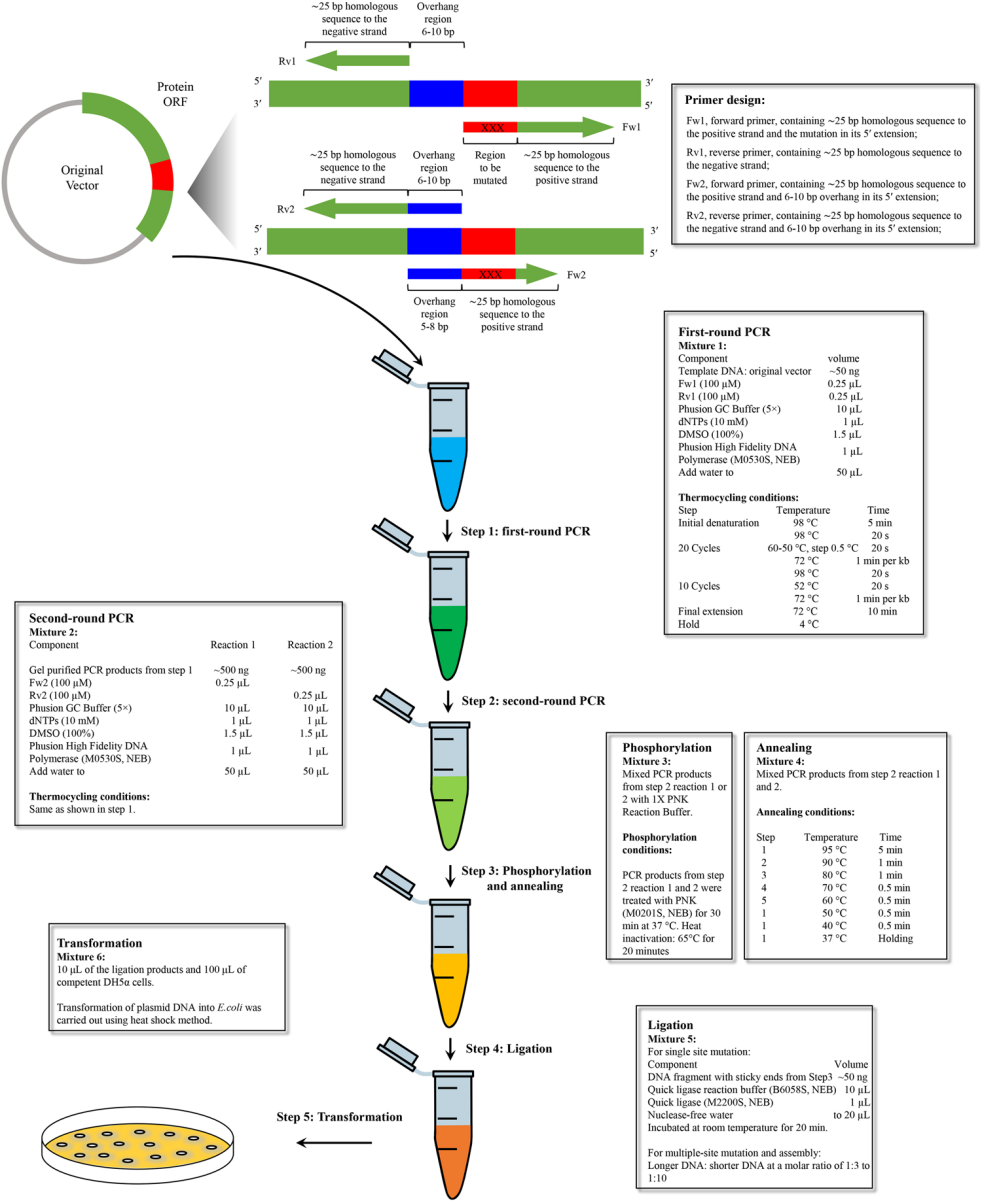
Schematic of the LFEAP mutagenesis protocol for generating single-site mutation in plasmid DNA. A detailed overview of the primer design, mutagenesis procedure, and experimental conditions is shown.

In short, we developed a simple, robust, and reliable method for creating a variety of mutations. Figure 6 summarizes the detailed protocol for the generation of single-site mutations with LFEAP mutagenesis. Multiple-site plasmid mutagenesis, as well as mutagenesis in large plasmids, can be achieved easily with high efficiency and fidelity by following the appropriate modifications of this protocol.

## Materials and Methods

### *E. coli* strains, primers, plasmids, and reagents

Host strain *E. coli* DH5α was obtained from Invitrogen Corporation (Carlsbad, CA, USA). The competent DH5α cells were prepared using the calcium chloride method^33^. Bacteria containing plasmids were cultured in lysogeny broth (LB) medium^34^ with appropriate antibiotics (kanamycin or ampicillin at 50 or 100 μg/ml, respectively). All the primers used were commercially synthesized by Invitrogen Corporation (Carlsbad, CA, USA). The vectors, pET22b and pcDNA™ 3.1 (+) were obtained from Invitrogen Corporation (Carlsbad, CA, USA). The vectors, pNGFP-BC, pCGFP-BC, pNGFP-EU, and pCGFP-EU were courtesy of Dr. Eric Gouaux. Phusion^®^ high-fidelity DNA polymerase, DNA marker, Taq DNA polymerase, T4-PNK, and T4 DNA ligase were purchased from New England Biolabs (Ipswich, MA, USA). Human cDNA library was purchased from Clontech Laboratories (Mountain View, CA, USA). QIAquick PCR purification kit, QIAquick gel extraction kit, QIAprep spin miniprep kit, and Large-Construct Kit were purchased from Qiagen (Hilden, Germany).

### PCR and ligation

The primer sequences used in this study were listed in Supplementary Table S1. Unless otherwise stated, 50 μl PCR reactions were performed using Phusion^®^ high-fidelity DNA polymerase (New England Biolabs). The PCR conditions are listed in Supplementary Table S2 and Supplementary Table S3. The resulting PCR products in the first round were separated via 1% agarose gel electrophoresis. The complementary DNA products from second-round PCRs were annealed without purification (see Supplementary Table S4 for annealing conditions). DNA ligation reactions were performed to join up DNA fragments with complementary sticky ends in a final volume of 20 μl using T4 DNA ligase following the standard protocol from New England Biolabs. In brief, the longer and shorter DNA fragments were mixed at a molar ratio of 1:3–1:10. The reaction was incubated at room temperature for 2 h. After heat inactivation at 65 °C for 10 min, the reaction was chilled on ice.

### Plasmid transformation, isolation, and sequencing

Transformation of the ligated DNA products into *E. coli* was carried out using the heat shock method. In brief, 10 μl of the ligation products and 100 μl of competent DH5α cells were mixed and incubated for 15 min on ice, and subsequently heat shocked at 42 °C for 1 min and then placed back on ice. LB media (500 μl) was added, and the transformed cells were incubated at 37 °C for 60 min with agitation. After incubation, cells were pelleted and resuspended in 100 µl flash LB, which was then spread on LB agar plates containing ampicillin (100 μg/ml) or kanamycin (50 μg/ml). The plates were incubated overnight at 37 °C. The resulting colonies were then counted to determine the efficiency of mutagenesis reactions. Ten to twenty colonies were randomly selected from each transformation, and the plasmids were isolated using the QIAprep Spin Miniprep Kit or Large-Construct Kit. DNA sequencing was performed to assess the fidelity of the mutagenesis reaction.

### Determining optimal overhang size needed for the LFEAP mutagenesis method

Primers were designed for the addition of two nucleotides (TA) in the middle of the *Xho*I restriction site (CTCGAG) in pcDNA3.1 (+)-*MCM6* plasmid (Fig. 3A), thereby disrupting the restriction site^29^. The overhang size was varied from 0 to 20 bp (see Supplementary Table S1 for primer sequences). The mutations were carried out by LFEAP mutagenesis (Fig. 1A). The efficiency of mutagenesis reaction as the function of overhang size was determined by counting the resulting bacterial colonies from each transformation. Ten colonies were randomly selected from each transformation, and the plasmids were isolated using the QIAprep Spin Miniprep Kit. The fidelity of mutagenesis reaction as the function of overhang size was measured by sequencing the mutated sites.

### Statistical analysis

Data points represent the mean from three independent experiments, and, where indicated, error bars represent one standard deviation from the mean.

## Electronic supplementary material

Supplementary DATA

Supplementary Dataset 1

Supplementary Dataset 2
