# Supplementary material for: Efficient strategy for introducing large and multiple changes in plasmid DNA
Source: Sci Rep. 2018 Jan 29;8:1714. doi: 10.1038/s41598-018-20169-8 (PMC5789069; doi:10.1038/s41598-018-20169-8)
Supplement: Supplementary file 1 — Supplementary DATA [file 41598_2018_20169_MOESM1_ESM.pdf]

SUPPLEMENTARY DATA

Efficient strategy for introducing large and multiple changes in plasmid DNA

Fanli Zeng[1], Suhua Zhang[2], Zhimin Hao[1], Shixin Duan[1], Yanan Meng[1], Pan Li[1], Jingao Dong[1*], and Yibin Lin[3*]

*Correspondence: Yibin.lin@uth.tmc.edu; shmdjg@hebau.edu.cn

[1] College of Life Sciences, Hebei Agricultural University, Baoding, 071001, China;

[2] Institute of Biophysics, Hebei University of Technology, Tianjin, 300401, China;

[3] Department of Biochemistry and Molecular Biology, University of Texas Health Science Center at Houston McGovern Medical School, 6431 Fannin Street, Houston, TX 77030, USA.

■ SUPPLEMENTARY METHODS

Basic mutations. Point mutations, substitutions, deletions, and insertions were performed followed the procedures as shown in Fig. 1A. As an example, we described the detailed procedure for creating point mutation of *yaaU* (R205A). To introduce *yaaU* (R205A) in pNGFP-BC-*yaaU* plasmid, one PCR was performed used primer pair YAAU-R205Afw1 and YAAU-R205Afw1 (see Supplementary Table S1 for details) with the PCR conditions as listed in Supplementary Table S2 and Supplementary Table S3. The resulting PCR product was gel purified and used as template in the second-round PCR. In the second-round PCR, two single-primer PCRs in parallel were performed to generate two complementary single-stranded DNA fragments with single primer of YAAU-R205Afw2 and YAAU-R205Afw2. The generated complementary single-stranded DNA fragments were 5' phosphorylated by T4 polynucleotide kinase followed the standard protocol from NEB, and subsequently annealed with the conditions listed in Supplementary Table S4 to form double-stranded DNA fragments with overhang adapter sequences at their 5' ends. The annealed DNA fragment was treated with T4 ligase (NEB) followed the standard ligation protocol from NEB. After ligation, DNA sample was transformed into chemical competent *E.coli* cells.

Multiple-site mutations. Multiple-site mutations were performed by following the procedure as shown in Fig.2. To introduce three point mutations (E52A, R309A, and Q668A) in *MCM6* gene in plasmid of pNGFP-EU-*MCM6*, three PCRs in parallel were performed used pNGFP-EU-*MCM6* as template and primer pairs: mcm6-q668a-1fw/mcm6-e52a-1rv, mcm6-e52a-1fw/mcm6-r309a-1rv, and mcm6-r309a-1fw/mcm6-q668a-1rv (see Supplementary Table S1 for the details) with the PCR conditions as listed in Supplementary Table S2 and Supplementary Table S3. The resulting PCR products (fragment 1, fragment 2, and fragment 3) were gel purified and used as templates in the second-round PCR. In the second-round PCR, two single-primer PCRs in parallel were performed to generate two complementary single-stranded DNA fragments with single primer of mcm6-q668a-2fw or mcm6-e52a-2rv, mcm6-e52a-2fw or mcm6-r309a-2rv, and mcm6-r309a-2fw or mcm6-q668a-2rv (see Supplementary Table S1 for the details). The generated complementary single-stranded DNA fragments were 5' phosphorylated by T4 polynucleotide kinase, and subsequently annealed with the conditions listed in Supplementary Table S4 to form double-stranded DNA fragments with overhang adapter sequences at their 5' ends. Three annealed DNA fragments were mixed at the molar ratio of 1:3:3 and treated with T4 ligase (NEB) followed the standard ligation protocol from NEB. After ligation, DNA samples were transformed into chemical competent *E.coli* cells.

Six, ten, fifteen point mutations were performed following the same procedure as described above. The detail primer sequences used were listed in Supplementary Table S1.

Introduction of five point mutations in 25 kb plasmid. The introduction of five point mutations in 25 kb plasmid was performed by following the procedure as shown in Fig.2 and Fig.5A. A pET22b vector, between the start codon ATG (287), and the sequence (157) CACCACCACCACCACCAC, inserted with a 20 kb genomic DNA fragment from *E. coli* (200482-220928) containing 21 genes (Supplementary Table S6), was used to introduce five point mutations within the gene cluster (see Supplementary Figure S5 for detailed plasmid structure and Supplementary Information for the plasmid sequence). The primers used for first-round PCR were designed such that the mutations will be added into the 5' ends of resulting PCR products and the primers used for second-round PCR were designed such that overhang adapter sequence will be added into 5' ends of resulting PCR products (see Supplementary Figure S6 for details primers design and sequences). In the first-round PCR, five PCRs were performed used this 25 kb plasmid as template and primer pairs 25-1-1fw/25-1-1rv, 25-2-1fw/25-2-1rv, 25-3-1fw/25-3-1rv, 25-4-1fw/25-4-1rv, and 25-5-1fw/25-5-1rv with the PCR conditions as listed in Supplementary Table S2 Supplementary Table S3. The resulting DNA products were gel purified and used as templates in the second-round PCR. In the second-round PCR, two single-primer PCRs in parallel were performed to generate two complementary single-stranded DNA fragments with single primer of 25-1-2fw or 25-1-2rv, 25-2-2fw or 25-2-2rv, 25-3-2fw or 25-3-2rv, 25-4-2fw or 25-4-2rv, and 25-5-2fw or 25-5-2rv with the PCR conditions as listed in Supplementary Table S2 and Supplementary Table S3. Resulting complementary single-stranded DNA fragments were 5' phosphorylated by T4 polynucleotide kinase, and then subsequently annealed at the molar ratio of 1:1:1:1:1 without further purification with the conditions listed in Supplementary Table S4. The annealed multi-part DNAs with nicks were sealed by T4 ligase followed the standard protocol from NEB. After ligation, DNA samples were transformed into chemical competent *E.coli* cells.

Introduction of a point mutation in 50 kb plasmid. The introduction of a point mutation in 50 kb plasmid was performed by following the procedure as shown in Fig.2 and Fig.5A. A pET22b vector, between the start codon ATG (287), and the sequence (157) CACCACCACCACCACCAC, inserted with a 45 kb genomic DNA fragment from *E. coli* (200482-248134) containing 46 genes (Supplementary Table S7), was used to introduce a point mutation, ldcC Q32A within the gene cluster (see Supplementary Figure S7 for plasmid structure). The two nucleotides (GC) were introduced to replace CA present in the original vector (AGGCTTT<u>CA</u>GATTATCTGG) such that the resulting sequence (AGGCTTT<u>GC</u>GATTATCTGG) leaves a mutant of ldcC Q32A in the plasmid. Supplementary Figure S7 shows all primers used in two-round PCRs. In the first-round PCR, 10 PCRs were performed used

this 50 kb plasmid as template and primer pairs 50-1-1fw/50-1-1rv, 50-2-1fw/50-2-1rv, 50-3-1fw/50-3-1rv, 50-4-1fw/50-4-1rv, 50-5-1fw/50-5-1rv, 50-6-1fw/50-6-1rv, 50-7-1fw/50-7-1rv, 50-8-1fw/50-8-1rv, 50-9-1fw/50-9-1rv, and 50-10-1fw/50-10-1rv with the PCR conditions as listed in Supplementary Table S2 Supplementary Table S3. The resulting DNA products were gel purified and used as templates in the second-round PCR. In the second-round PCR, two single-primer PCRs in parallel were performed to generate two complementary single-stranded DNA fragments with single primer of 50-1-2fw or 50-1-2rv, 50-2-2fw or 50-2-2rv, 50-3-2fw or 50-3-2rv, 50-4-2fw or 50-4-2rv, 50-5-2fw or 50-5-2rv, 50-6-2fw or 50-6-2rv, 50-7-2fw or 50-7-2rv, 50-8-2fw or 50-8-2rv, 50-9-2fw or 50-9-2rv, and 50-10-2fw or 50-10-2rv with the PCR conditions as listed in Supplementary Table S2 and Supplementary Table S3. Resulting complementary single-stranded DNA fragments were 5' phosphorylated by T4 polynucleotide kinase, and then subsequently annealed at the molar ratio of 1:1:1:1:1:1:1:1:1:1 without further purification with the conditions listed in Supplementary Table S4. The annealed multi-part DNAs with nicks were sealed by T4 ligase followed the standard protocol from NEB. After ligation, DNA samples were transformed into chemical competent *E.coli* cells.

■ SUPPLEMENTARY FIGURES

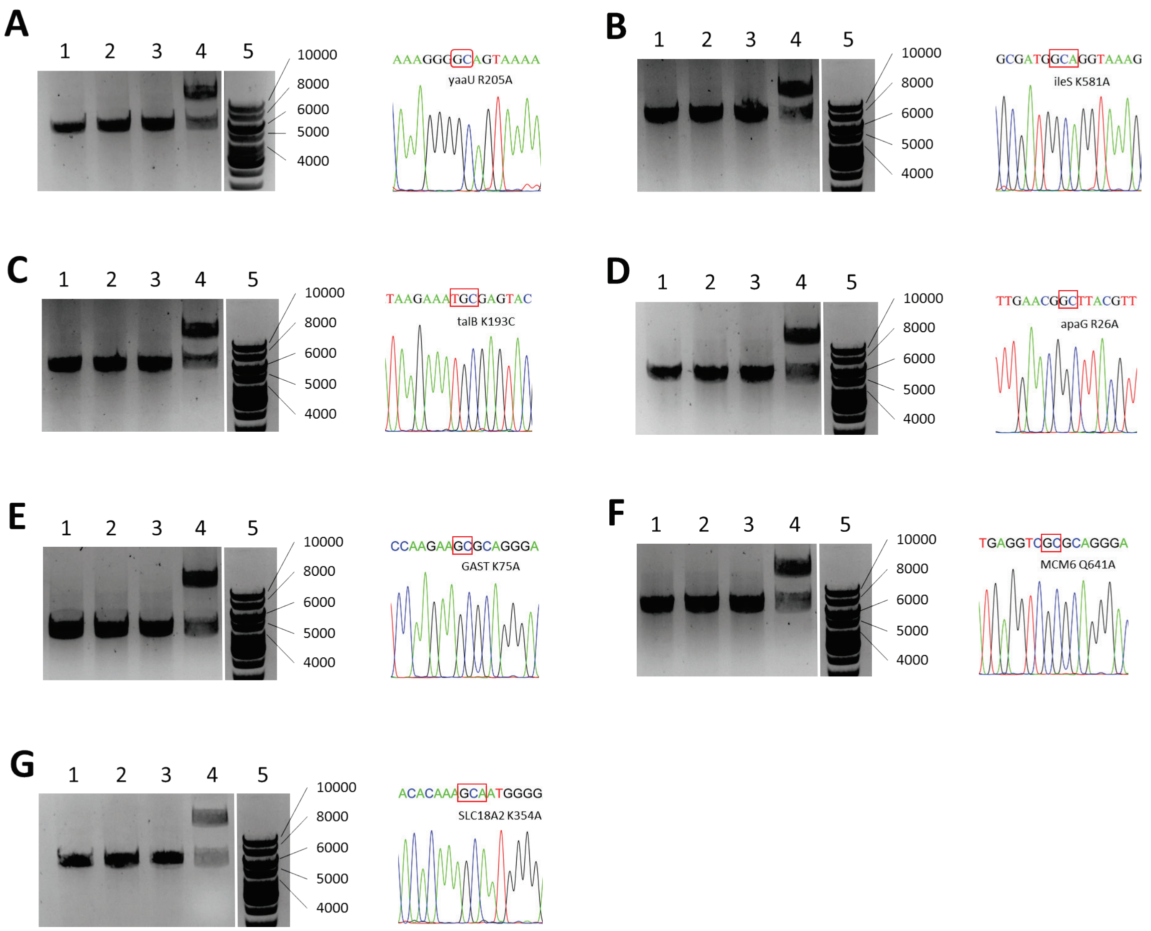

Supplementary Figure S1. Site-directed point mutations. Agarose electrophoresis resulting (right panel) from the amplification using the primers as listed at Supplementary Table S1 and DNA sequencing confirmations of the mutation sites (left panel). The red boxes show the mutation sites. Lane 1: first-round PCR product using Fw1 and Rv1 primers; Lane 2: Annealing of PCR products with Fw2 or Rv2 primer using DNA shown in lane 1 as template; Lane 3: DNA sample shown in lane 2 before ligation; Lane 4: DNA sample shown in lane 2 after ligation; lane 5: 1 kb DNA ladder. (A) yaaU R205A. (B) ileS K581A. (C) talB K193C. (D) apaG R26A. (E) GAST K75A. (F) MCM6 Q641A. (G) SLC18A2 K354A. DNA samples were electrophoresed in 1% agarose gel.

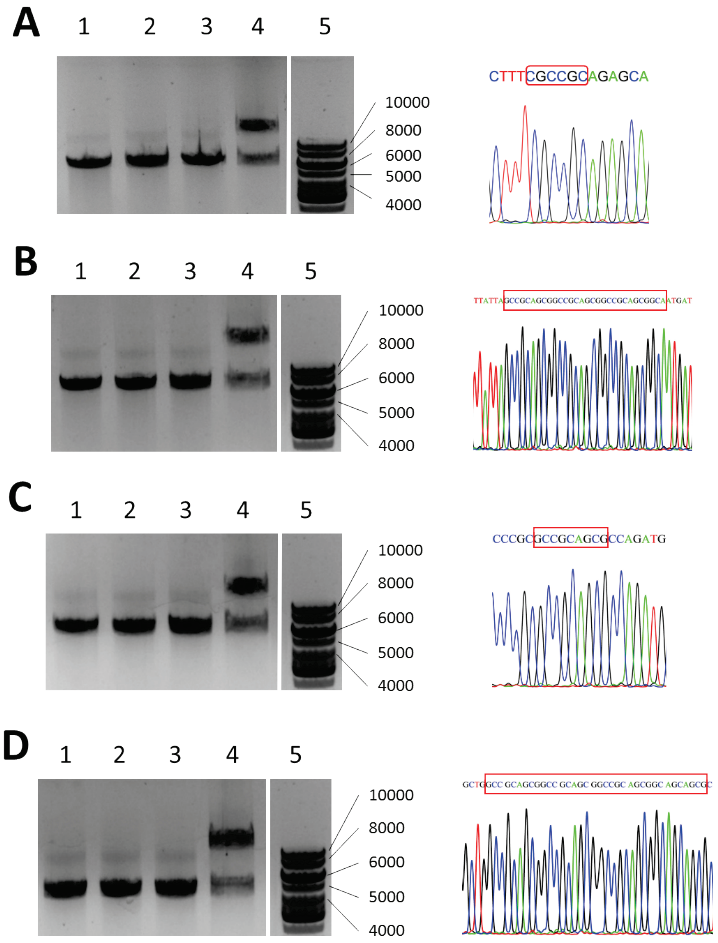

Supplementary Figure S2. Substitutions. Agarose electrophoresis resulting (left panel) from the amplification using the primers as listed at Supplementary Table S1 and DNA sequencing confirmations of the mutation sites (right panel). The red boxes show the mutation sites. Lane 1: first-round PCR product using Fw1 and Rv1 primers; Lane 2: Annealing of PCR products with Fw2 or Rv2 primer using DNA shown in lane 1 as template; Lane 3: DNA sample shown in lane 2 before ligation; Lane 4: DNA sample shown in lane 2 after ligation; Lane 5: 1 kb DNA ladder. (A) YAAU DE224AA. (B) YAAU RKGRVKECEE202AAAAAAAAAA. (C) GAST SQQ27AAA. (D) GAST EQQGPASHHRRQ48AAAAAAAAAAAA. DNA samples were electrophoresed in 1% agarose gel. Red boxes show the mutation sites.

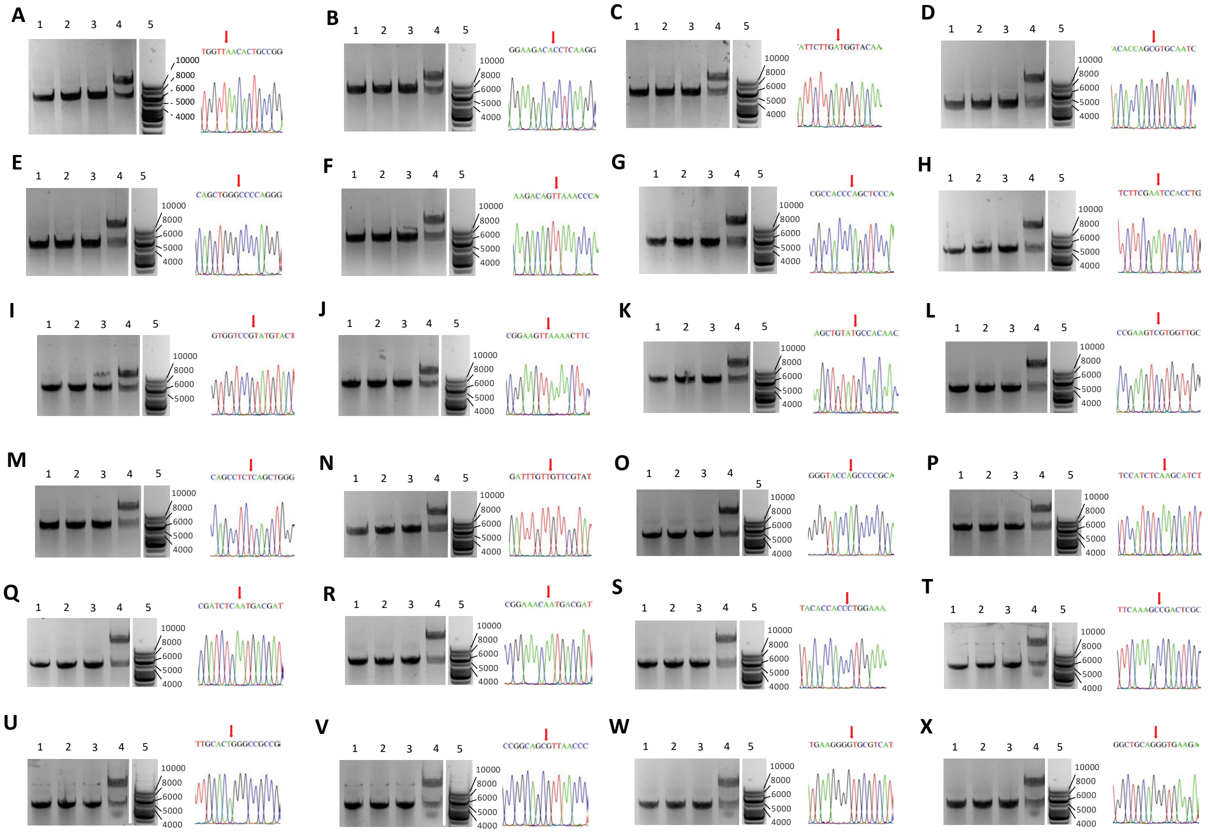

Supplementary Figure S3. Deletions. Agarose electrophoresis resulting (left panel) from the amplification using the primers as listed at Supplementary Table S1 and DNA sequencing confirmations of the mutation sites (right panel). The red arrows show the mutation sites. Lane 1: first-round PCR product using Fw1 and Rv1 primers; Lane 2: Annealing of PCR products with Fw2 or Rv2 primer using DNA shown in lane 1 as template; Lane 3: DNA sample shown in lane 2 before ligation; Lane 4: DNA sample shown in lane 2 after ligation; Lane 5: 1 kb DNA ladder. (A) yaaU del 909A. (B) ileS del 2096T. (C) talB del 552C. (D) apaG del 253G. (E) GAST del 183A. (F) MCM6 del 1745T. (G) PRRT2 del 1741C. (H) SLC18A2 del 1415G. (I) yaaU del F28-Gly31. (J) ileS del E201-D204. (K) talB del Q28-D31. (L) apaG del Q63-G66. (M) GAST del H55-R58. (N) MCM6 del D202-K205. (O) PRRT2 del D43-E45. (P) SLC18A2 del D73-Q76. (Q) yaaU del K11-N434. (R) ileS del G14-A929. (S) talB del V14-K308. (T) apaG del V14-F116. (U) GAST del V14-F116. (V) MCM6 del Q14-V812. (W) PRRT2 del V14-S331. (X) SLC18A2 del E14-I505. DNA samples were electrophoresed in 1% agarose gel. Red arrows show the mutation sites.

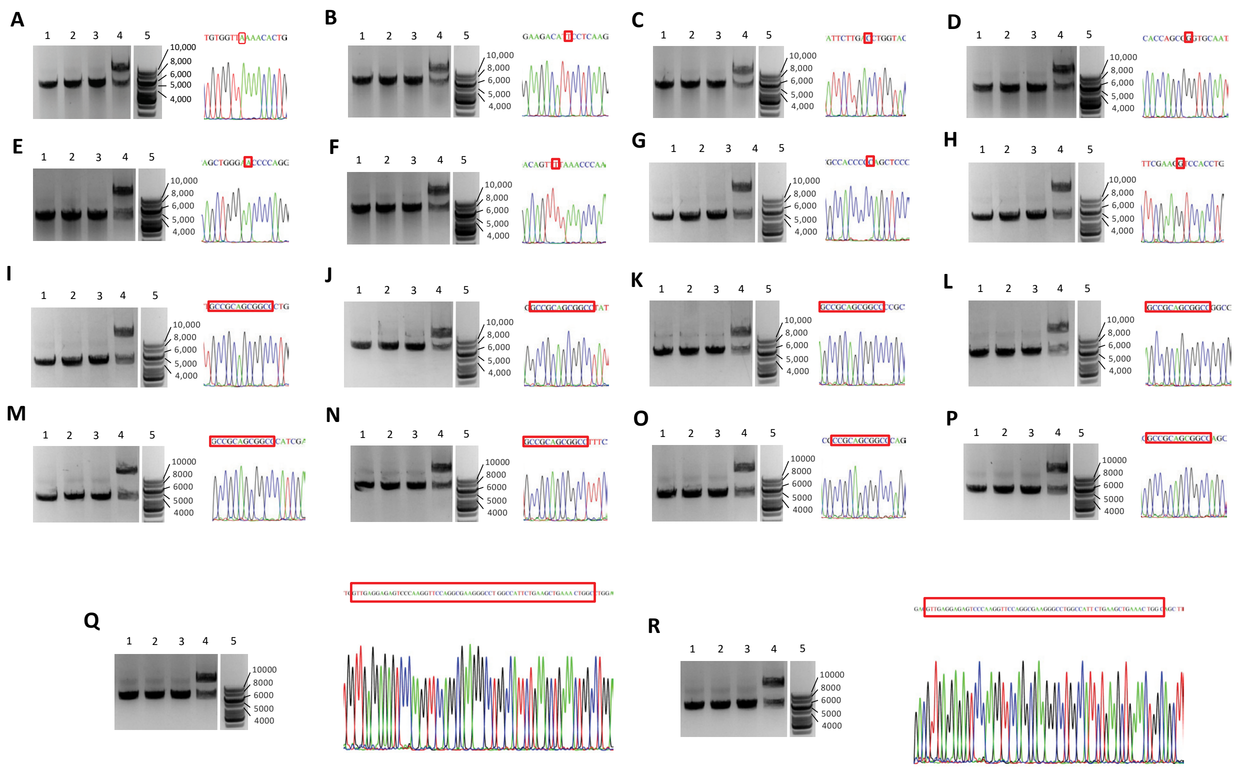

Supplementary Figure S4. Insertions. Agarose electrophoresis resulting (left panel) from the amplification using the primers as listed at Supplementary Table S1 and DNA sequencing confirmations of the mutation sites (right panel). The red boxes show the mutation sites. Lane 1: First-round PCR product using Fw1 and Rv1 primers; Lane 2: Annealing of PCR products with Fw2 or Rv2 primer using DNA shown in lane 1 as template; Lane 3: DNA sample shown in lane 2 before ligation; Lane 4: DNA sample shown in lane 2 after ligation; Lane 5: 1 kb DNA ladder. (A) *yaaU* inserted A at 909. (B) *ileS* inserted T at 2096. (C) *talB* inserted C at 552. (D) *apaG* inserted G at 253. (E) *GAST* inserted A at 183. (F) *MCM6* inserted T at 1745. (G) *PRRT2* inserted C at 741. (H) *SLC18A2* inserted G at 1415. (I) yaaU inserted AAAA after F28. (J) ileS inserted AAAA after E201. (K) talB inserted AAAA after Q28. (L) apaG inserted AAAA after Q63. (M) GAST inserted AAAAA after H55. (N) MCM6 inserted AAAA after H55. (O) PRRT2 inserted AAAA after D43. (P) SLC18A2 inserted AAAA after D73. (Q) YAAU inserted 30 amino acids after F28. (R) SLC18A2 inserted 30 amino acids after D73. DNA samples were electrophoresed in 1% agarose gel.

A

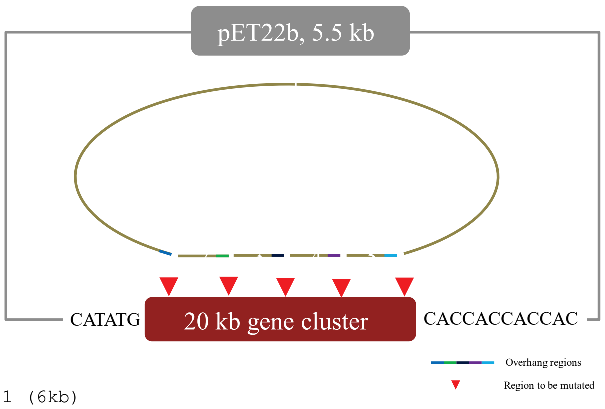

B

```
Fragment 1 (6kb)
       CGCAAAATCATGTAGCCAGACGG  25-1-1FW
CCAGCGAACCGCAAAATCATGTAGCCAGACGG  25-1-2FW
       GTTAAAATCATGTAGCCAGACGGC ···  ··· CCGAGATATCCGCACCAACGCGCAGCCCGGACTCGG
                                25-1-2RV  CTATAGGCGTGGTTGCGCGTCGGGCCTGAGCC
                                25-1-1RV  CTATAGGCGTGGTTGCGCGTCGGG
Fragment 2 (4kb)
       GCGTGGCGCGCATTGCGCCCAGC   25-2-1FW
GGACTCGGGCGTGGCGCGCATTGCGCCCAGC   25-2-2FW
       TAATGGCGCGCATTGCGCCCAGCG  ···  ··· TATCCTGGGGGCCGGTTTAATCCGCGCTCTGAAAG
                                25-2-2RV  GGACCCCCGGCCAAATTAGGCGCGAGACTTTC
                                25-2-1RV  GGACCCCCGGCCAAATTAGGCG
Fragment 3 (5kb)
       TTGATGTGCCCAACGCCCGCTTTG  25-3-1FW
GCTCTGAAAGTTGATGTGCCCAACGCCCGCTTTG  25-3-2FW
       AACATGTGCCCAACGCCCGCTTTGT ···  ··· GTGGAACTGGAGTTTGACTAATACAGGAATACTATG
                                25-3-2RV  TTGACCTCAAACTGATTATGTCCTTATGATAC
                                25-3-1RV  TTGACCTCAAACTGATTATGTCC
Fragment 4 (6kb)
       TCGCTGAATTTCCTTGATTTTG   25-4-1FW
GAATACTATGTCGCTGAATTTCCTTGATTTTG   25-4-2FW
       AGTCTGAATTTCCTTGATTTTGAA ···  ··· AGAATAATCTGGAAGTGAACTTAACCGCCTGGCAAC
                                25-4-2RV  ATTAGACCTTCACTTGAATTGGCGGACCGTTG
                                25-4-1RV  ATTAGACCTTCACTTGAATTGGC
Fragment 5 (4kb)
       GGACTTCATGATTGTGATTTCCC  25-5-1FW
CCTGGCAACGGACTTCATGATTGTGATTTCCC  25-5-2FW
       AACCTTCATGATTGTGATTTCCCG ···  ··· GACTTCAAAACCTGCGTCGGTGACGCGCCAGCGAAC
                                25-5-2RV  AGTTTTGGACGCAGCCACTGCGCGGTCGCTTG
                                25-5-1RV  AGTTTTGGACGCAGCCACTGCGC
```

Supplementary Figure S5. Introduction of five point mutations in 25 kb plasmid. A. 25 kb plasmid structure. A pET22b vector, between the start codon ATG (287), and the sequence (157) CACCACCACCACCACCAC, inserted with a 20 kb DNA fragment of *E. coli* genome (200482-220928) containing 21 genes (Supplementary Table S6), was used to introduce five point mutations within the gene cluster. B. The detailed primers design and sequences used. Mutations are highlighted with red underline.

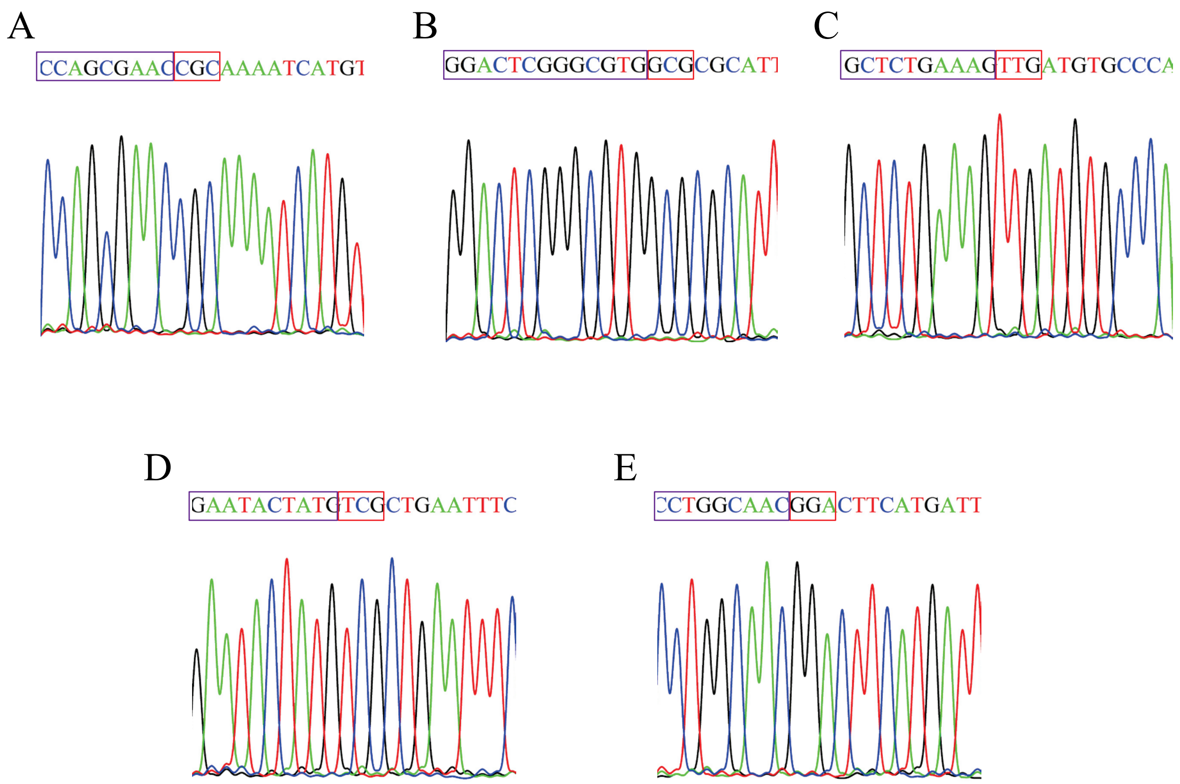

Supplementary Figure S6. DNA sequencing confirmations of the joining sites and mutations in reconstituted 25 kb plasmid. A. Joining site 1. B. Joining site 2. C. Joining site 3. D. Joining site 4. E. Joining site 5. Purple box: overhang sequences; Red box: mutations.

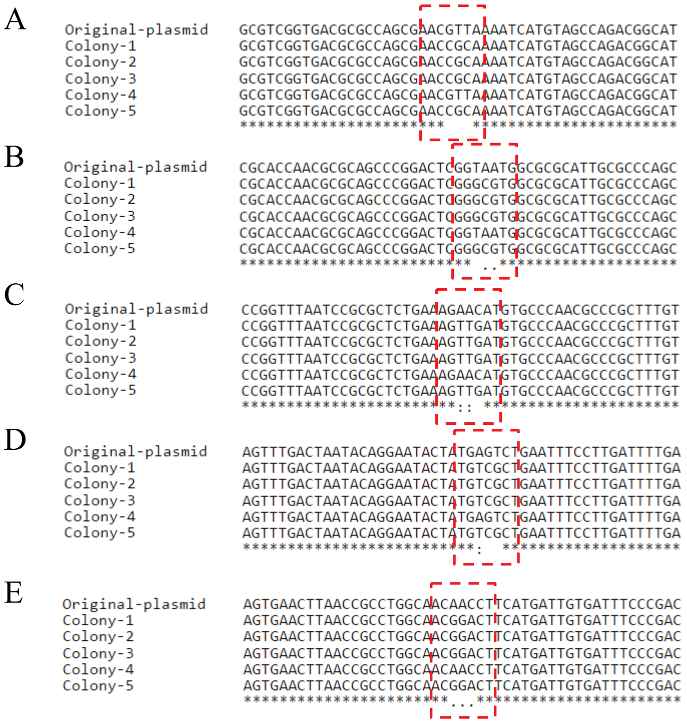

Figure S7. Sequence verification of LFEAP mutagenesis for 25 kb plasmid. Plasmids before (original-plasmid) and after (colony 1-5) introducing mutations by LFEAP that had been propagated from single colonies and checked by agarose electrophoresis to exclude those ones containing truncations were subjected to DNA sequencing. (A) Joining site 1; (B) Joining site 2; (C) Joining site 3; (D) Joining site 4; (E) Joining site 5 in the 25 kb assembly (See Supplementary Figure S5). DNA sequences were aligned by using (http://www.uniprot.org/align/). The mutations were shown in the red dashed rectangles. A full plasmid DNA sequencing result was presented in an independent file, name 25 kb DNA sequencing.jvp.

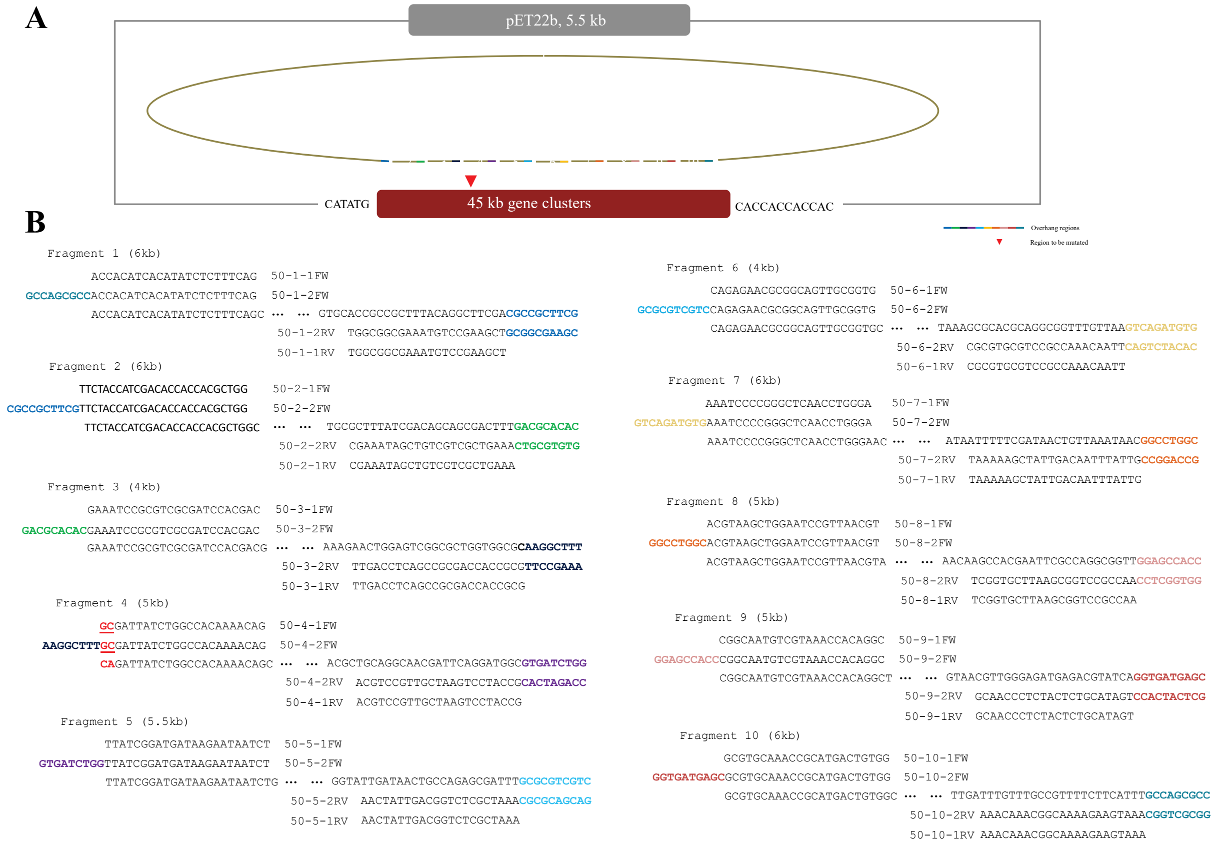

Supplementary Figure S8. Introduction of a point mutation in 50 kb plasmid. A. 50 kb plasmid structure. A pET22b vector, between the start codon ATG (287), and the sequence (157) CACCACCACCACCACCAC, inserted with a 45 kb DNA fragment of *E. coli* genome (200482-248134) containing 46 genes (Supplementary Table S7), was used to introduce a point mutation, ldcC Q32A within the gene cluster. B. The detailed primers design and sequences used. Mutations are highlighted with red underline.

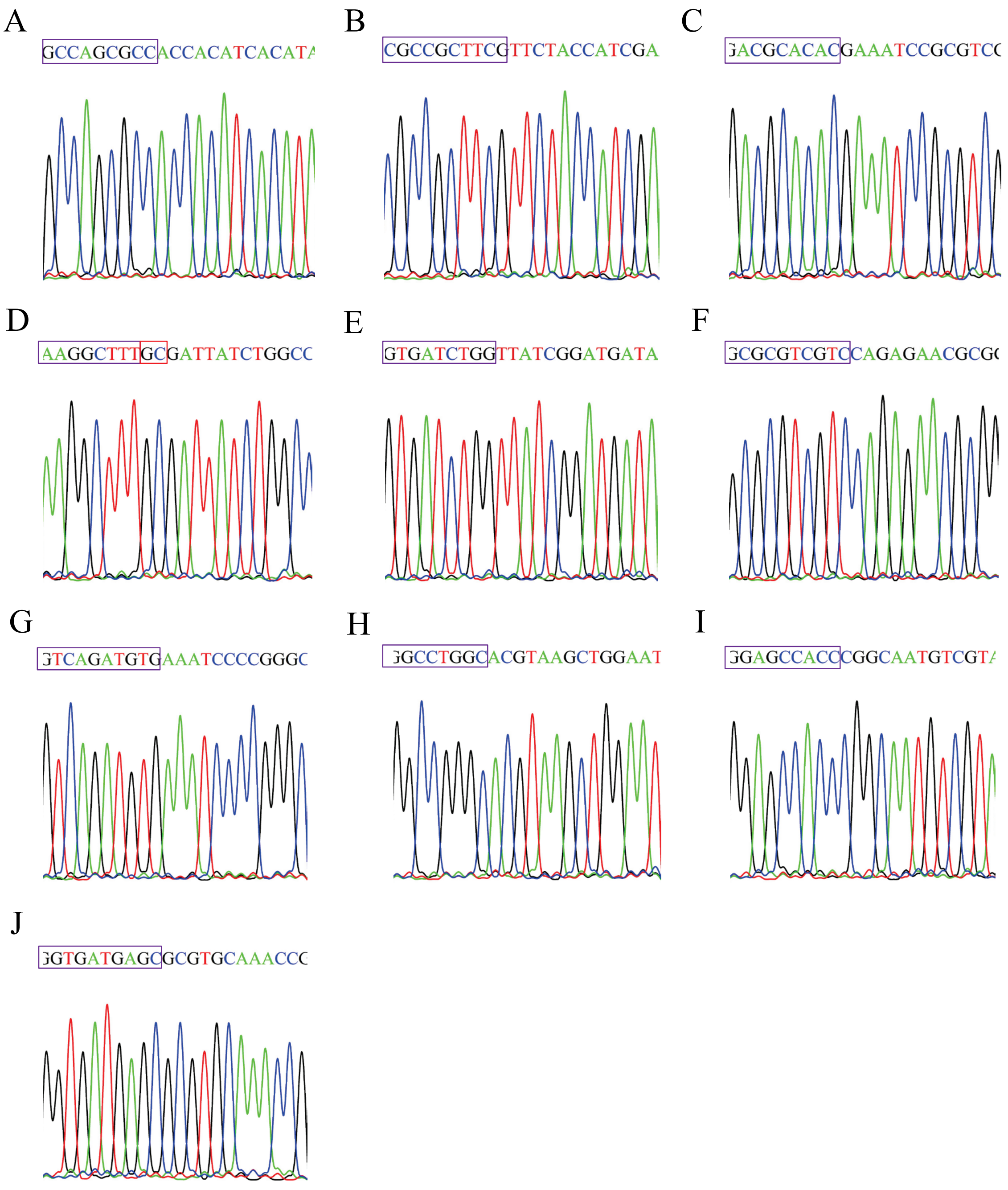

Supplementary Figure S9. DNA sequencing confirmations of the joining sites and mutation in reconstituted 50 kb plasmid. A. Joining site 1. B. Joining site 2. C. Joining site 3. D. Joining site 4. E. Joining site 5. F. Joining site 6. G. Joining site 7. H. Joining site 8. I. Joining site 9. J. Joining site 10. Purple box: overhang sequences; Red box: mutations.

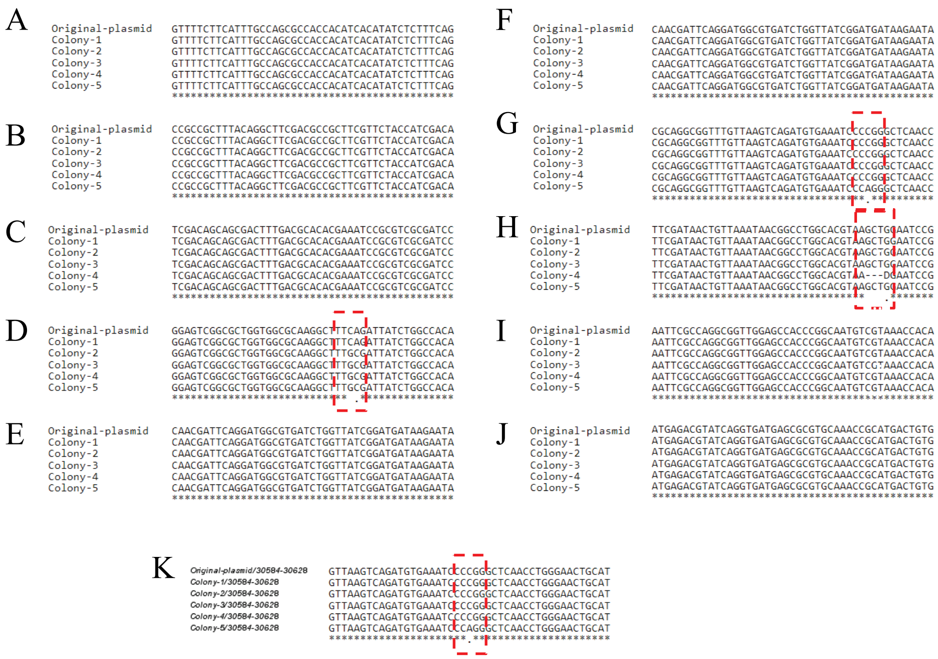

Supplementary Figure S10. Sequence verification of LFEAP mutagenesis for 50 kb plasmid. Plasmids before (original-plasmid) and after (colony 1-5) introducing mutations by LFEAP that had been propagated from single colonies and checked by agarose electrophoresis to exclude those ones containing truncations, were subjected to DNA sequencing. (A) Joining site 1; (B) Joining site 2; (C) Joining site 3; (D) Joining site 4; (E) Joining site 5; (F) Joining site 6; (G) Joining site 7; (H) Joining site 8; (I) Joining site 9; (J) Joining site 10 in the 50 kb assembly (see Supplementary Figure S8); (H) one unwanted mutation in the new constructed plasmid. DNA sequences were aligned by using (http://www.uniprot.org/align/). The mutations were shown in the red dashed rectangles. A full plasmid DNA sequencing result was presented in two independent files, name 50 kb DNA sequencing 1-25kb.jvp (for DNA sequences between 1 to 25 kb) and 50 kb DNA sequencing 25-50kb.jvp (for DNA sequencing between 25 kb to 50 kb).

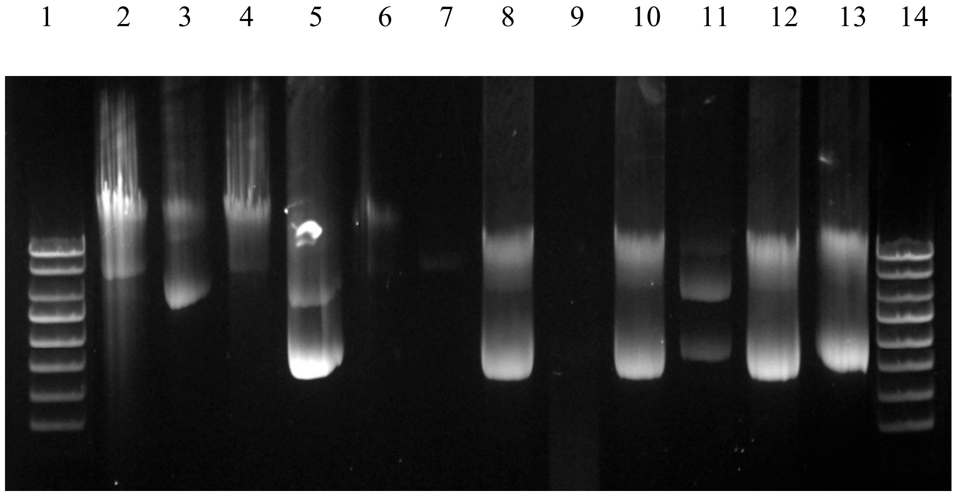

Supplementary Figure S11. Electrophoresis on a 0.5% agarose gel of 25 kb and 50 kb plasmids. Lane 1: GeneRuler high range DNA ladder (Thermo Fisher Scientific). Lane 2: 50 kb plasmid before introducing mutations. Lanes 2–7: 50 kb plasmids after introducing mutations propagated from five single colonies. Lane 8: 25 kb plasmid before introducing mutations. Lanes 9–13: 25 kb plasmids after introducing mutations propagated from five single colonies. Lane 14: GeneRuler high range DNA ladder (Thermo Fisher Scientific).

■ SUPPLEMENTARY TABLES

Supplementary Table S1. Primers used in this study

No.	Name	Primers (5'→3')*
		**Optimal overhang adapter sequence**
1	OHtestfw1	TATTCTGCAGATATCCAGCACAGTGG
2	OHtestrv1	CTCAATCTTCGAGCAAGTAGTTAG
3	OHtest2fw1	TATTCTGCAGATATCCAGCACAGTGG
4	OHtest2rv1	CTCAATCTTCGAGCAAGTAGTTAG
5	OHtest2fw2	**AA**TATTCTGCAGATATCCAGCACAGTGG
6	OHtest2rv2	**TT**CTCAATCTTCGAGCAAGTAGTTAG
7	OHtest4fw1	TATTCTGCAGATATCCAGCACAGTGG
8	OHtest4rv1	CAATCTTCGAGCAAGTAGTTAGGG
9	OHtest4fw2	**AGAA**TATTCTGCAGATATCCAGCACAGTGG
10	OHtest4rv2	**TTCT**CAATCTTCGAGCAAGTAGTTAGGG
11	OHtest6fw1	TATTCTGCAGATATCCAGCACAGTGG
12	OHtest6rv1	ATCTTCGAGCAAGTAGTTAGGGTTAAC
13	OHtest6fw2	**TGAGAA**TATTCTGCAGATATCCAGCACAGTGG
14	OHtest6rv2	**TTCTCA**ATCTTCGAGCAAGTAGTTAGGGTTAAC
15	OHtest8fw1	TATTCTGCAGATATCCAGCACAGTGG
16	OHtest8rv1	CTTCGAGCAAGTAGTTAGGGTTAAC
17	OHtest8fw2	**ATTGAGAA**TATTCTGCAGATATCCAGCACAGTGG
18	OHtest8rv2	**TTCTCAAT**CTTCGAGCAAGTAGTTAGGGTTAAC
19	OHtest10fw1	TATTCTGCAGATATCCAGCACAGTGG
20	OHtest10rv1	TCGAGCAAGTAGTTAGGGTTAAC
21	OHtest10fw2	**AGATTGAGAA**TATTCTGCAGATATCCAGCACAGTGG
22	OHtest10rv2	**TTCTCAATCT** TCGAGCAAGTAGTTAGGGTTAAC
23	OHtest12fw1	TATTCTGCAGATATCCAGCACAGTGG
24	OHtest12rv1	TCGAGCAAGTAGTTAGGGTTAAC
25	OHtest12fw2	**AGATTGAGAA**TATTCTGCAGATATCCAGCACAGTGG
26	OHtest12rv2	**TTCTCAATCT** TCGAGCAAGTAGTTAGGGTTAAC
27	OHtest14fw1	TATTCTGCAGATATCCAGCACAGTGG
28	OHtest14rv1	GAGCAAGTAGTTAGGGTTAACTAC
29	OHtest14fw2	**GAAGATTGAGAA**TATTCTGCAGATATCCAGCACAGTGG
30	OHtest14rv2	**TTCTCAATCTTC**GAGCAAGTAGTTAGGGTTAACTAC
31	OHtest16fw1	TATTCTGCAGATATCCAGCACAGTGG
32	OHtest16rv1	GCAAGTAGTTAGGGTTAACTACC
33	OHtest16fw2	**TCGAAGATTGAGAA**TATTCTGCAGATATCCAGCACAGTGG
34	OHtest16rv2	**TTCTCAATCTTCGA**GCAAGTAGTTAGGGTTAACTACC

35	OHtest18fw1	TATTCTGCAGATATCCAGCACAGTGG
36	OHtest18rv1	AAGTAGTTAGGGTTAACTACCAAGTAG
37	OHtest18fw2	**GCTCGAAGATTGAGAA**TATTCTGCAGATATCCAGCACAGTGG
38	OHtest18rv2	**TTCTCAATCTTCGAGC**AAGTAGTTAGGGTTAACTACCAAGTAG
39	OHtest20fw1	TATTCTGCAGATATCCAGCACAGTGG
40	OHtest20rv1	GTAGTTAGGGTTAACTACCAAGTAGGG
41	OHtest20fw2	**TTGCTCGAAGATTGAGAA**TATTCTGCAGATATCCAGCACAGTGG
42	OHtest20rv2	**TTCTCAATCTTCGAGCAA**GTAGTTAGGGTTAACTACCAAGTAGGG
		**Point mutations**
43	YAAU-R205Afw1	GCAGTAAAAGAGTGCGAAGAGATGATG
44	YAAU-R205Arv1	GCGTAATAACCAGCGGGGAGATTC
45	YAAU-R205Afw2	**AAAGGG**GCAGTAAAAGAGTGCGAAGAGATG
46	YAAU-R205Arv2	**CCCTTT**GCGTAATAACCAGCGGGGAGATTC
47	ILES-K581Afw1	GCAGGTAAAGCGCCGTATCGTCAGGTAC
48	ILES-K581Arv1	GGTGGAGATCATTAGGGAAGACATG
49	ILES-K581Afw2	**GCGATG**GCAGGTAAAGCGCCGTATCGTCAGGTAC
50	ILES-K581Arv2	**CATCGC**GGTGGAGATCATTAGGGAAGACATG
51	TALB-K193Cfw1	TGCGAGTACGCTCCGGCAGAAGATCC
52	TALB-K193Crv1	ATCGGTATTCGCTTTGTACCAGTC
53	TALB-K193Cfw2	**AAGAAA**TGCGAGTACGCTCCGGCAGAAGATCC
54	TALB-K193Crv2	**TTTCTT**ATCGGTATTCGCTTTGTACCAGTC
55	APAG-R26Afw1	GCTTACGTTTTTGCTTATACCGTAAC
56	APAG-R26Arv1	TTATCAGGTGAAGATTGAGCCTCAATG
57	APAG-R26Afw2	**TGAACG**GCTTACGTTTTTGCTTATACCGTAAC
58	APAG-R26Arv2	**CGTTCA**TTATCAGGTGAAGATTGAGCCTCAATG
59	GAST-K75Afw1	GCGCAGGGACCATGGCTGGAGGAAG
60	GAST-K75Arv1	GACGGGTCTGCCACGAGGTGTGG
61	GAST-K75Afw2	**CAAGAA**GCGCAGGGACCATGGCTGGAGGAAG
62	GAST-K75Arv2	**TTCTTG**GACGGGTCTGCCACGAGGTGTGG
63	MCM6-Q641Afw1	GCACCTAAACATGTGAAGGAAGCTTTC
64	MCM6-Q641Arv1	ATCACAGCAGTGCATCCGAGCCATAG
65	MCM6-Q641Afw2	**GAGGTC**GCGCAGGGACCATGGCTGGAGGAAG
66	MCM6-Q641Arv2	**GACCTC**ATCACAGCAGTGCATCCGAGCCATAG
67	PRRT2-R125Afw1	GCGCTGGAGTCTGCAGCCCCACCTG
68	PRRT2-R125Arv1	CTGGTCTGCAGTGGCCTCTTTGCTC
69	PRRT2-R125Afw2	**GGGTCC**GCGCTGGAGTCTGCAGCCCCACCTG
70	PRRT2-R125Arv2	**GGACCC**CTGGTCTGCAGTGGCCTCTTTGCTC
71	SLC18A2-K354Afw1	GCAATGGGGAGGTGGCTTTGTGCTCTTC
72	SLC18A2-K354Arv1	TGCAAGTATCCCAAAAATATTGG
73	SLC18A2-K354Afw2	**CACAAA**GCAATGGGGAGGTGGCTTTGTGCTCTTC

74	SLC18A2-K354Arv2	**TTTGTG**TGCAAGTATCCCAAAAATATTGG
75	YAAU-R205Afw	GTTATTACGCAAAGGGGCAGTAAAAGAGTGCGAAG
76	YAAU-R205Arv	CTTCGCACTCTTTTACTGCCCCTTTGCGTAATAAC
77	ILES-K581Afw	GATCTCCACCGCGATGGCAGGTAAAGCGCCGTATC
78	ILES-K581Arv	GATACGGCGCTTTACCTGCCATCGCGGTGGAGATC
79	TALB-K193Cfw	GCGAATACCGATAAGTGCGAGTACGCTCCGGC
80	TALB-K193Crv	GCCGGAGCGTACTCGCACTTATCGGTATTCGC
81	APAG-R26Afw	CACCTGATAATGAAGCTTACGTTTTTGCTTATAC
82	APAG-R26Arv	GTATAAGCAAAAACGTAAGCTTCATTATCAGGTG
83	GAST-K75Afw	GACCCGTCCAAGGCGCAGGGACCATG
84	GAST-K75Arv	CATGGTCCCTGCGCCTTGGACGGGTC
85	MCM6-Q641Afw	CTGCTGTGATGAGGTCGCACCTAAACATGTGAAG
86	MCM6-Q641Arv	CTTCACATGTTTAGGTGCGACCTCATCACAGCAG
87	PRRT2-R125Afw	GACCAGGGGTCCGCGCTGGAGTCTG
88	PRRT2-R125Arv	CAGACTCCAGCGCGGACCCCTGGTC
89	SLC18A2-K354Afw	GATACTTGCACACGCAATGGGGAGGTGGC
90	SLC18A2-K354Arv	GCCACCTCCCCATTGCGTGTGCAAGTATC
91	YAAU-DE224AAfw1	GCCGCAGAGCAGCCGCAGCAAACCCGTTTTC
92	YAAU-DE224AArv1	CACCGGTTCGCCAAACAGTTTGATC
93	YAAU-DE224AAfw2	**GCTTTC**GCCGCAGAGCAGCCGCAGCAAACCCG
94	YAAU-DE224AArv2	**GAAAGC**CACCGGTTCGCCAAACAGTTTGATC
95	GAST-SQQ27AAAfw1	GCCGCAGCGCCAGATGCACCCTTAGGTACAGG
96	GAST-QQQ27AAArv1	CACTCGGGGCGAATTGATCATATG
97	GAST-SQQ27AAAfw2	**CCCCGC**GCCGCAGCGCCAGATGCACCCTTAGGTACAGG
98	GAST-SQQ27AAArv2	**GCGGGG**CTTCCAAGAAGCTTCAGAGAAGG
99	YAAU-RKGRVKECEE202AAAAAAAAAAfw1	GCCGCAGCGGCCGCAGCGGCCGCAGCGGCAATGATGATCAAACTGTTTGGCGAAC
100	YAAU-RKGRVKECEE202AAAAAAAAAArv1	CAGCGGGGAGATTCAGGCAGTTCG
101	YAAU-RKGRVKECEE202AAAAAAAAAAfw2	**GTTATTA**GCCGCAGCGGCCGCAGCGGCCGCAG
102	YAAU-RKGRVKECEE202AAAAAAAAAArv2	**TAATAAC**CAGCGGGGAGATTCAGGCAGTTCG
103	GAST-EQQGPASHHRRQ48AAAAAAAAAAAAfw1	GCCGCAGCGGCCGCAGCGGCCGCAGCGGCAGCAGCGCTGGGACCCCAGGGTCCCCCACAC
104	GAST-EQQGPASHHRRQ48AAAAAAAAAAAArv1	TTCGCCCTGGACTTCGGTTTCACG
105	GAST-EQQGPASHHRRQ48AAAAAAAAAAAAfw2	**CTGGCTG**GCCGCAGCGGCCGCAGCGGCCGCAG

106	GAST-EQQGPASHHRRQ48AAAAAAAAAAArv2	**CAGCCAG**GGTAGCTCCAGGTCCCTGTTGG
		**Deletions**
107	YAAU-DEL-909Afw1	AACACTGCCGGACGGCGTCCATTG
108	YAAU-DEL-909Arv1	TTCGCCCTGGACTTCGGTTTCACG
109	YAAU-DEL-909Afw2	**GTGGTT**AACACTGCCGGACGGCGTCCATTG
110	YAAU-DEL-909Arv2	**AACCAC**TTCGCCCTGGACTTCGGTTTCACG
111	ILES-DEL-2096Tfw1	CCTCAAGGCGTACGAAGCATACG
112	ILES-DEL-2096Trv1	CTGTGCCGCTTTCGCACAACCTAC
113	ILES-DEL-2096Tfw2	**GAAGACA**CCTCAAGGCGTACGAAGCATACG
114	ILES-DEL-2096Trv2	**TGTCTTC**CTGTGCCGCTTTCGCACAACCTAC
115	TALB-DEL-552Cfw1	TGGTACAAAGCGAATACCGATAAG
116	TALB-DEL-552Crv1	ATACGGCCAACAAACGGCGAGATC
117	TALB-DEL-552Cfw2	**TCTTGA**TGGTACAAAGCGAATACCGATAAG
118	TALB-DEL-552Crv2	**TCAAGA**ATACGGCCAACAAACGGCGAGATC
119	APAG-DEL-253Gfw1	GTGCAATCATTGAAACCCCGCTG
120	APAG-DEL-253Grv1	GTACTGGTACTCTTCGCCAGGC
121	APAG-DEL-253Gfw2	**ACCAGC**GTGCAATCATTGAAACCCCGCTG
122	APAG-DEL-253Grv2	**GCTGGT**GTACTGGTACTCTTCGCCAGGC
123	GAST-DEL-183Afw1	CCCCAGGGTCCCCCACACCTCG
124	GAST-DEL-183Arv1	TGCCTTCGATGATGAGAGGCTG
125	GAST-DEL-183Afw2	**GCTGGG**CCCCAGGGTCCCCCACACCTCG
126	GAST-DEL-183Arv2	**CCCAGC**TGCCTTCGATGATGAGAGGCTG
127	MCM6-DEL-1745Tfw1	TAAACCCAAGATTTCCAAAGAGTC
128	MCM6-DEL-1745Trv1	TTGCAAAGAGAAGATATCTTCTG
129	MCM6-DEL-1745Tfw2	**GACAGT**TAAACCCAAGATTTCCAAAGAGTC
130	MCM6-DEL-1745Trv2	**ACAGTC**TTGCAAAGAGAAGATATCTTCTG
131	PRRT2-DEL-1741Cfw1	AGCTCCCAGCTGGCAGGTCCTGG
132	PRRT2-DEL-1741Crv1	CGGCTCAGGCTACCTCGGGGAGATC
133	PRRT2-DEL-1741Cfw2	**CCACCC**AGCTCCCAGCTGGCAGGTCCTGG
134	PRRT2-DEL-1741Crv2	**GGGTGG**CGGCTCAGGCTACCTCGGGGAGATC
135	SLC18A2-DEL-1415Gfw1	TCCACCTGCCAAAGAAGAAAAAATG
136	SLC18A2-DEL-1415Grv1	AAAAAAGCAGAGAGGGGCAAAAAGAATATC
137	SLC18A2-DEL-1415Gfw2	**CTTCGAA**TCCACCTGCCAAAGAAGAAAAAATG
138	SLC18A2-DEL-1415Grv2	**TTCGAAG**AAAAAAGCAGAGAGGGGCAAAAAGAATATC
139	YAAU DEL-F28-Gly31fw1	TATGTACTGGTAATGATTGGCGTG
140	YAAU DEL-F28-Gly31rv1	ACCGCTTCCCCACAGCAAAATGC

141	YAAU DEL-F28-Gly31fw2	**GGTCCG**TATGTACTGGTAATGATTGGCGTG
142	YAAU DEL-F28-Gly31rv2	**CGGACC**ACCGCTTCCCCACAGCAAAATGC
143	ILES DEL-E201-D204fw1	AAAACTTCTCCGTCCATCGACGTTG
144	ILES DEL-E201-D204rv1	CGCTTCCGCCAGCGCAGAACGGC
145	ILES DEL-E201-D204fw2	**GAAGTT**AAAACTTCTCCGTCCATCGACGTTG
146	ILES DEL-E201-D204rv2	**AACAAC**CGCTTCCGCCAGCGCAGAACGGC
147	TALB DEL-Q28-D31fw1	GCCACAACCAACCCTTCTCTCATTC
148	TALB DEL-Q28-D31rv1	CTTCATTGCCGCGATGTCCCCAG
149	TALB DEL-Q28-D31fw2	**CTGTAT**GCCACAACCAACCCTTCTCTCATTC
150	TALB DEL-Q28-D31rv2	**ATACAG**CTTCATTGCCGCGATGTCCCCAG
151	APAG DEL-Q63-G66fw1	GTGGTTGGCGTCCAGCCACTTATC
152	APAG DEL-Q63-G66rv1	GGTTTCACGGCCATTGCCATTG
153	APAG DEL-Q63-G66fw2	**GAAGTC**GTGGTTGGCGTCCAGCCACTTATC
154	APAG DEL-Q63-G66rv2	**GACTTC**GGTTTCACGGCCATTGCCATTG
155	GAST DEL-H55-R58fw1	CAGCTGGGACCCCAGGGTCCCCCAC
156	GAST DEL-H55-R58rv1	TGGGCCCTGCTGCTCCAGCCAGG
157	GAST DEL-H55-R58fw2	**GCCTCT**CAGCTGGGACCCCAGGGTCCCCCAC
158	GAST DEL-H55-R58rv2	**AGAGGC**TGGGCCCTGCTGCTCCAGCCAGG
159	MCM6 DEL-D202-K205fw1	GTTCGTATTCAAGAGACCCAAG
160	MCM6 DEL-D202-K205rv1	TTGATTTATTTGTATCCAGTAAG
161	MCM6 DEL-D202-K205fw2	**GATTTGTT**GTTCGTATTCAAGAGACCCAAG
162	MCM6 DEL-D202-K205rv2	**AACAAATC**TTGATTTATTTGTATCCAGTAAG
163	PRRT2 DEL-D43-E45fw1	GCCCCGCAGCCAGGTCCAAACAC
164	PRRT2 DEL-D43-E45rv1	CCCTGCTAGGACCTGGGGAGGGC
165	PRRT2 DEL-D43-E45fw2	**GTACCA**GCCCCGCAGCCAGGTCCAAACAC
166	PRRT2 DEL-D43-E45rv2	**TGGTAC**CCCTGCTAGGACCTGGGGAGGGC
167	SLC18A2 DEL-D73-Q76fw1	AGCATCTTCTCCTATTATGATAAC
168	SLC18A2 DEL-D73-Q76rv1	GAGGCAGTGTGCACTGGCCTGGC

169	SLC18A2 DEL-D73-Q76fw2	**CATCTCA**AGCATCTTCTCCTATTATGATAAC
170	SLC18A2 DEL-D73-Q76rv2	**TGAGATG**GAGGCAGTGTGCACTGGCCTGGC
171	YAAU DEL-K11-N434fw1	ATGACGATCCGCGGGCAGAGAATG
172	YAAU DEL-K11-N434rv1	GTCAAAGTTTCTGGACGGTTGC
173	YAAU DEL-K11-N434fw2	**GATCTCA**ATGACGATCCGCGGGCAGAGAATG
174	YAAU DEL-K11-N434rv2	**TGAGATC**GTCAAAGTTTCTGGACGGTTGC
175	ILES DEL-G14-A929fw1	GGTGACGGTGAAAAACGTAAGTTTG
176	ILES DEL-G14-A929rv1	CGGCAAATTCAGGGTTGATTTATAG
177	ILES DEL-G14-A929fw2	**GAAACA**ATGACGATCCGCGGGCAGAGAATG
178	ILES DEL-G14-A929rv2	**TGTTTC**CGGCAAATTCAGGGTTGATTTATAG
179	TALB DEL-V14-K308fw1	CTGGAAAAAATGATCGGCGATCTG
180	TALB DEL-V14-K308rv1	TACTGACGAAGGGAGGTCAATTTG
181	TALB DEL-V14-K308fw2	**CACCACC**CTGGAAAAAATGATCGGCGATCTG
182	TALB DEL-V14-K308rv2	**GGTGGTG**TACTGACGAAGGGAGGTCAATTTG
183	APAG DEL-V14-F116fw1	CGACTCGCCGTTCCCACACTCATTC
184	APAG DEL-V14-F116rv1	AACCTGAATACACACTCGGGGCG
185	APAG DEL-V14-F116fw2	**CAAAGC**CGACTCGCCGTTCCCACACTCATTC
186	APAG DEL-V14-F116rv2	**GCTTTG**AACCTGAATACACACTCGGGGCG
187	GAST DEL-V14-F116fw1	GGCCGCCGCAGTGCTGAGGATGAG
188	GAST DEL-V14-F116rv1	AAAGATCAGCACATACACACATAG
189	GAST DEL-V14-F116fw2	**GCACTG**GGCCGCCGCAGTGCTGAGGATGAG
190	GAST DEL-V14-F116rv2	**CAGTGC**AAAGATCAGCACATACACACATAG
191	MCM6 DEL-Q14-V812fw1	GTTAACCCTAACTACTTGCTCGAAG
192	MCM6 DEL-Q14-V812rv1	GGCGCCCGGCTCCGCTGCCGCCGC
193	MCM6 DEL-Q14-V812fw2	**GGCAGC**GTTAACCCTAACTACTTGCTCGAAG
194	MCM6 DEL-Q14-V812rv2	**GCTGCC**GGCGCCCGGCTCCGCTGCCGCCGC
195	PRRT2 DEL-V14-S331fw1	TGCGTCATCAACTTAGGCGTGTATAAG
196	PRRT2 DEL-V14-S331rv1	ATCTCAGAGATCTCAGAGCTGCTG

197	PRRT2 DEL-V14-S331fw2	**GAAGGGG**TGCGTCATCAACTTAGGCGTGTATAAG
198	PRRT2 DEL-V14-S331rv2	**CCCCTTC**ATCTCAGAGATCTCAGAGCTGCTG
199	SLC18A2 DEL-E14-I505fw1	GGTGAAGATGAAGAATCTGAAAGTG
200	SLC18A2 DEL-E14-I505rv1	CCAGCGGACCAGCGCCAGCTCGC
201	SLC18A2 DEL-E14-I505fw2	**CTGCAG**GGTGAAGATGAAGAATCTGAAAGTG
202	SLC18A2 DEL-E14-I505rv2	**CTGCAG**CCAGCGGACCAGCGCCAGCTCGC
		**Insertions**
203	YAAU Ins-A-909fw1	AAACACTGCCGGACGGCGTCCATTG
204	YAAU Ins-A-909rv1	AGCATCGGCGGAATACAGCCGAG
205	YAAU Ins-A-909fw2	**GTGGTTA**AAACACTGCCGGACGGCGTCCATTG
206	YAAU Ins-A-909rv2	**TAACCAC**AGCATCGGCGGAATACAGCCGAG
207	ILES Ins-T-2096fw1	TCCTCAAGGCGTACGAAGCATACG
208	ILES Ins-T-2096rv1	CTGTGCCGCTTTCGCACAACCTAC
209	ILES Ins-T-2096fw2	**GAAGACAT**TCCTCAAGGCGTACGAAGCATACG
210	ILES Ins-T-2096rv2	**ATGTCTTC**CTGTGCCGCTTTCGCACAACCTAC
211	TALB Ins-C-552fw1	CCTGGTACAAAGCGAATACCGATAAG
212	TALB Ins-C-552rv1	GGCCAACAAACGGCGAGATCAGG
213	TALB Ins-C-552fw2	**GTATTCTTGA**CCTGGTACAAAGCGAATACCGATAAG
214	TALB Ins-C-552rv2	**TCAAGAATAC**GGCCAACAAACGGCGAGATCAGG
215	APAG Ins-G-253fw1	GGTGCAATCATTGAAACCCCGCTG
216	APAG Ins-G-253rv1	TGTACTGGTACTCTTCGCCAGGC
217	APAG Ins-G-253fw2	**CCAGCG**GGTGCAATCATTGAAACCCCGCTG
218	APAG Ins-G-253rv2	**CGCTGG**TGTACTGGTACTCTTCGCCAGGC
219	GAST Ins-A-183fw1	ACCCCAGGGTCCCCCACACCTCG
220	GAST Ins-A-183rv1	CTGCCTTCGATGATGAGAGGCTG
221	GAST Ins-A-183fw2	**CTGGGA**ACCCCAGGGTCCCCCACACCTCG
222	GAST Ins-A-183rv2	**TCCCAG**CTGCCTTCGATGATGAGAGGCTG
223	MCM6 Ins-T-1745fw1	TTAAACCCAAGATTTCCAAAGAGTC
224	MCM6 Ins-T-1745rv1	TTGCAAAGAGAAGATATCTTCTG
225	MCM6 Ins-T-1745fw2	**GACAGTT**TTAAACCCAAGATTTCCAAAGAGTC
226	MCM6 Ins-T-1745rv2	**AACTGTC**TTGCAAAGAGAAGATATCTTCTG
227	PRRT2 Ins-C-741fw1	CAGCTCCCAGCTGGCAGGTCCTG
228	PRRT2 Ins-C-741rv1	GCGGCTCAGGCTACCTCGGGGAG
229	PRRT2 Ins-C-741fw2	**CACCCC**CAGCTCCCAGCTGGCAGGTCCTG
230	PRRT2 Ins-C-741rv2	**GGGGTG**GCGGCTCAGGCTACCTCGGGGAG
231	SLC18A2 Ins-G-1415fw1	GTCCACCTGCCAAAGAAGAAAAAATG

232	SLC18A2 Ins-G-1415rv1	AAAAAAGCAGAGAGGGGCAAAAAG
233	SLC18A2 Ins-G-1415fw2	**CTTCGAAG**GTCCACCTGCCAAAGAAGAAAAAATG
234	SLC18A2 Ins-G-1415rv2	**CTTCGAAG**AAAAAAGCAGAGAGGGGCAAAAAG
235	YAAU Ins-F28-AAAAfw1	GCCGCAGCGGCCCTGGATGGTTATGTACTGGTAATG
236	YAAU Ins-F28-AAAArv1	ACCACCGCTTCCCCACAGCAAAATG
237	YAAU Ins-F28-AAAAfw2	**CCGTTT**GCCGCAGCGGCCCTGGATGGTTATG
238	YAAU Ins-F28-AAAArv2	**AAACGG**ACCACCGCTTCCCCACAGCAAAATG
239	ILES Ins-E201-AAAAfw1	GCCGCAGCGGCCTATTACGACAAAACTTCTCCGTCC
240	ILES Ins-E201-AAAArv1	TTCCGCTTCCGCCAGCGCAGAACG
241	ILES Ins-E201-AAAAfw2	**GTTGAG**GCCGCAGCGGCCTATTACGACAAAAC
242	ILES Ins-E201-AAAArv2	**CTCAAC**TTCCGCTTCCGCCAGCGCAGAACG
243	TALB Ins-Q28-AAAAfw1	GCCGCAGCGGCCCCGCAGGATGCCACAACCAACCC
244	TALB Ins-Q28-AAAArv1	AGCTTCATTGCCGCGATGTCCCC
245	TALB Ins-Q28-AAAAfw2	**GTATCAAG**CCGCAGCGGCCCCGCAGGATGCC
246	TALB Ins-Q28-AAAArv2	**CTTGATAC**AGCTTCATTGCCGCGATGTCCCC
247	APAG Ins-Q63-AAAAfw1	GCCGCAGCGGCCGGCGAAGGAGTGGTTGGCGTCCAG
248	APAG Ins-Q63-AAAArv1	TTCGGTTTCACGGCCATTGCCATTG
249	APAG Ins-Q63-AAAAfw2	**GTCCAG**CCGCAGCGGCCGGCGAAGGAGTG
250	APAG Ins-Q63-AAAArv2	**CTGGAC**TTCGGTTTCACGGCCATTGCCATTG
251	GAST Ins-H55-AAAAfw1	GCCGCAGCGGCCCATCGAAGGCAGCTGGGACCCCAG
252	GAST Ins-H55-AAAArv1	AGGCTGGGCCCTGCTGCTCCAGC
253	GAST Ins-H55-AAAAfw2	**CTCATC**GCCGCAGCGGCCCATCGAAGGCAGC
254	GAST Ins-H55-AAAArv2	**GATGAG**AGGCTGGGCCCTGCTGCTCCAGC
255	MCM6 Ins-H55-AAAAfw1	GCCGCAGCGGCCTTTCAAAAGGTTCGTATTCAAGAG
256	MCM6 Ins-H55-AAAArv1	AAATCTTGATTTATTTGTATCCAG
257	MCM6 Ins-H55-AAAAfw2	**GTTGAT**GCCGCAGCGGCCTTTCAAAAGGTTC
258	MCM6 Ins-H55-AAAArv2	**CATCAAC**AAATCTTGATTTATTTGTATCCAG
259	PRRT2 Ins-D43-AAAAfw1	GCCGCAGCGGCCCAGCCAGAGGCCCCGCAGCCAGG

260	PRRT2 Ins-D43-AAAArv1	TACCCCTGCTAGGACCTGGGGAG
261	PRRT2 Ins-D43-AAAAfw2	**CCAGAC**GCCGCAGCGGCCCAGCCAGAGGC
262	PRRT2 Ins-D43-AAAArv2	**GTCTGG**TACCCCTGCTAGGACCTGGGGAG
263	SLC18A2 Ins-D73-AAAAfw1	GCCGCAGCGGCCAGCTTCCAGAGCATCTTCTCCTATTATG
264	SLC18A2 Ins-D73-AAAArv1	ATGGAGGCAGTGTGCACTGGCC
265	SLC18A2 Ins-D73-AAAAfw2	**CTCAGAC**GCCGCAGCGGCCAGCTTCCAGAGC
266	SLC18A2 Ins-D73-AAAArv2	**GTCTGAG**ATGGAGGCAGTGTGCACTGGCC
267	YAAU Ins-F28-60BPfw1	GTTGAGGAGAGTCCCAAGGTTCCAGGCGAAGGGCCTGGCCATTCTGAAGCTGAAACTGGC CTGGATGGTTATGTACTGGTAATG
268	YAAU Ins-F28-60BPrv1	ACCACCGCTTCCCCACAGCAAAATG
269	YAAU Ins-F28-60BPfw2	**CCGTTTG**GTTGAGGAGAGTCCCAAGGTTCCAG
270	YAAU Ins-F28-60BPrv2	**CAAACGG**ACCACCGCTTCCCCACAGCAAAATG
271	SLC18A2 Ins-D73-60BPfw1	GTTGAGGAGAGTCCCAAGGTTCCAGGCGAAGGGCCTGGCCATTCTGAAGCTGAAACTGGC AGCTTCCAGAGCATCTTCTCCTATTATG
272	SLC18A2 Ins-D73-60BPrv1	ATGGAGGCAGTGTGCACTGGCC
273	SLC18A2 Ins-D73-60BPfw2	**CTCAGAC**GTTGAGGAGAGTCCCAAGGTTCCAG
274	SLC18A2 Ins-D73-60BPrv2	**GTCTGAG**ATGGAGGCAGTGTGCACTGGCC
		**Multiple-site mutagenesis**
275	MCM6-E52Afw1	CACTGATTCGTCCTGAGAGAAAC
276	MCM6-E52Arv1	TAATTGCAAGTATTTAATTTCTCC
277	MCM6-E52Afw2	**GCAGAGG**CACTGATTCGTCCTGAGAGAAAC
278	MCM6-E52Arv2	**CCTCTGC**TAATTGCAAGTATTTAATTTCTCC
279	MCM6-E103Afw1	CGATCCCTCTTGCCAAGGATTTTTATG
280	MCM6-E103Arv1	GTCTTTGACGAATGTTTTCAAGG
281	MCM6-E103Afw2	**CGTAAAG**CGATCCCTCTTGCCAAGGATTTTTATG
282	MCM6-E103Arv2	**CTTTACG**GTCTTTGACGAATGTTTTCAAGG
283	MCM6-D160Afw1	CCTGTCAGACAGTGATCAGGGATG
284	MCM6-D160Arv1	AGAAAAGTTCCGCTCACAAGCTC
285	MCM6-D160Afw2	**GTGCTTGG**CCTGTCAGACAGTGATCAGGGATG
286	MCM6-D160Arv2	**CCAAGCAC**AGAAAAGTTCCGCTCACAAGCTC
287	MCM6-R207Afw1	GCCATTCAAGAGACCCAAGCTGAG
288	MCM6-R207Arv1	TAATTGCAAGTATTTAATTTCTCC
289	MCM6-R207Afw2	**CAAAAGGTT**CACTGATTCGTCCTGAGAGAAAC
290	MCM6-R207Arv2	**AACCTTTTG**TAATTGCAAGTATTTAATTTCTCC
291	MCM6-D253Afw1	CCGTCTCCAAGCTTAGCACACC

292	MCM6-D253Arv1	AACAATCAGTGTCCCTGTAAAG
293	MCM6-D253Afw2	**GTGCCTG**CCGTCTCCAAGCTTAGCACACC
294	MCM6-D253Arv2	**CAGGCAC**AACAATCAGTGTCCCTGTAAAG
295	MCM6-R309Afw1	GCATTTGGGGGGAAAGAGCTCAGAG
296	MCM6-R309Arv1	TTGGCGCAACACAGCAGGCAAGAAAG
297	MCM6-R309Afw2	**CCAACCCA**GCATTTGGGGGGAAAGAGCTCAGAG
298	MCM6-R309Arv2	**TGGGTTGG**TTGGCGCAACACAGCAGGCAAGAAAG
299	MCM6-D362Afw1	CTGAAGTAAAACGGGGTGTCC
300	MCM6-D362Arv1	ATGTATAGTAGGGAACAGGCTGG
301	MCM6-D362Afw2	**GGCAATG**CTGAAGTAAAACGGGGTGTCC
302	MCM6-D362Arv2	**CATTGCC**ATGTATAGTAGGGAACAGGCTGG
303	MCM6-R415Afw1	GCAGCTGTCTACACCAGTGGTAAAG
304	MCM6-R415Arv1	AACTCCTCCACGTGCTTGAGAAATTGG
305	MCM6-R415Afw2	**CAGCCCC**GCAGCTGTCTACACCAGTGGTAAAG
306	MCM6-R415Arv2	**GGGGCTG**AACTCCTCCACGTGCTTGAGAAATTGG
307	MCM6-E461Afw1	CCTTTGATAAGATGGACGTGC
308	MCM6-E461Arv1	AACACACACCATTATCAGCCAAC
309	MCM6-E461Afw2	**GTATTGATG**CACTGATTCGTCCTGAGAGAAAC
310	MCM6-E461Arv2	**CATCAATAC**AACACACACCATTATCAGCCAAC
311	MCM6-D511Afw1	CCAGATCAAAATCATTGAAACAG
312	MCM6-D511Arv1	TCCACTGATTGGGTTTGCTGC
313	MCM6-D511Afw2	**CACTATG**CCAGATCAAAATCATTGAAACAG
314	MCM6-D511Arv2	**CATAGTG**TCCACTGATTGGGTTTGCTGC
315	MCM6-D564Afw1	CTCGTGTCTATTCCCTCGATG
316	MCM6-D564Arv1	ATTCCTCAATTCTTGAATGC
317	MCM6-D564Afw2	**CAATTG**CTCGTGTCTATTCCCTCGATG
318	MCM6-D564Arv2	**CAATTG**ATTCCTCAATTCTTGAATGC
319	MCM6-R619Afw1	GCACAGCTTGAGAGCATGATTCG
320	MCM6-R619Arv1	CTCCATGAAGACTTGGTCACTCCAG
321	MCM6-R619Afw2	**GATTACAGTG**GCACAGCTTGAGAGCATGATTCG
322	MCM6-R619Arv2	**CACTGTAATC**CTCCATGAAGACTTGGTCACTCCAG
323	MCM6-Q668Afw1	GCAGAGGAAGAGATCCAGATGGAG
324	MCM6-Q668Arv1	ACATCAGGTGTTTCCACACG
325	MCM6-Q668Afw2	**CAATCTAGAT**GCAGAGGAAGAGATCCAGATGGAG
326	MCM6-Q668Arv2	**ATCTAGATTG**ACATCAGGTGTTTCCACACG
327	MCM6-E719Afw1	CGTACTGCCGAATCTCTAACC
328	MCM6-E719Arv1	CCCAGCCTTAAGGAGGCTTTGG
329	MCM6-E719Afw2	**CTTCTCTG**CACTGATTCGTCCTGAGAGAAAC
330	MCM6-E719Arv2	**CAGAGAAG**CCCAGCCTTAAGGAGGCTTTGG

331	MCM6-D784Afw1	CTCATGTTCTAATTGAGCTCACC
332	MCM6-D784Arv1	TGTGAGTCGATGAATAACTTTC
333	MCM6-D784Afw2	**CACTATG**CTCATGTTCTAATTGAGCTCACC
334	MCM6-D784Arv2	**CATAGTG**TGTGAGTCGATGAATAACTTTC

* Bold purple letters show overhangs of forward primers, and bold green letters show overhangs of reverse primers. Underlined letters represent the nucleotides mutated.

Supplementary Table S2. PCR reaction components.

	Reaction 1*	Reaction 2a#	Reaction 2b#
Template DNA	~50 ng	~500 ng	~500 ng
Forward primer (100 µM)	0.25 µL	0.25 µL	
Reverse primer (100 µM)	0.25 µL		0.25 µL
Phusion GC Buffer (5×)	10 µL	10 µL	10 µL
dNTPs (10 mM)	1 µL	1 µL	1 µL
DMSO (100%)	1.5 µL	1.5 µL	1.5 µL
Phusion High Fidelity DNA Polymerase	1 µL	1 µL	1 µL
add water to	50 µL	50 µL	50 µL

*PCR with forward and reverse primer; #PCR with forward or reverse primer

Supplementary Table S3. PCR thermocycling conditions.

Step	Temperature	Time
Initial Denaturation	98°C	5 min
20 Cycles	98°C	20 seconds
	60-50 °C, step -0.5 °C	20 seconds
	72°C	1 minute/kbp
10 Cycles	98°C	20 seconds
	52 °C	20 seconds
	72°C	1 minute/kbp
Final Extension	72°C	10 minutes
Hold	4 °C	

Supplementary Table S4 PCR product reannealing conditions

Steps	Temperature (°C)	Time (min)
1	95	5
2	90	1
3	80	1
4	70	0.5
5	60	0.5
6	50	0.5
7	40	0.5
8	37	Holding

Supplementary Table S5. The efficiency and fidelity of point mutations with QuikChange™ method.

Gene	Gene ID	Length (bp)	Vector	Mutation	CFU/µg DNA[a]	Positive (%)[b]
yaaU	944766	1,332	pNGFP-BC	R205A	7305 ± 735	85.3 ± 7.5
ileS	944761	2,817	pCGFP-BC	K581A	8006 ± 856	85.4 ± 10.5
talB	944748	954	pNGFP-BC	K193C	7507 ± 858	90.5 ± 9.5
apaG	944772	378	pCGFP-BC	R26A	8008 ± 821	85.6 ± 7.5
GAST	2520	306	pNGFP-EU	K75A	7105 ± 653	90.5 ± 10.4
MCM6	4175	2,466	pNGFP-EU	Q641A	8703 ± 714	80.4 ± 6.8
SLC18A2	6571	1,545	pCGFP-EU	K354A	7007 ± 835	85.6 ± 8.6

[a] Reported results are the mean ± s.d. of three independent experiments.
[b] For each independent experiment, ten of colonies were checked by DNA sequencing.

Supplementary Table S6. 20 kb genomic DNA fragment from *E.coli*.

Genes	JW ID	Direction	Left nt	Right nt
hlpA	JW0173	+	200485	200946
lpxD	JW0174	+	200974	201975
fabZ	JW0175	+	202104	202535
lpxA	JW0176	+	202563	203327
lpxB	JW0177	+	203351	204475
rnhB	JW0178	+	204496	205068
dnaE	JW0179	+	205129	208587
accA	JW0180	+	208624	209559
ldcC	JW0181	+	209682	211799
yaeR	JW0182	+	211880	212245
tilS	JW0183	+	212334	213608
rof	JW0184	-	213699	213929
yaeP	JW0185	-	213946	214122
yaeQ	JW0186	+	214294	214815
yaeJ	JW0187	+	214836	215234
nlpE	JW0188	+	215272	215958
yaeF	JW5016	-	216200	217000
proS	JW0190	-	217078	218772
yaeB	JW0191	-	218908	219591
rcsF	JW0192	-	219612	219992
metQ	JW0193	-	220134	220925

Supplementary Table S7. 45 kb DNA fragment from *E.coli* genome.

Gene	JW ID	Direction	Left nt	Right nt
hlpA	JW0173	+	200485	200946
lpxD	JW0174	+	200974	201975
fabZ	JW0175	+	202104	202535
lpxA	JW0176	+	202563	203327
lpxB	JW0177	+	203351	204475
rnhB	JW0178	+	204496	205068
dnaE	JW0179	+	205129	208587
accA	JW0180	+	208624	209559
ldcC	JW0181	+	209682	211799
yaeR	JW0182	+	211880	212245
tilS	JW0183	+	212334	213608
rof	JW0184	-	213699	213929
yaeP	JW0185	-	213946	214122
yaeQ	JW0186	+	214294	214815
yaeJ	JW0187	+	214836	215234
nlpE	JW0188	+	215272	215958
yaeF	JW5016	-	216200	217000
proS	JW0190	-	217078	218772
yaeB	JW0191	-	218908	219591
rcsF	JW0192	-	219612	219992
metQ	JW0193	-	220134	220925
metI	JW0194	-	220989	221618
metN	JW0195	-	221635	222642
gmhB	JW0196	+	222836	223387
dkgB	JW0197	+	229170	229949
yafC	JW0198	-	229988	230878
yafD	JW5017	+	231125	231901
yafE	JW0200	+	231929	232528
mltD	JW5018	-	232618	233952
gloB	JW0202	-	234048	234779
yafS	JW0203	+	234819	235517
rnhA	JW0204	-	235556	235999
dnaQ	JW0205	+	236070	236777
yafT	JW0206	+	237338	238099
yafU	JW0207	-	238767	239081

yafF	JW0208	+	239193	239357
yafV	JW5019	-	239440	240186
ivy	JW0210	+	240346	240795
fadE	JW5020	-	240880	243300
lpcA	JW0212	+	243546	244100
yafJ	JW0213	+	244330	245073
yafK	JW0214	-	245086	245802
yafQ	JW0215	-	245982	246236
dinJ	JW0216	-	246263	246499
yafL	JW0217	+	246715	247440
yafM	JW0218	+	247640	248113

■ **SUPPLEMENTARY INFORMATIONS**

25 KB vector DNA sequence. Purple letters: overhang sequences. Letters underlined: mutations.

Fragment 1 (6kb)

CGCAAAATCATGTAGCCAGACGGCATAGGTTTTGCCCGTTTCCTCACCTTTACGATAGGCCGGGCGCGGG
TCCTGCGCCAGTACTTCGCGGATAAACAGCGTTAACTGCGGATAACGCTTCTCCAGCGTCAAAAGCTGCT
TTTCGACCTCTGCGGTAAAACTCACCGCCATCTCTGCAGCTGGCGCACTTTGCGCATAGCTGGCACTGGC
ATCGGGAAGCGATTCGGCAAAGGGGAGATACGGTTTGATATCCACTACCGGCGTACCATCGACCAGATCC
AGACTGCCGAGCTTCAGAATCACGCTGTCTTTATGGCAAACAACCTCTTTCAGCTCTACCAGCGACATGC
CAATTGGGTTAGGGCGGAAAGTAGAGCGTGTTGCGAAAACCCCCATTCTGGCGTTACCGCCGAGGCGCGG
TGGACGCACAGTCGGACGCCAGCCGCCTTCCATCGTTTGATGAAAGACGAAAAGGATCCATAAATGGCTG
AACGCTTCCAGGCCGCGAACGGCGTCGGCCTGGTTGTAGGGAGCAATGAGATGCAGTTCGCCGTTGGCGC
TTTTTACCAGACCTGGCTGGCGCGGAACGGCGAACTTTTCTTTATAGGGCGAGCGAATAACGCCTATTTG
CTCGAACTGGAAACTGCTCATTTCGCCGTAATGTTAAGCGCAGAACCGATACATACAGCCTGACGATAGC
AGCCTGGCGTACCGCTGGTGACTTCGCAGCTATGCAGTAATACAGCATTGGCTTTCATTTTAGAGGCGTT
GATTTGCATCCGCTTACGTGCGGTTGGAATGCTCGGCGGAGAGTCCTGATTAGAGGCCTGGCAAGAGTCG
CCACTGACTTCACCGAGATCGCGGAACGGTTTGCCGACTAATTCTTCTGCATTGGTATAAATTCGGACCG
GCGTGGCGCGCGGCGCTTTCGGTTTTGCAGGCTCCGCTTTCGGCTGGGGTGCAGTGCTTTGAACGGGTTC
GACAGGGGATCTGCTTAACATGGAACAGCCGCTTAGCATGAGTGCTACTAAACAGATCGGTAAAGCACGC
ATAGTATTTCCTCAATGTATGATCAAAACGTCAATATTGAATCAGGAGCTTGTAAAAATGACAAGACGGG
CAAGCGCCCGTCCTGAATGATATTACAAATTGTGGAAACAGCCTAAAAATTACCAGCCTTTAACAGCTCC
GCCGTTAAACACTTTGTTTGCTGCTTCGTAAACTTCGTCAGACTGATAAGCCTGGACGAATTTCTTCACG
TTCTCGGCGTCTTTGTTATCTTCACGCGTCACGATCAGGTTTACGTACGGGGACTCTTTATCTTCAACAA
AGATACCGTCTTTCGCCGGAGTCAGGCCAATCTGGCTGGCATAGGTGGTATTGATAACTGCCAGAGCGAT
TTGCGCGTCGTCCAGAGAACGCGGCAGTTGCGGTGCTTCCAGTTCAACAATTTTCAGATTTTTGGGGTTC
TCAACAACATCAAGAACGGTCGGCAGCAGGCCAACGCCATCTTTCAGTTTGATCAAGCCCACTTTTTGCA
GCAGCAGCAGTGAACGACCAAGGTTAGTTGGGTCGTTTGGCACGGCAACCTGCGAACCATCCTGCAGTTC
ATCCAGTGATTTGATTTTCTTGGAGTAACCAGCAATCGGATAAACAAAAGTGTTGCCTACTGCGACCAGT
TTGTAGCCACGATCTTTCAGTTGCTGATCAAGGTACGGTTTATGCTGGAAGGCGTTGGCGTCGATATCGC
CTTTGCTCAATGCTTCGTTTGGCAGAACATAGTCGTTGAAGGTTACCAGCTCAACGTCCAGGCCATATTT
GTCTTTCGCAACTTTCTGCGCGACTTCTGCAACCTGCTGTTCGGCACCAACAATCACGCCGACTTTAATG
TGGTTTGGATCTTTTTCATCCTGACCGCAGCCTACCAGTGCCAGTGATCCGATCAGGGCTCCCACTGCCG
CAAAGGTTTTGAATTTGAACGCCATCACCACCACCACCACCACTGAGATCCGGCTGCTAACAAAGCCCGA
AAGGAAGCTGAGTTGGCTGCTGCCACCGCTGAGCAATAACTAGCATAACCCCTTGGGGCCTCTAAACGGG
TCTTGAGGGGTTTTTTGCTGAAAGGAGGAACTATATCCGGATTGGCGAATGGGACGCGCCCTGTAGCGGC
GCATTAAGCGCGGCGGGTGTGGTGGTTACGCGCAGCGTGACCGCTACACTTGCCAGCGCCCTAGCGCCCG
CTCCTTTCGCTTTCTTCCCTTCCTTTCTCGCCACGTTCGCCGGCTTTCCCCGTCAAGCTCTAAATCGGGG
GCTCCCTTTAGGGTTCCGATTTAGTGCTTTACGGCACCTCGACCCCAAAAAACTTGATTAGGGTGATGGT
TCACGTAGTGGGCCATCGCCCTGATAGACGGTTTTTCGCCCTTTGACGTTGGAGTCCACGTTCTTTAATA
GTGGACTCTTGTTCCAAACTGGAACAACACTCAACCCTATCTCGGTCTATTCTTTTGATTTATAAGGGAT
TTTGCCGATTTCGGCCTATTGGTTAAAAAATGAGCTGATTTAACAAAAATTTAACGCGAATTTTAACAAA
ATATTAACGTTTACAATTTCAGGTGGCACTTTTCGGGGAAATGTGCGCGGAACCCCTATTTGTTTATTTT
TCTAAATACATTCAAATATGTATCCGCTCATGAGACAATAACCCTGATAAATGCTTCAATAATATTGAAA
AAGGAAGAGTATGAGTATTCAACATTTCCGTGTCGCCCTTATTCCCTTTTTTGCGGCATTTTGCCTTCCT
GTTTTTGCTCACCCAGAAACGCTGGTGAAAGTAAAAGATGCTGAAGATCAGTTGGGTGCACGAGTGGGTT
ACATCGAACTGGATCTCAACAGCGGTAAGATCCTTGAGAGTTTTCGCCCCGAAGAACGTTTTCCAATGAT
GAGCACTTTTAAAGTTCTGCTATGTGGCGCGGTATTATCCCGTATTGACGCCGGGCAAGAGCAACTCGGT
CGCCGCATACACTATTCTCAGAATGACTTGGTTGAGTACTCACCAGTCACAGAAAAGCATCTTACGGATG

GCATGACAGTAAGAGAATTATGCAGTGCTGCCATAACCATGAGTGATAACACTGCGGCCAACTTACTTCT
GACAACGATCGGAGGACCGAAGGAGCTAACCGCTTTTTTGCACAACATGGGGGATCATGTAACTCGCCTT
GATCGTTGGGAACCGGAGCTGAATGAAGCCATACCAAACGACGAGCGTGACACCACGATGCCTGCAGCAA
TGGCAACAACGTTGCGCAAACTATTAACTGGCGAACTACTTACTCTAGCTTCCCGGCAACAATTAATAGA
CTGGATGGAGGCGGATAAAGTTGCAGGACCACTTCTGCGCTCGGCCCTTCCGGCTGGCTGGTTTATTGCT
GATAAATCTGGAGCCGGTGAGCGTGGGTCTCGCGGTATCATTGCAGCACTGGGGCCAGATGGTAAGCCCT
CCCGTATCGTAGTTATCTACACGACGGGGAGTCAGGCAACTATGGATGAACGAAATAGACAGATCGCTGA
GATAGGTGCCTCACTGATTAAGCATTGGTAACTGTCAGACCAAGTTTACTCATATATACTTTAGATTGAT
TTAAAACTTCATTTTTAATTTAAAAGGATCTAGGTGAAGATCCTTTTTGATAATCTCATGACCAAAATCC
CTTAACGTGAGTTTTCGTTCCACTGAGCGTCAGACCCCGTAGAAAAGATCAAAGGATCTTCTTGAGATCC
TTTTTTTCTGCGCGTAATCTGCTGCTTGCAAACAAAAAAACCACCGCTACCAGCGGTGGTTTGTTTGCCG
GATCAAGAGCTACCAACTCTTTTTCCGAAGGTAACTGGCTTCAGCAGAGCGCAGATACCAAATACTGTCC
TTCTAGTGTAGCCGTAGTTAGGCCACCACTTCAAGAACTCTGTAGCACCGCCTACATACCTCGCTCTGCT
AATCCTGTTACCAGTGGCTGCTGCCAGTGGCGATAAGTCGTGTCTTACCGGGTTGGACTCAAGACGATAG
TTACCGGATAAGGCGCAGCGGTCGGGCTGAACGGGGGGTTCGTGCACACAGCCCAGCTTGGAGCGAACGA
CCTACACCGAACTGAGATACCTACAGCGTGAGCTATGAGAAAGCGCCACGCTTCCCGAAGGGAGAAAGGC
GGACAGGTATCCGGTAAGCGGCAGGGTCGGAACAGGAGAGCGCACGAGGGAGCTTCCAGGGGGAAACGCC
TGGTATCTTTATAGTCCTGTCGGGTTTCGCCACCTCTGACTTGAGCGTCGATTTTTGTGATGCTCGTCAG
GGGGGCGGAGCCTATGGAAAAACGCCAGCAACGCGGCCTTTTTACGGTTCCTGGCCTTTTGCTGGCCTTT
TGCTCACATGTTCTTTCCTGCGTTATCCCCTGATTCTGTGGATAACCGTATTACCGCCTTTGAGTGAGCT
GATACCGCTCGCCGCAGCCGAACGACCGAGCGCAGCGAGTCAGTGAGCGAGGAAGCGGAAGAGCGCCTGA
TGCGGTATTTTCTCCTTACGCATCTGTGCGGTATTTCACACCGCATATATGGTGCACTCTCAGTACAATC
TGCTCTGATGCCGCATAGTTAAGCCAGTATACACTCCGCTATCGCTACGTGACTGGGTCATGGCTGCGCC
CCGACACCCGCCAACACCCGCTGACGCGCCCTGACGGGCTTGTCTGCTCCCGGCATCCGCTTACAGACAA
GCTGTGACCGTCTCCGGGAGCTGCATGTGTCAGAGGTTTTCACCGTCATCACCGAAACGCGCGAGGCAGC
TGCGGTAAAGCTCATCAGCGTGGTCGTGAAGCGATTCACAGATGTCTGCCTGTTCATCCGCGTCCAGCTC
GTTGAGTTTCTCCAGAAGCGTTAATGTCTGGCTTCTGATAAAGCGGGCCATGTTAAGGGCGGTTTTTTCC
TGTTTGGTCACTGATGCCTCCGTGTAAGGGGGATTTCTGTTCATGGGGGTAATGATACCGATGAAACGAG
AGAGGATGCTCACGATACGGGTTACTGATGATGAACATGCCCGGTTACTGGAACGTTGTGAGGGTAAACA
ACTGGCGGTATGGATGCGGCGGGACCAGAGAAAAATCACTCAGGGTCAATGCCAGCGCTTCGTTAATACA
GATGTAGGTGTTCCACAGGGTAGCCAGCAGCATCCTGCGATGCAGATCCGGAACATAATGGTGCAGGGCG
CTGACTTCCGCGTTTCCAGACTTTACGAAACACGGAAACCGAAGACCATTCATGTTGTTGCTCAGGTCGC
AGACGTTTTGCAGCAGCAGTCGCTTCACGTTCGCTCGCGTATCGGTGATTCATTCTGCTAACCAGTAAGG
CAACCCCGCCAGCCTAGCCGGGTCCTCAACGACAGGAGCACGATCATGCGCACCCGTGGGGCCGCCATGC
CGGCGATAATGGCCTGCTTCTCGCCGAAACGTTTGGTGGCGGGACCAGTGACGAAGGCTTGAGCGAGGGC
GTGCAAGATTCCGAATACCGCAAGCGACAGGCCGATCATCGTCGCGCTCCAGCGAAAGCGGTCCTCGCCG
AAAATGACCCAGAGCGCTGCCGGCACCTGTCCTACGAGTTGCATGATAAAGAAGACAGTCATAAGTGCGG
CGACGATAGTCATGCCCCGCGCCCACCGGAAGGAGCTGACTGGGTTGAAGGCTCTCAAGGGCATCGGTCG
AGATCCCGGTGCCTAATGAGTGAGCTAACTTACATTAATTGCGTTGCGCTCACTGCCCGCTTTCCAGTCG
GGAAACCTGTCGTGCCAGCTGCATTAATGAATCGGCCAACGCGCGGGGAGAGGCGGTTTGCGTATTGGGC
GCCAGGGTGGTTTTTCTTTTCACCAGTGAGACGGGCAACAGCTGATTGCCCTTCACCGCCTGGCCCTGAG
AGAGTTGCAGCAAGCGGTCCACGCTGGTTTGCCCCAGCAGGCGAAAATCCTGTTTGATGGTGGTTAACGG
CGGGATATAACATGAGCTGTCTTCGGTATCGTCGTATCCCACTACCGAGATATCCGCACCAACGCGCAGC
CC**GGACTCGG**

Fragment 2 (4kb)

GCGTGGCGCGCATTGCGCCCAGCGCCATCTGATCGTTGGCAACCAGCATCGCAGTGGGAACGATGCCCTC
ATTCAGCATTTGCATGGTTTGTTGAAAACCGGACATGGCACTCCAGTCGCCTTCCCGTTCCGCTATCGGC
TGAATTTGATTGCGAGTGAGATATTTATGCCAGCCAGCCAGACGCAGACGCGCCGAGACAGAACTTAATG
GGCCCGCTAACAGCGCGATTTGCTGGTGACCCAATGCGACCAGATGCTCCACGCCCAGTCGCGTACCGTC

TTCATGGGAGAAAATAATACTGTTGATGGGTGTCTGGTCAGAGACATCAAGAAATAACGCCGGAACATTA
GTGCAGGCAGCTTCCACAGCAATGGCATCCTGGTCATCCAGCGGATAGTTAATGATCAGCCCACTGACGC
GTTGCGCGAGAAGATTGTGCACCGCCGCTTTACAGGCTTCGACGCCGCTTCGTTCTACCATCGACACCAC
CACGCTGGCACCCAGTTGATCGGCGCGAGATTTAATCGCCGCGACAATTTGCGACGGCGCGTGCAGGGCC
AGACTGGAGGTGGCAACGCCAATCAGCAACGACTGTTTGCCCGCCAGTTGTTGTGCCACGCGGTTGGGAA
TGTAATTCAGCTCCGCCATCGCCGCTTCCACTTTTTCCCGCGTTTTCGCAGAAACGTGGCTGGCCTGGTT
CACCACGCGGGAAACGGTCTGATAAGAGACACCGGCATACTCTGCGACATCGTATAACGTTACTGGTTTC
ACATTCACCACCCTGAATTGACTCTCTTCCGGGCGCTATCATGCCATACCGCGAAAGGTTTTGCGCCATT
CGATGGTGTCCGGGATCTCGACGCTCTCCCTTATGCGACTCCTGCATTAGGAAGCAGCCCAGTAGTAGGT
TGAGGCCGTTGAGCACCGCCGCCGCAAGGAATGGTGCATGCAAGGAGATGGCGCCCAACAGTCCCCCGGC
CACGGGGCCTGCCACCATACCCACGCCGAAACAAGCGCTCATGAGCCCGAAGTGGCGAGCCCGATCTTCC
CCATCGGTGATGTCGGCGATATAGGCGCCAGCAACCGCACCTGTGGCGCCGGTGATGCCGGCCACGATGC
GTCCGGCGTAGAGGATCGAGATCTCGATCCCGCGAAATTAATACGACTCACTATAGGGGAATTGTGAGCG
GATAACAATTCCCCTCTAGAAATAATTTTGTTTAACTTTAAGAAGGAGATATACATATGAAAAAGTGGTT
ATTAGCTGCAGGTCTCGGTTTAGCACTGGCAACTTCTGCTCAGGCGGCTGACAAAATTGCAATCGTCAAC
ATGGGCAGCCTGTTCCAGCAGGTAGCGCAGAAAACCGGTGTTTCTAACACGCTGGAAAATGAGTTCAAAG
GCCGTGCCAGCGAACTGCAGCGTATGGAAACCGATCTGCAGGCTAAAATGAAAAAGCTGCAGTCCATGAA
AGCGGGCAGCGATCGCACTAAGCTGGAAAAAGACGTGATGGCTCAGCGCCAGACTTTTGCTCAGAAAGCG
CAGGCTTTTGAGCAGGATCGCGCACGTCGTTCCAACGAAGAACGCGGCAAACTGGTTACTCGTATCCAGA
CTGCTGTGAAATCCGTTGCCAACAGCCAGGATATCGATCTGGTTGTTGATGCAAACGCCGTTGCTTACAA
CAGCAGCGATGTAAAAGACATCACTGCCGACGTACTGAAACAGGTTAAATAAGTAATGCCTTCAATTCGA
CTGGCTGATTTAGCGCAGCAGTTGGATGCAGAACTACACGGTGATGGCGATATCGTCATCACCGGCGTTG
CGTCCATGCAATCTGCACAAACAGGTCACATTACGTTCATGGTTAACCCAAAATACCGTGAGCATTTAGG
CTTGTGCCAGGCGTCCGCGGTTGTCATGACCCAGGACGATCTTCCTTTCGCGAAAAGTGCCGCACTGGTA
GTGAAGAATCCCTACCTGACTTACGCGCGCATGGCGCAAATTTTAGATACCACGCCGCAGCCCGCGCAGA
ACATTGCACCCAGTGCGGTGATCGACGCGACGGCGAAGCTGGGTAACAACGTATCGATTGGCGCTAACGC
GGTGATTGAGTCCGGCGTTGAACTGGGCGATAACGTGATTATCGGTGCCGGTTGCTTCGTAGGTAAAAAC
AGCAAAATCGGTGCAGGTTCGCGTCTCTGGGCGAACGTAACCATTTACCATGAGATCCAGATCGGTCAGA
ATTGCCTGATCCAGTCCGGAACAGTGGTAGGCGCAGACGGCTTTGGTTATGCCAACGATCGTGGTAACTG
GGTGAAGATCCCACAGATTGGTCGCGTAATTATTGGCGATCGCGTGGAGATCGGTGCCTGCACAACCATC
GATCGCGGCGCGCTGGATGACACTATTATTGGCAATGGCGTGATCATTGATAACCAGTGCCAGATTGCAC
ATAACGTCGTGATTGGCGACAATACGGCGGTTGCCGGTGGCGTCATTATGGCGGGCAGCCTGAAAATTGG
TCGTTACTGCATGATCGGCGGAGCCAGCGTAATCAACGGGCATATGGAAATATGCGACAAAGTGACGGTT
ACGGGCATGGGTATGGTGATGCGTCCCATCACTGAACCAGGCGTCTATTCCTCAGGCATTCCGCTGCAAC
CCAACAAAGTCTGGCGCAAAACCGCTGCACTGGTGATGAACATTGATGACATGAGCAAGCGTCTGAAATC
GCTTGAGCGCAAGGTTAATCAACAAGACTAACGTTCCATCTTTTGTTCGCCAAACTTTACGGCCTGTCTC
ATTCTTACGATTGCGGCAGGCCGTGTTATTATTGTCGTTTCTTATATTTTGACAGGAAGAGTATCTTGAC
TACTAACACTCATACTCTGCAGATTGAAGAGATTTTAGAACTTCTGCCGCACCGTTTCCCGTTCTTACTG
GTGGATCGCGTGCTGGATTTTGAAGAAGGTCGTTTTCTGCGCGCAGTAAAAAATGTCTCTGTCAATGAGC
CATTCTTCCAGGGCCATTTCCCTGGAAAACCGATTTTCCCGGGTGTGCTGATTCTGGAAGCAATGGCACA
GGCAACAGGTATTCTGGCGTTTAAAAGCGTAGGAAAACTGGAACCGGGTGAGCTGTACTACTTCGCTGGT
ATTGACGAAGCGCGCTTCAAGCGCCCGGTCGTGCCTGGCGATCAAATGATCATGGAAGTCACTTTCGAAA
AAACGCGCCGCGGCCTGACCCGTTTTAAAGGGGTTGCTCTGGTCGATGGTAAAGTAGTTTGCGAAGCAAC
GATGATGTGTGCTCGTAGCCGGGAGGCCTGATACGTGATTGATAAATCCGCCTTTGTGCATCCAACCGCC
ATTGTGGAAGAGGGCGCGTCAATTGGCGCGAACGCACACATTGGTCCTTTTTGTATCGTTGGACCCCATG
TCGAAATTGGTGAGGGTACCGTACTGAAATCTCACGTTGTCGTGAATGGTCATACTAAAATTGGCCGCGA
TAATGAGATTTATCAGTTCGCCTCCATCGGCGAAGTTAACCAGGATCTGAAATATGCTGGCGAACCGACC
CGTGTGGAAATCGGCGATCGTAACCGCATTCGCGAAAGCGTCACCATTCATCGTGGCACAGTCCAGGGCG
GTGGATTGACGAAGGTGGGCAGCGACAACTTACTGATGATCAACGCGCACATTGCGCACGATTGTACGGT
AGGTAACCGCTGTATTCTCGCCAACAACGCAACGCTGGCGGGTCACGTATCGGTTGACGACTTCGCGATC
ATCGGCGGCATGACCGCAGTCCATCAGTTCTGCATCATTGGTGCGCACGTGATGGTTGGCGGCTGCTCCG
GTGTGGCGCAGGACGTCCCTCCTTATGTCATTGCGCAGGGTAACCACGCAACGCCGTTCGGTGTCAATAT

CGAAGGGCTGAAGCGCCGCGGATTCAGCCGTGAGGCGATTACCGCTATCCGCAATGCGTATAAGCTGATT
TATCGTAGCGGTAAAACGCTCGATGAAGTGAAACCGGAAATTGCTGAACTGGCGGAAACATATCCGGAAG
TGAAAGCCTTTACCGATTTCTTTGCACGCTCAACGCGCGGTCTGATTCGTTAATGACTGAACAGCGTCCA
TTAACGATTGCCCTGGTCGCCGGAGAAACCTCCGGCGATATCCTGGGGGCCGGTTTAATCCGC**GCTCTGA
AAG**

Fragment 3 (5kb)

TTGATGTGCCCAACGCCCGCTTTGTTGGTGTTGCCGGGCCACGAATGCAGGCTGAAGGCTGCGAAGCCTG
GTACGAAATGGAAGAACTGGCGGTGATGGGCATTGTTGAAGTGCTCGGTCGTCTGCGTCGCTTACTGCAT
ATTCGTGCCGATCTGACAAAGCGTTTTGGCGAACTGAAGCCAGATGTTTTTGTTGGTATTGATGCGCCTG
ACTTCAATATTACTCTTGAAGGTAACCTCAAAAAGCAGGGTATCAAAACCATTCATTACGTCAGTCCGTC
AGTCTGGGCGTGGCGACAGAAACGTGTTTTCAAAATAGGCAGAGCCACCGATCTGGTGCTCGCATTTCTG
CCTTTCGAAAAAGCGTTTTATGACAAATACAACGTACCGTGCCGCTTTATCGGTCATACCATGGCTGATG
CCATGCCATTAGATCCAGATAAAAATGCCGCCCGTGATGTGCTGGGGATCCCTCACGATGCCCACTGCCT
GGCGTTGCTACCGGGGAGCCGTGGTGCAGAAGTTGAAATGCTTAGTGCCGATTTCCTGAAAACGGCCCAG
CTTTTGCGCCAGACATATCCGGATCTCGAAATCGTGGTGCCACTGGTGAATGCCAAACGCCGCGAGCAGT
TTGAACGCATCAAAGCTGAAGTCGCGCCAGACCTTTCAGTTCATTTGCTGGATGGGATGGGCCGTGAGGC
GATGGTCGCCAGCGATGCGGCGCTACTGGCGTCGGGTACGGCAGCCCTGGAGTGTATGCTGGCGAAATGC
CCGATGGTGGTGGGATATCGCATGAAGCCTTTTACCTTCTGGTTGGCGAAGCGGCTGGTGAAAACTGATT
ATGTCTCGCTGCCAAATCTGCTGGCGGGCAGAGAGTTAGTCAAAGAATTATTGCAGGAAGAGTGTGAGCC
GCAAAAACTGGCTGCGGCGCTGTTACCGCTGTTGGCGAACGGGAAAACCAGCCACGCGATGCACGATACC
TTCCGTGAACTGCATCAGCAGATCCGCTGCAATGCCGATGAGCAGGCGGCACAAGCCGTTCTGGAGTTAG
CACAATGATCGAATTTGTTTATCCGCACACGCAGCTGGTTGCGGGTGTGGATGAAGTCGGACGCGGGCCG
TTAGTTGGCGCGGTCGTCACCGCTGCGGTGATCCTTGACCCGGCGCGCCCGATTGCCGGGCTGAATGATT
CCAAAAAGCTGAGCGAAAAACGCCGTCTGGCGCTCTATGAAGAGATCAAAGAGAAAGCGTTGAGCTGGAG
TCTGGGCCGCGCGGAACCCCACGAAATCGACGAGCTGAACATTCTTCATGCGACCATGCTGGCGATGCAG
CGTGCCGTCGCTGGGCTGCATATTGCGCCGGAATATGTGTTGATTGATGGTAACCGCTGCCCGAAATTAC
CGATGCCTGCGATGGCTGTGGTGAAAGGCGATAGCCGCGTACCGGAAATCAGTGCCGCGTCTATCCTGGC
GAAAGTGACGCGTGACGCCGAAATGGCGGCGCTGGATATTGTTTTCCCGCAATATGGTTTTGCCCAACAC
AAAGGGTACCCAACCGCTTTTCATCTGGAAAAACTGGCTGAACACGGCGCGACCGAACACCATCGGCGCA
GCTTTGGGCCTGTCAAACGCGCACTGGGACTTGCGTCCTGATTCTTGTGTCGAGATTAAGTAAACCGGAA
TCTGAAGATGTCTGAACCACGTTTCGTACACCTGCGGGTGCACAGCGACTACTCGATGATCGATGGCCTG
GCCAAAACCGCACCGTTGGTAAAAAAGGCGGCGGCGTTGGGTATGCCAGCACTGGCGATCACCGATTTCA
CCAACCTTTGTGGTCTGGTGAAGTTCTACGGAGCGGGACATGGCGCAGGGATTAAGCCTATCGTCGGGGC
AGATTTTAACGTCCAGTGCGACCTGCTGGGTGATGAGTTAACCCACCTGACGGTACTGGCGGCGAACAAT
ACCGGCTATCAGAATCTGACGTTGCTGATCTCAAAAGCGTATCAGCGCGGGTACGGTGCCGCCGGGCCGA
TCATCGATCGCGACTGGCTTATCGAATTAAACGAAGGGTTGATCCTTCTTTCCGGCGGACGCATGGGCGA
CGTCGGACGCAGTCTTTTGCGTGGTAACAGCGCGCTGGTAGATGAGTGTGTCGCGTTTTATGAAGAACAC
TTCCCGGATCGCTATTTTCTCGAGCTGATCCGCACCGGCAGGCCGGATGAAGAAAGCTATCTGCACGCGG
CGGTGGAACTGGCGGAAGCGCGCGGTTTGCCCGTCGTGGCGACCAACGACGTGCGCTTTATCGACAGCAG
CGACTTTGACGCACACGAAATCCGCGTCGCGATCCACGACGGCTTTACCCTCGACGATCCTAAACGCCCG
CGTAACTATTCGCCGCAGCAATATATGCGTAGCGAAGAGGAGATGTGTGAGCTGTTTGCCGACATCCCCG
AAGCCCTTGCCAACACCGTTGAGATCGCCAAACGCTGTAACGTAACCGTGCGTCTTGGTGAATACTTCCT
GCCGCAGTTCCCGACCGGGGACATGAGCACCGAAGATTATCTGGTCAAGCGTGCAAAAGAGGGCCTGGAA
GAGCGTCTGGCCTTTTTATTCCCTGATGAGGAAGAACGTCTTAAGCGCCGCCCGGAATATGACGAACGTC
TGGAGACTGAACTTCAGGTTATCAACCAGATGGGCTTCCCGGGCTACTTCCTCATCGTTATGGAATTTAT
CCAGTGGTCGAAAGATAACGGCGTACCGGTAGGGCCAGGCCGTGGCTCCGGTGCGGGTTCACTGGTGGCC
TACGCGCTGAAAATCACCGACCTCGATCCGCTGGAATTTGACCTGCTGTTCGAACGTTTCCTTAACCCGG
AACGTGTCTCCATGCCTGACTTCGACGTTGACTTCTGTATGGAGAAACGCGATCAGGTTATCGAGCACGT
AGCGGACATGTACGGTCGTGATGCGGTATCGCAGATCATCACCTTCGGTACAATGGCGGCGAAAGCGGTG

ATCCGCGACGTAGGCCGCGTGCTGGGGCATCCGTACGGCTTTGTCGATCGTATCTCGAAACTGATCCCGC
CCGATCCGGGGATGACGCTGGCGAAAGCGTTTGAAGCCGAGCCGCAGCTGCCGGAAATCTACGAAGCGGA
TGAAGAAGTTAAGGCGCTGATCGACATGGCGCGCAAACTGGAAGGGGTCACCCGTAACGCCGGTAAGCAC
GCCGGTGGGGTGGTTATCGCGCCGACCAAAATTACCGATTTTGCGCCGCTTTACTGCGATGAAGAGGGCA
AACATCCGGTCACCCAGTTTGATAAAAGCGACGTTGAATACGCCGGACTGGTGAAGTTCGACTTCCTTGG
TTTGCGTACGCTCACCATCATCAACTGGGCGCTGGAGATGATCAACAAGCGGCGGGCGAAGAATGGCGAG
CCGCCGCTGGATATCGCTGCGATCCCGCTGGATGATAAGAAAAGCTTCGACATGCTGCAACGCTCGGAAA
CCACGGCGGTATTCCAGCTTGAATCGCGCGGCATGAAGGACCTGATCAAGCGTCTACAACCTGACTGCTT
CGAAGATATGATCGCCCTAGTGGCACTGTTCCGCCCCGGTCCGTTGCAATCAGGGATGGTGGATAACTTT
ATCGACCGTAAACATGGTCGTGAAGAGATCTCCTATCCGGACGTACAGTGGCAGCATGAAAGCCTGAAAC
CGGTACTGGAGCCAACCTACGGCATTATCCTGTATCAGGAACAGGTCATGCAGATTGCGCAGGTGCTTTC
TGGTTATACCCTCGGTGGCGCGGATATGCTGCGTCGTGCGATGGGTAAGAAAAAGCCGGAAGAGATGGCT
AAGCAACGTTCTGTATTTGCTGAAGGTGCAGAAAAGAACGGAATCAACGCTGAACTGGCGATGAAAATCT
TCGACCTGGTGGAGAAATTCGCTGGTTACGGATTTAACAAATCGCACTCTGCGGCCTATGCTTTGGTGTC
ATATCAAACGTTATGGCTGAAAGCGCACTATCCTGCGGAGTTTATGGCGGCGGTAATGACCGCCGATATG
GACAACACCGAGAAGGTGGTGGGTCTGGTGGATGAGTGCTGGCGGATGGGGCTGAAAATCCTGCCACCAG
ATATAAACTCCGGTCTTTACCATTTCCACGTCAACGACGACGGCGAAATCGTGTATGGTATTGGCGCGAT
CAAAGGGGTCGGTGAAGGTCCGATTGAGGCCATCATCGAAGCCCGTAATAAAGGCGGCTACTTCCGCGAA
CTGTTTGATCTCTGCGCCCGTACCGACACCAAAAAGTTGAACCGTCGCGTGCTGGAAAAACTGATCATGT
CCGGGGCGTTTGACCGTCTTGGGCCACATCGCGCAGCGCTGATGAACTCGCTGGGCGATGCGTTAAAAGC
GGCAGATCAACACGCGAAAGCGGAAGCTATCGGTCAGGCCGATATGTTCGGCGTGCTGGCCGAAGAGCCG
GAACAAATTGAACAATCCTACGCCAGCTGCCAACCGTGGCCGGAGCAGGTGGTATTAGATGGGGAACGTG
AAACGTTAGGCCTGTACCTGACCGGACACCCTATCAACCAGTATTTAAAAGAGATTGAGCGTTATGTCGG
AGGCGTAAGGCTGAAAGACATGCACCCGACAGAACGTGGTAAAGTCATCACGGCTGCGGGGCTCGTTGTT
GCCGCGCGGGTTATGGTCACCAAGCGCGGCAATCGTATCGGTATCTGCACGCTGGATGACCGTTCCGGGC
GGCTGGAAGTGATGTTGTTTACTGACGCCCTGGATAAATACCAGCAATTGCTGGAAAAAGACCGCATACT
TATCGTCAGCGGACAGGTCAGCTTTGATGACTTCAGCGGTGGGCTTAAAATGACCGCTCGCGAAGTGATG
GATATTGACGAAGCCCGGGAAAAAATATGCTCGCGGGCTTGCTATCTCGCTGACGGACAGGCAAATTGATG
ACCAGCTTTTAAACCGACTCCGTCAGTCTCTGGAACCCCACCGCTCTGGGACAATTCCAGTACATCTCTA
CTATCAGAGGGCGGATGCACGCGCGCGGTTGCGTTTTGGCGCGACGTGGCGTGTCTCTCCGAGCGATCGT
TTATTAAACGATCTCCGTGGCCTCATTGGTTCGGAGCAGGTGGAACTGGAGTTTGACTAATACAG**GAATA**
CTATG

Fragment 4 (6kb)

TCGCTGAATTTCCTTGATTTTGAACAGCCGATTGCAGAGCTGGAAGCGAAAATCGATTCTCTGACTGCGG
TTAGCCGTCAGGATGAGAAACTGGATATTAACATCGATGAAGAAGTGCATCGTCTGCGTGAAAAAAGCGT
AGAACTGACACGTAAAATCTTCGCCGATCTCGGTGCATGGCAGATTGCGCAACTGGCACGCCATCCACAG
CGTCCTTATACCCTGGATTACGTTCGCCTGGCATTTGATGAATTTGACGAACTGGCTGGCGACCGCGCGT
ATGCAGACGATAAAGCTATCGTCGGTGGTATCGCCCGTCTCGATGGTCGTCCGGTGATGATCATTGGTCA
TCAAAAAGGTCGTGAAACCAAAGAAAAAATTCGCCGTAACTTTGGTATGCCAGCGCCAGAAGGTTACCGC
AAAGCACTGCGTCTGATGCAAATGGCTGAACGCTTTAAGATGCCTATCATCACCTTTATCGACACCCCGG
GGGCTTATCCTGGCGTGGGCGCAGAAGAGCGTGGTCAGTCTGAAGCCATTGCACGCAACCTGCGTGAAAT
GTCTCGCCTCGGCGTACCGGTAGTTTGTACGGTTATCGGTGAAGGTGGTTCTGGCGGTGCGCTGGCGATT
GGCGTGGGCGATAAAGTGAATATGCTGCAATACAGCACCTATTCCGTTATCTCGCCGGAAGGTTGTGCGT
CCATTCTGTGGAAGAGCGCCGACAAAGCGCCGCTGGCGGCTGAAGCGATGGGTATCATTGCTCCGCGTCT
GAAAGAACTGAAACTGATCGACTCCATCATCCCGGAACCACTGGGTGGTGCTCACCGTAACCCGGAAGCG
ATGGCGGCATCGTTGAAAGCGCAACTGCTGGCGGATCTGGCCGATCTCGACGTGTTAAGCACTGAAGATT
TAAAAAATCGTCGTTATCAGCGCCTGATGAGCTACGGTTACGCGTAATTCGCAAAAGTTCTGAAAAAGGG
TCACTTCGGTGGCCCTTTTTTATCGCCACGGTTTGAGCAGGCTATGATTAAGGAAGGATTTTCCAGGAGG
AACACATGAACATCATTGCCATTATGGGACCGCATGGCGTCTTTTATAAAGATGAGCCCATCAAAGAACT

GGAGTCGGCGCTGGTGGCGCAAGGCTTTCAGATTATCTGGCCACAAAACAGCGTTGATTTGCTGAAATTT
ATCGAGCATAACCCTCGAATTTGCGGCGTGATTTTTGACTGGGATGAGTACAGTCTCGATTTATGTAGCG
ATATCAATCAGCTTAATGAATATCTCCCGCTTTATGCCTTCATCAACACCCACTCGACGATGGATGTCAG
CGTGCAGGATATGCGGATGGCGCTCTGGTTTTTTGAATATGCGCTGGGGCAGGCGGAAGATATCGCCATT
CGTATGCGTCAGTACACCGACGAATATCTTGATAACATTACACCGCCGTTCACGAAAGCCTTGTTTACCT
ACGTCAAAGAGCGGAAGTACACCTTTTGTACGCCGGGGCATATGGGCGGCACCGCATATCAAAAAAGCCC
GGTTGGCTGTCTGTTTTATGATTTTTTCGGCGGGAATACTCTTAAGGCTGATGTCTCTATTTCGGTCACC
GAGCTTGGTTCGTTGCTCGACCACACCGGGCCACACCTGGAAGCGGAAGAGTACATCGCGCGGACTTTTG
GCGCGGAACAGAGTTATATCGTTACCAACGGAACATCGACGTCGAACAAAATTGTGGGTATGTACGCCGC
GCCATCCGGCAGTACGCTGTTGATCGACCGCAATTGTCATAAATCGCTGGCGCATCTGTTGATGATGAAC
GATGTAGTGCCAGTCTGGCTGAAACCGACGCGTAATGCGTTGGGGATTCTTGGTGGGATCCCGCGCCGTG
AATTTACTCGCGACAGCATCGAAGAGAAAGTCGCTGCTACCACGCAAGCACAATGGCCGGTTCATGCGGT
GATCACCAACTCCACCTATGATGGCTTGCTCTACAACACCGACTGGATCAAACAGACGCTGGATGTCCCG
TCGATTCACTTCGATTCTGCCTGGGTGCCGTACACCCATTTTCATCCGATCTACCAGGGTAAAAGTGGTA
TGAGCGGCGAGCGTGTTGCGGGAAAAGTGATCTTCGAAACGCAATCGACCCACAAAATGCTGGCGGCGTT
ATCGCAGGCTTCGCTGATCCACATTAAAGGCGAGTATGACGAAGAGGCCTTTAACGAAGCCTTTATGATG
CATACCACCACCTCGCCCAGTTATCCCATTGTTGCTTCGGTTGAGACGGCGGCGGCGATGCTGCGTGGTA
ATCCGGGCAAACGGCTGATTAACCGTTCAGTAGAACGAGCTCTGCATTTTCGCAAAGAGGTCCAGCGGCT
GCGGGAAGAGTCTGACGGTTGGTTTTTCGATATCTGGCAACCGCCGCAGGTGGATGAAGCCGAATGCTGG
CCCGTTGCGCCTGGCGAACAGTGGCACGGCTTTAACGATGCGGATGCCGATCATATGTTTCTCGATCCGG
TTAAAGTCACTATTTTGACACCGGGGATGGACGAGCAGGGCAATATGAGCGAGGAGGGGATCCCGGCGGC
GCTGGTAGCAAAATTCCTCGACGAACGTGGGATCGTAGTAGAGAAAACCGGCCCTTATAACCTGCTGTTT
CTCTTTAGTATTGGCATCGATAAAACCAAAGCAATGGGATTATTGCGTGGGTTGACGGAATTCAAACGCT
CTTACGATCTCAACCTGCGGATCAAAAATATGCTACCCGATCTCTATGCAGAAGATCCCGATTTCTACCG
CAATATGCGTATTCAGGATCTGGCACAAGGGATCCATAAGCTGATTCGTAAACACGATCTTCCCGGTTTG
ATGTTGCGGGCATTCGATACTTTGCCGGAGATGATCATGACGCCACATCAGGCATGGCAACGACAAATTA
AAGGCGAAGTAGAAACCATTGCGCTGGAACAACTGGTCGGTAGAGTATCGGCAAATATGATCCTGCCTTA
TCCACCGGGCGTACCGCTGTTGATGCCTGGAGAAATGCTGACCAAAGAGAGCCGCACAGTACTCGATTTT
CTACTGATGCTTTGTTCCGTCGGGCAACATTACCCCGGTTTTGAAACGGATATTCACGGCGCGAAACAGG
ACGAAGACGGCGTTTACCGCGTACGAGTCCTAAAAATGGCGGGATAACTTGCCAGAGCGGCTTCCGGGCG
AGTAACGTGCTGTTAACAAATAAAGGAGACGTTATGCTGGGTTTAAAACAGGTTCACCATATTGCGATTA
TTGCGACGGATTATGCGGTGAGCAAAGCTTTCTACTGCGATATTCTTGGTTTCACGCTGCAAAGCGAAGT
CTATCGCGAAGCGCGCGACTCATGGAAAGGGGATTTGGCGCTTAATGGGCAATATGTGATTGAGCTTTTC
TCATTTCCGTTCCCGCCGGAACGACCCAGCCGACCGGAAGCTTGCGGTCTGCGTCATCTGGCTTTTAGCG
TTGATGACATCGATGCGGCAGTGGCGCACCTTGAAAGCCATAACGTGAAGTGTGAAACCATCCGTGTCGA
TCCATACACGCAAAAACGCTTCACCTTCTTTAACGATCCGGACGGGCTGCCGTTGGAACTGTATGAGCAG
TAAGGCTTGTCATCGCCGCATTTGCCCGGTAACGTGCCGGGCATTGCTACTGTAAAATCGCACCATCATG
ACACTCACGCTCAATAGACAACTTCTCACCTCACGCCAGATTCTGGTGGCCTTTAGCGGCGGGCTTGACT
CCACCGTTCTGCTGCATCAGTTGGTGCAGTGGCGGACGGAAAATCCGGGTGTCGCTCTGCGCGCTATTCA
TGTGCATCACGGTTTAAGTGCCAATGCCGATGCCTGGGTTACGCATTGCGAAAACGTCTGCCAACAGTGG
CAGGTGCCGCTGGTGGTCGAACGCGTACAACTTGCGCAAGAAGGACTGGGCATTGAGGCCCAGGCGCGGC
AGGCACGTTATCAGGCATTTGCCCGCACCTTGTTGCCCGGTGAAGTGCTGGTCACCGCGCAACATCTCGA
CGATCAATGTGAAACCTTTCTGCTGGCGCTAAAACGCGGCAGTGGCCCTGCCGGGCTTTCGGCTATGGCG
GAAGTCTCGGAGTTTGCCGGAACGCGGCTTATTCGCCCGTTGCTCGCCCGCACGCGGGGGGAACTGGTGC
AGTGGGCGCGTCAGTATGATTTACGCTGGATTGAAGACGAAAGTAATCAGGACGACTCATACGATCGTAA
CTTTCTGCGCCTGCGCGTAGTGCCGTTATTGCAGCAGCGTTGGCCGCATTTTGCCGAAGCAACGGCCCGC
AGCGCCGCACTTTGTGCTGAACAAGAGAGCCTGCTGGATGAACTGCTGGCAGATGATTTAGCACACTGTC
AATCGCCGCAGGGGACGCTGCAGATTGTGCCAATGCTGGCGATGAGTGATGCCCGCCGCGCGGCGATTAT
CCGCCGCTGGCTGGCAGGGCAGAATGCACCGATGCCTTCCCGCGACGCGTTGGTGAGGATCTGGCAGGAA
GTGGCGCTGGCGCGGGAAGATGCCTCACCCTGTTTACGTTTGGGCGCGTTTGAAATCCGACGCTATCAGT
CGCAACTGTGGTGGATTAAATCCGTCACCGGGCAAAGCGAAAACATTGTGCCGTGGCAGACGTGGCTTCA
ACCGCTGGAATTACCGGCGGGGCTGGGAAGTGTACAGCTTAATGCGGGAGGCGATATTCGCCCTCCGCGT

GCAGACGAAGCGGTCAGCGTGCGTTTCAAAGCGCCAGGATTGCTGCATATTGTCGGGCGTAACGGCGGAC
GTAAGCTAAAGAAAATCTGGCAAGAGCTGGGCGTGCCGCCGTGGCTACGTGACACCACGCCACTGCTGTT
TTATGGCGAAACGCTGATTGCGGCGGCAGGGGTATTTGTGACGCAAGAAGGTGTGGCTGAAGGTGAGAAT
GGCGTAAGTTTTGTCTGGCAGAAAACGCTTAGTTAAGTGAAAGCCGGATAAGACGCATCAAACGTCGCAT
CCGGCGAAAGTCAATCAGGATTCGCTTACCACCACCGTACCGATTTCCGGGTGGCTAAAGCTGGTAATTT
TATCCAGACGCAGCTCGCGGGTTTCGCCAGCGGCCTCGACGACCAGGTACTCCACATTTTTGCGGGAGAC
TAAATCACTGGCTTTTGCCTGCAATTTTTCGCCATCTTTCAGCTCAAGTGTCAGCATTAAATGATGCTGG
CAGGCGAGCTCAAGATTATCGTAATCATCACAATTGATTGGTTGATACGTATCATTCATTGACATAATCG
CTCACCAGTAAGTTTGCCGCAGCGTATGCTGCTTTTTCCCTGACAGCCTCAGAAAGGGCGTCGTCGGCAG
CCATTTCATTCAGCACTTTCAAAACGCAGCCCAGCGCGTCCGGAACGTATCCTAAGTCTCCGCTGGCGAT
TTCCGCGTACCGCTTGCGTATTAACTCACAATATTTTTCCACATGCCCTCCTGTCAGCACTCTGACTTAA
CCGTGGATGCAAGTCTAAGCCTACGAAGATAAACTCTGTTTCGCAAGGTGACTATACCACACTCATTTCT
GCAATATCAGCGCCGCAACTGCACGTATTCCGTTACAATGGCCTCCTGATTCGAAAGGAGTTTTCTTATG
GCGCTTAAAGCGACAATTTATAAAGCGACGGTTAATGTGGCCGATCTCGACCGCAACCAGTTTCTCGATG
CCTCTCTGACGCTGGCGCGCCATCCTTCAGAAACCCAGGAGCGTATGATGCTGCGCTTGCTGGCGTGGCT
GAAATATGCTGATGAACGTCTGCAATTTACCCGTGGTTTGTGTGCCGATGATGAGCCGGAAGCGTGGCTG
CGTAACGATCACCTGGGCATTGATTTGTGGATTGAGCTGGGGCTGCCGGATGAGCGGCGGATTAAGAAAG
CCTGCACCCAGGCCGCAGAAGTGGCGCTGTTTACCTATAATAGTCGGGCGGCGCAAATCTGGTGGCAGCA
AAATCAGAGCAAATGTGTGCAGTTTGCCAATCTTTCCGTCTGGTATCTGGACGATGAACAACTGGCGAAA
GTAAGCGCCTTTGCCGATCGTACCATGACGCTGCAGGCAACGATTCAGGATGGCGTGATCTGGTTATCGG
ATGATAAGAATAATCTGGAAGTGAACTTAACCG**CCTGGCAAC**

Fragment 5 (4kb)

GGACTTCATGATTGTGATTTCCCGACATGTTGCTATTCCCGATGGTGAGCTTGAGATCACCGCCATTCGT
GCGCAGGGCGCGGGCGGGCAGCATGTTAATAAGACCTCAACGGCTATTCATCTGCGTTTTGACATTCGGG
CGTCCAGCCTGCCAGAGTATTACAAAGAGCGTCTGCTCGCCGCCAGCCATCATTTGATCAGCAGTGATGG
CGTGATTGTCATTAAGGCACAGGAATACCGCAGTCAGGAACTGAACCGCGAAGCAGCTCTGGCCCGGCTG
GTGGCTATGATTAAAGAATTAACAACAGAAAAAAAAGCCCGACGACCCACGCGGCCCACCCGTGCATCGA
AAGAGCGCAGGCTGGCATCGAAAGCACAAAAATCAAGCGTGAAGGCGATGCGCGGCAAAGTGCGCAGCGG
TCGGGAATAAAAAGAAGGAATGGATGGTGAAAAAAGCGATAGTGACAGCGATGGCTGTAATCAGCCTCTT
TACTCTGATGGGATGTAATAATCGGGCCGAAGTCGATACGCTTTCTCCGGCGCAGGCTGCCGAACTGAAA
CCGATGCCGCAAAGTTGGCGCGGCGTGCTGCCGTGTGCCGATTGCGAAGGAATCGAAACCTCTCTGTTCC
TCGAAAAAGACGGAACATGGGTGATGAATGAGCGTTATCTCGGTGCTCGTGAAGAACCTTCCTCCTTCGC
TTCCTACGGTACATGGGCGCGAACCGCTGACAAGCTGGTATTAACCGACAGCAAAGGTGAAAAGTCATAT
TATCGGGCGAAAGGCGATGCGCTGGAGATGCTCGATCGTGAAGGCAATCCGATTGAATCGCAGTTCAACT
ATACGCTGGAAGCGGCACAATCCAGTTTACCTATGACGCCGATGACCCTGCGGGGCATGTATTTTTATAT
GGCTGATGCGGCGACCTTCACTGATTGCGCGACCGGAAAACGTTTCATGGTAGCGAATAACGCAGAGCTG
GAGCGTAGCTACCTGGCTGCGCGCGGTCACAGTGAAAAACCGGTGTTACTGTCAGTAGAAGGTCACTTTA
CGCTTGAGGGTAATCCGGATACCGGTGCGCCGACTAAAGTATTGGCACCCGATACGGCAGGGAAATTTTA
CCCCAACCAGGATTGCAGTAGTTTGGGGCAGTAACCCGTCTTGAGACAGAAACAAACGCAAAACAGGCCA
GAAGGATATATTTCAACATTTTGAATTTGCACGTTTTTTGTAGGCCGGATAAGGCGTTTACGCCGCATCC
GGCAATGGTGCTCAACGCCTGATGCGACGCTGGCGCGTCTTATCATGCCTACAACCCCCCTCATACCTTA
AGGCGGATAAGGCATTTACTTTATCACCGGGTCAAACCAACAAAGCGACCCGCCTTGATATAAATCCCAG
GCTTCAGGTGCCCAACATACTGTAACTGCGTTTCTGGCTTAAACGCAGAGACATCACCAATACGCATATG
CATCAGATCGGCAGGGCTAATCCAGCCCGATTGCGCCAGTGTAAGTGGATGCCCGGCTTTGGCAAAGGCA
TCCGTGACAAATTCCGAACAAAACCACGACTTTTTGTCTCCTTCGCCCACACTGCTTAACTGCGCTTTCG
CCAGGCCGCTGACGCACTGTTGGCGAAAATCCTCGGAGAACGGATTCAGTGAGCACATCTGGCGAGTCAC
CATAAAGGGAATAAATTCGACAATGCCGCGATAGTTATAACCGCTATCTTTGATTTTATTGGCAAACGCG
GTGATATCTGTGGCTTGTTGCGGGGTAAGATCCGGGACTCGTAAGACGAAAAGCTTATCACTATGCTTCA
TCGCTTTTTTAAGGGAAACGATCTGGACGCCAGCGCCTGTCGCTTCTGCAACGTTATTATCACCCAGAAA

GATTGCAACGTGACTCACAGAGGAAGTGCTGAAGACGCGGATTCCAAATGAGGTTACCCCAAGGCTTGAG
GAGAACAGCAAATCGCCGGGTTTGAGATCAGGTGCCGTTATTTCTTTTATTGATTGTTCGGTGAAAGAGC
TTTGATGCTGGAATTTGACAGCCCATGTTTTTGCCTCGGCATCTACTGCTGTGGCTGATGGGTCTGGCTG
GCTGATATCAACGGTACAAGCCGAAAGTAAAAGAAAGCTGGGGAGAAACAGGCGGCAGTACGCCTTTGGT
TTATCCATTTTATACAATCCATGTAAAAAAAGGGCCCTGAAATTCAGGACCCTTTCTGGCATCAGCCTTT
AATCTGTTTCACCAGATATTCGACGATGTCACCAGTCTTAATTAACTGTTTCTCGCCGTTGCGACGATAT
TTATATTCGATATCGTCGTTGTCGAGGTTACGGTCGCCCAGCACAATAGTGTGCGGAATACCGATCAGTT
CCATATCAGCAAACATCACGCCCGGACGCTCTTTGCGGTCATCCAGCAGCACTTCGATACCTTGTGCACG
CAGTTCGCTGTACAGTTTCTCAGCAAGCTCTTGTACGCGGAAGGATTTGTGCATGTTCATCGGCAGAATC
GCCACCTGGAACGGCGCGATAGCGTCAGGCCATACGATGCCTCGTTCGTCGTAGTTCTGCTCAATCGCCG
CAGCTACCACACGCGTTACCCCGATACCGTAGCAACCCATCGTCAGGATTTGGTTACGGCCATCTTCACC
CTGTACGGAGGCTTTCAGTGCTTCGGAGTACTTGGTACCCAGCTGGAAGATGTGACCAACTTCGATACCA
CGTTTGATCAGCAGCCTACCCTGGCCATCCGGGCTTGGATCGCCAGCCACCACGTTACGGATATCTGCAA
CTTCCGGGGTAGCGACATCGCGATCCCAGTTGATGCCGAAGTAGTGTTTACCATCGATGTTAGCACCAGC
AGCGAAATCACTCATCGCCGCAACGGTACGGTCAATCACCACCGGAATCGGCATGTTTACCGGACCCAGT
GAACCCGGACCGGCTTTAACCACGGCACGAATTTCTTCTTCGGTCGCGAAAGTCAGCGGGCTTGCAACCT
GCGGCAGTTTTTCTGCTTTAACTTCGTTCAGCTCGTGATCACCGCGCACCAGCAGCGCAACCTGCGGGAA
GCTGCTGCCTTCAACCGCTTTAACCAGCAGAGTCTTAACCGTTTTCTCAATCGGCAGATTGAACTGTTCA
ACCAGTTCCGCGATGGTTTTCGCGTTCGGCGTATCAACCAGCGTCATTTCCTGGGTAGCAGCAGCGCGCG
GTTCTTTCGGCGCGATAGCTTCTGCCAGTTCAATGTTCGCTGCATAGTCAGAGGTGTCGGAGAAGACCAC
ATCGTCTTCACCGCTCTGCGCCAGCACCTGGAATTCGTGAGAGGCGCTGCCGCCGATAGAACCGGTGTCG
GCTTGTACGGCGCGGAAATCCAGCCCCATGCGGCTGAAGATTTTGCTGTAGGCCGCATACATTGCATCGT
AGGTTTCCTGCAGGGATTCCTGAGAAGTATGGAAAGAGTAAGCATCTTTCATCAGGAATTCGCGGGAACG
CATGACGCCGAAACGCGGACGCACTTCGTCGCGGAACTTGGTCTGGATCTGATAGAAGTTCAGCGGCAGC
TGTTTGTAAGAGCTAAGCTCGTTACGAATCAGGTCAGTGATAACTTCTTCATGAGTTGGGCCGAGTACGA
ACGGACGCTCGCCACGGTCAACAAAACGCAGCAGTTCCGGACCGTACTGTTCCCAACGACCACTCTCTTG
CCACAAATCGGCTGGCTGAACCACCGGCATCGACACCTCGATCGCACCGGCGTTGTTCATCTCTTCACGC
ACGATGTTTTCGACTTTTTTCAGAACGCGCACGCCGGTCGGCAGCCAGGTATATAACCCGGAGGCCAGCT
TGCGGATCATCCCGGCGCGCAGCATCAGCTGATGGCTGATCACCTCGGCGTCGGCAGGTGTCTCCTTGAG
AGTGGAGAGCAGGTATTGGCTAGTACGCATGTTGTTACGGTTCCAGTTGGAAGGTAGAACAGGCTCAAGG
CGAGCCTGGGACAAAAAAAAGTGATTTAGTTTACCAGTGCAAAAGAAATGTCAAAAGAGAAGGGCGTGAA
TTTAACGCGGTTCCAGCGCAAAGACTTCAAAACCTGCGTCGGTGACGCG**CCAGCGAAC**

50 KB vector DNA sequence. Purple letters: overhang sequences. Letters underlined: mutations.

Fragment 1 (6kb)

ACCACATCACATATCTCTTTCAGCTACGCCGCCCGCCAGCGGATGCAAAACCGTGCGCGTTTATTAAAAC
AGTACCAAACTCATCTGAAAAAGCAGGCCAGCTATATTGTGGAAGGCAATGCCGAAAGCAAAAGGGCGCT
ACGCCAGCACAACCGGGAGCAGATAAAACAGCATCCAGAATGGTTTCCTGCTCCGCTCAAGGCGAGTGAC
AGACGCTGGCAGGCGCTGGCGGAAAACAACCACTTTTTAAGCAGCGACCATCTGCATAACATTACCGAAG
TGGCGATTCACCGCCTGGAGCAGCAGCTTGGCAAGCCTTACGTCTGGGGCGGTACGCGGCCTGATAAAGG
CTTTGACTGTAGCGGGTTGGTTTTTTATGCCTACAACAAGATCCTTGAGGCTAAGCTCCCGCGCACGGCC
AATGAGATGTACCACTATCGCCGGGCAACGATTGTGGCGAACAACGACCTGCGCCGGGGAGATTTGCTGT
TTTTCCATATCCACAGCCGCGAGATAGCCGATCATATGGGCGTGTATTTGGGCGATGGGCAATTTATCGA
GTCGCCACGTACCGGCGAAACCATTCGGATAAGCCGATTAGCCGAACCTTTCTGGCAGGACCATTTTTTG
GGCGCGCGCAGGATTTTGACGGAAGAGACGATTTTGTAGGACGGATAAGGCGTTTACGCCGCATCCGGCA
GTTGTACGCAGGTGCCTGATGCGACGCTGGCGCGTCTTATCATGCCTACGAGCCCGCGAATATTTGCGAG
CCGCTTTCCCGATATAAAACAACCTCATTGCCAACCTTTCCTTTTCTTCTTACCGTTGAGAAAAAGGAGT
CGCCATGTCTGAATATCGTCGTTATTACATCAAGGGGGGAACATGGTTTTTCACGGTGAATTTACGAAAT
CGTCGAAGCCAACTTTTGACCACCCAGTACCAGATGCTCCGTCACGCCATTATTAAAGTTAAGCGAGACA
GGCCTTTTGAAATCAACGCCTGGGTCGTTTTGCCAGAGCATATGCACTGTATCTGGACATTACCTGAAGG
CGATGATGATTTTTCCTCGCGCTGGCGGGAAATTAAAAAGCAATTTACCCATGCTTGTGGATTGAAAAAT
ATCTGGCAACCACGTTTTTGGGAGCACGCCATCCGCAATACCAAAGATTATAGGCATCATGTTGATTATA
TTTATATAAATCCAGTAAAGCATGGTTGGGTAAAGCAAGTGAGTGATTGGCCATTCTCAACGTTCCATCG
CGATGTCGCGCGAGGGTTATATCCCATCGATTGGGCGGGGGACGTAACGGATTTTAGTGCCGGGGAGCGT
ATCATTTCATAACACCACCACCACCACCACTGAGATCCGGCTGCTAACAAAGCCCGAAAGGAAGCTGAGT
TGGCTGCTGCCACCGCTGAGCAATAACTAGCATAACCCCTTGGGGCCTCTAAACGGGTCTTGAGGGGTTT
TTTGCTGAAAGGAGGAACTATATCCGGATTGGCGAATGGGACGCGCCCTGTAGCGGCGCATTAAGCGCGG
CGGGTGTGGTGGTTACGCGCAGCGTGACCGCTACACTTGCCAGCGCCCTAGCGCCCGCTCCTTTCGCTTT
CTTCCCTTCCTTTCTCGCCACGTTCGCCGGCTTTCCCCGTCAAGCTCTAAATCGGGGGCTCCCTTTAGGG
TTCCGATTTAGTGCTTTACGGCACCTCGACCCCAAAAAACTTGATTAGGGTGATGGTTCACGTAGTGGGC

CATCGCCCTGATAGACGGTTTTTCGCCCTTTGACGTTGGAGTCCACGTTCTTTAATAGTGGACTCTTGTT
CCAAACTGGAACAACACTCAACCCTATCTCGGTCTATTCTTTTGATTTATAAGGGATTTTGCCGATTTCG
GCCTATTGGTTAAAAAATGAGCTGATTTAACAAAAATTTAACGCGAATTTTAACAAAATATTAACGTTTA
CAATTTCAGGTGGCACTTTTCGGGGAAATGTGCGCGGAACCCCTATTTGTTTATTTTTCTAAATACATTC
AAATATGTATCCGCTCATGAGACAATAACCCTGATAAATGCTTCAATAATATTGAAAAAGGAAGAGTATG
AGTATTCAACATTTCCGTGTCGCCCTTATTCCCTTTTTTGCGGCATTTTGCCTTCCTGTTTTTGCTCACC
CAGAAACGCTGGTGAAAGTAAAAGATGCTGAAGATCAGTTGGGTGCACGAGTGGGTTACATCGAACTGGA
TCTCAACAGCGGTAAGATCCTTGAGAGTTTTCGCCCCGAAGAACGTTTTCCAATGATGAGCACTTTTAAA
GTTCTGCTATGTGGCGCGGTATTATCCCGTATTGACGCCGGGCAAGAGCAACTCGGTCGCCGCATACACT
ATTCTCAGAATGACTTGGTTGAGTACTCACCAGTCACAGAAAAGCATCTTACGGATGGCATGACAGTAAG
AGAATTATGCAGTGCTGCCATAACCATGAGTGATAACACTGCGGCCAACTTACTTCTGACAACGATCGGA
GGACCGAAGGAGCTAACCGCTTTTTTGCACAACATGGGGGATCATGTAACTCGCCTTGATCGTTGGGAAC
CGGAGCTGAATGAAGCCATACCAAACGACGAGCGTGACACCACGATGCCTGCAGCAATGGCAACAACGTT
GCGCAAACTATTAACTGGCGAACTACTTACTCTAGCTTCCCGGCAACAATTAATAGACTGGATGGAGGCG
GATAAAGTTGCAGGACCACTTCTGCGCTCGGCCCTTCCGGCTGGCTGGTTTATTGCTGATAAATCTGGAG
CCGGTGAGCGTGGGTCTCGCGGTATCATTGCAGCACTGGGGCCAGATGGTAAGCCCTCCCGTATCGTAGT
TATCTACACGACGGGGAGTCAGGCAACTATGGATGAACGAAATAGACAGATCGCTGAGATAGGTGCCTCA
CTGATTAAGCATTGGTAACTGTCAGACCAAGTTTACTCATATATACTTTAGATTGATTTAAAACTTCATT
TTTAATTTAAAAGGATCTAGGTGAAGATCCTTTTTGATAATCTCATGACCAAAATCCCTTAACGTGAGTT
TTCGTTCCACTGAGCGTCAGACCCCGTAGAAAAGATCAAAGGATCTTCTTGAGATCCTTTTTTTCTGCGC
GTAATCTGCTGCTTGCAAACAAAAAAACCACCGCTACCAGCGGTGGTTTGTTTGCCGGATCAAGAGCTAC
CAACTCTTTTTCCGAAGGTAACTGGCTTCAGCAGAGCGCAGATACCAAATACTGTCCTTCTAGTGTAGCC
GTAGTTAGGCCACCACTTCAAGAACTCTGTAGCACCGCCTACATACCTCGCTCTGCTAATCCTGTTACCA
GTGGCTGCTGCCAGTGGCGATAAGTCGTGTCTTACCGGGTTGGACTCAAGACGATAGTTACCGGATAAGG
CGCAGCGGTCGGGCTGAACGGGGGGTTCGTGCACACAGCCCAGCTTGGAGCGAACGACCTACACCGAACT
GAGATACCTACAGCGTGAGCTATGAGAAAGCGCCACGCTTCCCGAAGGGAGAAAGGCGGACAGGTATCCG
GTAAGCGGCAGGGTCGGAACAGGAGAGCGCACGAGGGAGCTTCCAGGGGGAAACGCCTGGTATCTTTATA
GTCCTGTCGGGTTTCGCCACCTCTGACTTGAGCGTCGATTTTTGTGATGCTCGTCAGGGGGGCGGAGCCT
ATGGAAAAACGCCAGCAACGCGGCCTTTTTACGGTTCCTGGCCTTTTGCTGGCCTTTTGCTCACATGTTC
TTTCCTGCGTTATCCCCTGATTCTGTGGATAACCGTATTACCGCCTTTGAGTGAGCTGATACCGCTCGCC
GCAGCCGAACGACCGAGCGCAGCGAGTCAGTGAGCGAGGAAGCGGAAGAGCGCCTGATGCGGTATTTTCT
CCTTACGCATCTGTGCGGTATTTCACACCGCATATATGGTGCACTCTCAGTACAATCTGCTCTGATGCCG
CATAGTTAAGCCAGTATACACTCCGCTATCGCTACGTGACTGGGTCATGGCTGCGCCCCGACACCCGCCA
ACACCCGCTGACGCGCCCTGACGGGCTTGTCTGCTCCCGGCATCCGCTTACAGACAAGCTGTGACCGTCT
CCGGGAGCTGCATGTGTCAGAGGTTTTCACCGTCATCACCGAAACGCGCGAGGCAGCTGCGGTAAAGCTC
ATCAGCGTGGTCGTGAAGCGATTCACAGATGTCTGCCTGTTCATCCGCGTCCAGCTCGTTGAGTTTCTCC
AGAAGCGTTAATGTCTGGCTTCTGATAAAGCGGGCCATGTTAAGGGCGGTTTTTTCCTGTTTGGTCACTG
ATGCCTCCGTGTAAGGGGGATTTCTGTTCATGGGGGTAATGATACCGATGAAACGAGAGAGGATGCTCAC
GATACGGGTTACTGATGATGAACATGCCCGGTTACTGGAACGTTGTGAGGGTAAACAACTGGCGGTATGG
ATGCGGCGGGACCAGAGAAAAATCACTCAGGGTCAATGCCAGCGCTTCGTTAATACAGATGTAGGTGTTC
CACAGGGTAGCCAGCAGCATCCTGCGATGCAGATCCGGAACATAATGGTGCAGGGCGCTGACTTCCGCGT
TTCCAGACTTTACGAAACACGGAAACCGAAGACCATTCATGTTGTTGCTCAGGTCGCAGACGTTTTGCAG
CAGCAGTCGCTTCACGTTCGCTCGCGTATCGGTGATTCATTCTGCTAACCAGTAAGGCAACCCCGCCAGC
CTAGCCGGGTCCTCAACGACAGGAGCACGATCATGCGCACCCGTGGGGCCGCCATGCCGGCGATAATGGC
CTGCTTCTCGCCGAAACGTTTGGTGGCGGGACCAGTGACGAAGGCTTGAGCGAGGGCGTGCAAGATTCCG
AATACCGCAAGCGACAGGCCGATCATCGTCGCGCTCCAGCGAAAGCGGTCCTCGCCGAAAATGACCCAGA
GCGCTGCCGGCACCTGTCCTACGAGTTGCATGATAAAGAAGACAGTCATAAGTGCGGCGACGATAGTCAT
GCCCCGCGCCCACCGGAAGGAGCTGACTGGGTTGAAGGCTCTCAAGGGCATCGGTCGAGATCCCGGTGCC
TAATGAGTGAGCTAACTTACATTAATTGCGTTGCGCTCACTGCCCGCTTTCCAGTCGGGAAACCTGTCGT
GCCAGCTGCATTAATGAATCGGCCAACGCGCGGGGAGAGGCGGTTTGCGTATTGGGCGCCAGGGTGGTTT
TTCTTTTCACCAGTGAGACGGGCAACAGCTGATTGCCCTTCACCGCCTGGCCCTGAGAGAGTTGCAGCAA
GCGGTCCACGCTGGTTTGCCCCAGCAGGCGAAAATCCTGTTTGATGGTGGTTAACGGCGGGATATAACAT

GAGCTGTCTTCGGTATCGTCGTATCCCACTACCGAGATATCCGCACCAACGCGCAGCCCGGACTCGGTAA
TGGCGCGCATTGCGCCCAGCGCCATCTGATCGTTGGCAACCAGCATCGCAGTGGGAACGATGCCCTCATT
CAGCATTTGCATGGTTTGTTGAAAACCGGACATGGCACTCCAGTCGCCTTCCCGTTCCGCTATCGGCTGA
ATTTGATTGCGAGTGAGATATTTATGCCAGCCAGCCAGACGCAGACGCGCCGAGACAGAACTTAATGGGC
CCGCTAACAGCGCGATTTGCTGGTGACCCAATGCGACCAGATGCTCCACGCCCAGTCGCGTACCGTCTTC
ATGGGAGAAAATAATACTGTTGATGGGTGTCTGGTCAGAGACATCAAGAAATAACGCCGGAACATTAGTG
CAGGCAGCTTCCACAGCAATGGCATCCTGGTCATCCAGCGGATAGTTAATGATCAGCCCACTGACGCGTT
GCGCGAGAAGATTGTGCACCGCCGCTTTACAGGCTTCGA**CGCCGCTTCG**

Fragment 2 (6kb)

TTCTACCATCGACACCACCACGCTGGCACCCAGTTGATCGGCGCGAGATTTAATCGCCGCGACAATTTGC
GACGGCGCGTGCAGGGCCAGACTGGAGGTGGCAACGCCAATCAGCAACGACTGTTTGCCCGCCAGTTGTT
GTGCCACGCGGTTGGGAATGTAATTCAGCTCCGCCATCGCCGCTTCCACTTTTTCCCGCGTTTTCGCAGA
AACGTGGCTGGCCTGGTTCACCACGCGGGAAACGGTCTGATAAGAGACACCGGCATACTCTGCGACATCG
TATAACGTTACTGGTTTCACATTCACCACCCTGAATTGACTCTCTTCCGGGCGCTATCATGCCATACCGC
GAAAGGTTTTGCGCCATTCGATGGTGTCCGGGATCTCGACGCTCTCCCTTATGCGACTCCTGCATTAGGA
AGCAGCCCAGTAGTAGGTTGAGGCCGTTGAGCACCGCCGCCGCAAGGAATGGTGCATGCAAGGAGATGGC
GCCCAACAGTCCCCCGGCCACGGGGCCTGCCACCATACCCACGCCGAAACAAGCGCTCATGAGCCCGAAG
TGGCGAGCCCGATCTTCCCCATCGGTGATGTCGGCGATATAGGCGCCAGCAACCGCACCTGTGGCGCCGG
TGATGCCGGCCACGATGCGTCCGGCGTAGAGGATCGAGATCTCGATCCCGCGAAATTAATACGACTCACT
ATAGGGGAATTGTGAGCGGATAACAATTCCCCTCTAGAAATAATTTTGTTTAACTTTAAGAAGGAGATAT
ACATATGAAAAAGTGGTTATTAGCTGCAGGTCTCGGTTTAGCACTGGCAACTTCTGCTCAGGCGGCTGAC
AAAATTGCAATCGTCAACATGGGCAGCCTGTTCCAGCAGGTAGCGCAGAAAACCGGTGTTTCTAACACGC
TGGAAAATGAGTTCAAAGGCCGTGCCAGCGAACTGCAGCGTATGGAAACCGATCTGCAGGCTAAAATGAA
AAAGCTGCAGTCCATGAAAGCGGGCAGCGATCGCACTAAGCTGGAAAAAGACGTGATGGCTCAGCGCCAG
ACTTTTGCTCAGAAAGCGCAGGCTTTTGAGCAGGATCGCGCACGTCGTTCCAACGAAGAACGCGGCAAAC
TGGTTACTCGTATCCAGACTGCTGTGAAATCCGTTGCCAACAGCCAGGATATCGATCTGGTTGTTGATGC
AAACGCCGTTGCTTACAACAGCAGCGATGTAAAAGACATCACTGCCGACGTACTGAAACAGGTTAAATAA
GTAATGCCTTCAATTCGACTGGCTGATTTAGCGCAGCAGTTGGATGCAGAACTACACGGTGATGGCGATA
TCGTCATCACCGGCGTTGCGTCCATGCAATCTGCACAAACAGGTCACATTACGTTCATGGTTAACCCAAA
ATACCGTGAGCATTTAGGCTTGTGCCAGGCGTCCGCGGTTGTCATGACCCAGGACGATCTTCCTTTCGCG
AAAAGTGCCGCACTGGTAGTGAAGAATCCCTACCTGACTTACGCGCGCATGGCGCAAATTTTAGATACCA
CGCCGCAGCCCGCGCAGAACATTGCACCCAGTGCGGTGATCGACGCGACGGCGAAGCTGGGTAACAACGT
ATCGATTGGCGCTAACGCGGTGATTGAGTCCGGCGTTGAACTGGGCGATAACGTGATTATCGGTGCCGGT
TGCTTCGTAGGTAAAAACAGCAAAATCGGTGCAGGTTCGCGTCTCTGGGCGAACGTAACCATTTACCATG
AGATCCAGATCGGTCAGAATTGCCTGATCCAGTCCGGAACAGTGGTAGGCGCAGACGGCTTTGGTTATGC
CAACGATCGTGGTAACTGGGTGAAGATCCCACAGATTGGTCGCGTAATTATTGGCGATCGCGTGGAGATC
GGTGCCTGCACAACCATCGATCGCGGCGCGCTGGATGACACTATTATTGGCAATGGCGTGATCATTGATA
ACCAGTGCCAGATTGCACATAACGTCGTGATTGGCGACAATACGGCGGTTGCCGGTGGCGTCATTATGGC
GGGCAGCCTGAAAATTGGTCGTTACTGCATGATCGGCGGAGCCAGCGTAATCAACGGGCATATGGAAATA
TGCGACAAAGTGACGGTTACGGGCATGGGTATGGTGATGCGTCCCATCACTGAACCAGGCGTCTATTCCT
CAGGCATTCCGCTGCAACCCAACAAAGTCTGGCGCAAAACCGCTGCACTGGTGATGAACATTGATGACAT
GAGCAAGCGTCTGAAATCGCTTGAGCGCAAGGTTAATCAACAAGACTAACGTTCCATCTTTTGTTCGCCA
AACTTTACGGCCTGTCTCATTCTTACGATTGCGGCAGGCCGTGTTATTATTGTCGTTTCTTATATTTTGA
CAGGAAGAGTATCTTGACTACTAACACTCATACTCTGCAGATTGAAGAGATTTTAGAACTTCTGCCGCAC
CGTTTCCCGTTCTTACTGGTGGATCGCGTGCTGGATTTTGAAGAAGGTCGTTTTCTGCGCGCAGTAAAAA
ATGTCTCTGTCAATGAGCCATTCTTCCAGGGCCATTTCCCTGGAAAACCGATTTTCCCGGGTGTGCTGAT
TCTGGAAGCAATGGCACAGGCAACAGGTATTCTGGCGTTTAAAAGCGTAGGAAAACTGGAACCGGGTGAG
CTGTACTACTTCGCTGGTATTGACGAAGCGCGCTTCAAGCGCCCGGTCGTGCCTGGCGATCAAATGATCA
TGGAAGTCACTTTCGAAAAAACGCGCCGCGGCCTGACCCGTTTTAAAGGGGTTGCTCTGGTCGATGGTAA
AGTAGTTTGCGAAGCAACGATGATGTGTGCTCGTAGCCGGGAGGCCTGATACGTGATTGATAAATCCGCC

TTTGTGCATCCAACCGCCATTGTGGAAGAGGGCGCGTCAATTGGCGCGAACGCACACATTGGTCCTTTTT
GTATCGTTGGACCCCATGTCGAAATTGGTGAGGGTACCGTACTGAAATCTCACGTTGTCGTGAATGGTCA
TACTAAAATTGGCCGCGATAATGAGATTTATCAGTTCGCCTCCATCGGCGAAGTTAACCAGGATCTGAAA
TATGCTGGCGAACCGACCCGTGTGGAAATCGGCGATCGTAACCGCATTCGCGAAAGCGTCACCATTCATC
GTGGCACAGTCCAGGGCGGTGGATTGACGAAGGTGGGCAGCGACAACTTACTGATGATCAACGCGCACAT
TGCGCACGATTGTACGGTAGGTAACCGCTGTATTCTCGCCAACAACGCAACGCTGGCGGGTCACGTATCG
GTTGACGACTTCGCGATCATCGGCGGCATGACCGCAGTCCATCAGTTCTGCATCATTGGTGCGCACGTGA
TGGTTGGCGGCTGCTCCGGTGTGGCGCAGGACGTCCCTCCTTATGTCATTGCGCAGGGTAACCACGCAAC
GCCGTTCGGTGTCAATATCGAAGGGCTGAAGCGCCGCGGATTCAGCCGTGAGGCGATTACCGCTATCCGC
AATGCGTATAAGCTGATTTATCGTAGCGGTAAAACGCTCGATGAAGTGAAACCGGAAATTGCTGAACTGG
CGGAAACATATCCGGAAGTGAAAGCCTTTACCGATTTCTTTGCACGCTCAACGCGCGGTCTGATTCGTTA
ATGACTGAACAGCGTCCATTAACGATTGCCCTGGTCGCCGGAGAAACCTCCGGCGATATCCTGGGGGCCG
GTTTAATCCGCGCTCTGAAAGAACATGTGCCCAACGCCCGCTTTGTTGGTGTTGCCGGGCCACGAATGCA
GGCTGAAGGCTGCGAAGCCTGGTACGAAATGGAAGAACTGGCGGTGATGGGCATTGTTGAAGTGCTCGGT
CGTCTGCGTCGCTTACTGCATATTCGTGCCGATCTGACAAAGCGTTTTGGCGAACTGAAGCCAGATGTTT
TTGTTGGTATTGATGCGCCTGACTTCAATATTACTCTTGAAGGTAACCTCAAAAAGCAGGGTATCAAAAC
CATTCATTACGTCAGTCCGTCAGTCTGGGCGTGGCGACAGAAACGTGTTTTCAAAATAGGCAGAGCCACC
GATC
TGGTGCTCGCATTTCTGCCTTTCGAAAAAGCGTTTTATGACAAATACAACGTACCGTGCCGCTTTATCGG
TCATACCATGGCTGATGCCATGCCATTAGATCCAGATAAAAATGCCGCCCGTGATGTGCTGGGGATCCCT
CACGATGCCCACTGCCTGGCGTTGCTACCGGGGAGCCGTGGTGCAGAAGTTGAAATGCTTAGTGCCGATT
TCCTGAAAACGGCCCAGCTTTTGCGCCAGACATATCCGGATCTCGAAATCGTGGTGCCACTGGTGAATGC
CAAACGCCGCGAGCAGTTTGAACGCATCAAAGCTGAAGTCGCGCCAGACCTTTCAGTTCATTTGCTGGAT
GGGATGGGCCGTGAGGCGATGGTCGCCAGCGATGCGGCGCTACTGGCGTCGGGTACGGCAGCCCTGGAGT
GTATGCTGGCGAAATGCCCGATGGTGGTGGGATATCGCATGAAGCCTTTTACCTTCTGGTTGGCGAAGCG
GCTGGTGAAAACTGATTATGTCTCGCTGCCAAATCTGCTGGCGGGCAGAGAGTTAGTCAAAGAATTATTG
CAGGAAGAGTGTGAGCCGCAAAAACTGGCTGCGGCGCTGTTACCGCTGTTGGCGAACGGGAAAACCAGCC
ACGCGATGCACGATACCTTCCGTGAACTGCATCAGCAGATCCGCTGCAATGCCGATGAGCAGGCGGCACA
AGCCGTTCTGGAGTTAGCACAATGATCGAATTTGTTTATCCGCACACGCAGCTGGTTGCGGGTGTGGATG
AAGTCGGACGCGGGCCGTTAGTTGGCGCGGTCGTCACCGCTGCGGTGATCCTTGACCCGGCGCGCCCGAT
TGCCGGGCTGAATGATTCCAAAAAGCTGAGCGAAAAACGCCGTCTGGCGCTCTATGAAGAGATCAAAGAG
AAAGCGTTGAGCTGGAGTCTGGGCCGCGCGGAACCCCACGAAATCGACGAGCTGAACATTCTTCATGCGA
CCATGCTGGCGATGCAGCGTGCCGTCGCTGGGCTGCATATTGCGCCGGAATATGTGTTGATTGATGGTAA
CCGCTGCCCGAAATTACCGATGCCTGCGATGGCTGTGGTGAAAGGCGATAGCCGCGTACCGGAAATCAGT
GCCGCGTCTATCCTGGCGAAAGTGACGCGTGACGCCGAAATGGCGGCGCTGGATATTGTTTTCCCGCAAT
ATGGTTTTGCCCAACACAAAGGGTACCCAACCGCTTTTCATCTGGAAAAACTGGCTGAACACGGCGCGAC
CGAACACCATCGGCGCAGCTTTGGGCCTGTCAAACGCGCACTGGGACTTGCGTCCTGATTCTTGTGTCGA
GATTAAGTAAACCGGAATCTGAAGATGTCTGAACCACGTTTCGTACACCTGCGGGTGCACAGCGACTACT
CGATGATCGATGGCCTGGCCAAAACCGCACCGTTGGTAAAAAAGGCGGCGGCGTTGGGTATGCCAGCACT
GGCGATCACCGATTTCACCAACCTTTGTGGTCTGGTGAAGTTCTACGGAGCGGGACATGGCGCAGGGATT
AAGCCTATCGTCGGGGCAGATTTTAACGTCCAGTGCGACCTGCTGGGTGATGAGTTAACCCACCTGACGG
TACTGGCGGCGAACAATACCGGCTATCAGAATCTGACGTTGCTGATCTCAAAAGCGTATCAGCGCGGGTA
CGGTGCCGCCGGGCCGATCATCGATCGCGACTGGCTTATCGAATTAAACGAAGGGTTGATCCTTCTTTCC
GGCGGACGCATGGGCGACGTCGGACGCAGTCTTTTGCGTGGTAACAGCGCGCTGGTAGATGAGTGTGTCG
CGTTTTATGAAGAACACTTCCCGGATCGCTATTTTCTCGAGCTGATCCGCACCGGCAGGCCGGATGAAGA
AAGCTATCTGCACGCGGCGGTGGAACTGGCGGAAGCGCGCGGTTTGCCCGTCGTGGCGACCAACGACGTG
CGCTTTATCGACAGCAGCGACTTT**GACGCACAC**

Fragment 3 (4kb)

GAAATCCGCGTCGCGATCCACGACGGCTTTACCCTCGACGATCCTAAACGCCCGCGTAACTATTCGCCGC
AGCAATATATGCGTAGCGAAGAGGAGATGTGTGAGCTGTTTGCCGACATCCCCGAAGCCCTTGCCAACAC
CGTTGAGATCGCCAAACGCTGTAACGTAACCGTGCGTCTTGGTGAATACTTCCTGCCGCAGTTCCCGACC
GGGGACATGAGCACCGAAGATTATCTGGTCAAGCGTGCAAAAGAGGGCCTGGAAGAGCGTCTGGCCTTTT
TATTCCCTGATGAGGAAGAACGTCTTAAGCGCCGCCCGGAATATGACGAACGTCTGGAGACTGAACTTCA
GGTTATCAACCAGATGGGCTTCCCGGGCTACTTCCTCATCGTTATGGAATTTATCCAGTGGTCGAAAGAT
AACGGCGTACCGGTAGGGCCAGGCCGTGGCTCCGGTGCGGGTTCACTGGTGGCCTACGCGCTGAAAATCA
CCGACCTCGATCCGCTGGAATTTGACCTGCTGTTCGAACGTTTCCTTAACCCGGAACGTGTCTCCATGCC
TGACTTCGACGTTGACTTCTGTATGGAGAAACGCGATCAGGTTATCGAGCACGTAGCGGACATGTACGGT
CGTGATGCGGTATCGCAGATCATCACCTTCGGTACAATGGCGGCGAAAGCGGTGATCCGCGACGTAGGCC
GCGTGCTGGGGCATCCGTACGGCTTTGTCGATCGTATCTCGAAACTGATCCCGCCCGATCCGGGGATGAC
GCTGGCGAAAGCGTTTGAAGCCGAGCCGCAGCTGCCGGAAATCTACGAAGCGGATGAAGAAGTTAAGGCG
CTGATCGACATGGCGCGCAAACTGGAAGGGGTCACCCGTAACGCCGGTAAGCACGCCGGTGGGGTGGTTA
TCGCGCCGACCAAAATTACCGATTTTGCGCCGCTTTACTGCGATGAAGAGGGCAAACATCCGGTCACCCA
GTTTGATAAAAGCGACGTTGAATACGCCGGACTGGTGAAGTTCGACTTCCTTGGTTTGCGTACGCTCACC
ATCATCAACTGGGCGCTGGAGATGATCAACAAGCGGCGGGCGAAGAATGGCGAGCCGCCGCTGGATATCG
CTGCGATCCCGCTGGATGATAAGAAAAGCTTCGACATGCTGCAACGCTCGGAAACCACGGCGGTATTCCA
GCTTGAATCGCGCGGCATGAAGGACCTGATCAAGCGTCTACAACCTGACTGCTTCGAAGATATGATCGCC
CTAGTGGCACTGTTCCGCCCCGGTCCGTTGCAATCAGGGATGGTGGATAACTTTATCGACCGTAAACATG
GTCGTGAAGAGATCTCCTATCCGGACGTACAGTGGCAGCATGAAAGCCTGAAACCGGTACTGGAGCCAAC
CTACGGCATTATCCTGTATCAGGAACAGGTCATGCAGATTGCGCAGGTGCTTTCTGGTTATACCCTCGGT
GGCGCGGATATGCTGCGTCGTGCGATGGGTAAGAAAAAGCCGGAAGAGATGGCTAAGCAACGTTCTGTAT
TTGCTGAAGGTGCAGAAAAGAACGGAATCAACGCTGAACTGGCGATGAAAATCTTCGACCTGGTGGAGAA
ATTCGCTGGTTACGGATTTAACAAATCGCACTCTGCGGCCTATGCTTTGGTGTCATATCAAACGTTATGG
CTGAAAGCGCACTATCCTGCGGAGTTTATGGCGGCGGTAATGACCGCCGATATGGACAACACCGAGAAGG
TGGTGGGTCTGGTGGATGAGTGCTGGCGGATGGGGCTGAAAATCCTGCCACCAGATATAAACTCCGGTCT
TTACCATTTCCACGTCAACGACGACGGCGAAATCGTGTATGGTATTGGCGCGATCAAAGGGGTCGGTGAA
GGTCCGATTGAGGCCATCATCGAAGCCCGTAATAAAGGCGGCTACTTCCGCGAACTGTTTGATCTCTGCG
CCCGTACCGACACCAAAAAGTTGAACCGTCGCGTGCTGGAAAAACTGATCATGTCCGGGGCGTTTGACCG
TCTTGGGCCACATCGCGCAGCGCTGATGAACTCGCTGGGCGATGCGTTAAAAGCGGCAGATCAACACGCG
AAAGCGGAAGCTATCGGTCAGGCCGATATGTTCGGCGTGCTGGCCGAAGAGCCGGAACAAATTGAACAAT
CCTACGCCAGCTGCCAACCGTGGCCGGAGCAGGTGGTATTAGATGGGGAACGTGAAACGTTAGGCCTGTA
CCTGACCGGACACCCTATCAACCAGTATTTAAAAGAGATTGAGCGTTATGTCGGAGGCGTAAGGCTGAAA
GACATGCACCCGACAGAACGTGGTAAAGTCATCACGGCTGCGGGGCTCGTTGTTGCCGCGCGGGTTATGG
TCACCAAGCGCGGCAATCGTATCGGTATCTGCACGCTGGATGACCGTTCCGGGCGGCTGGAAGTGATGTT
GTTTACTGACGCCCTGGATAAATACCAGCAATTGCTGGAAAAAGACCGCATACTTATCGTCAGCGGACAG
GTCAGCTTTGATGACTTCAGCGGTGGGCTTAAAATGACCGCTCGCGAAGTGATGGATATTGACGAAGCCC
GGGAAAAATATGCTCGCGGGCTTGCTATCTCGCTGACGGACAGGCAAATTGATGACCAGCTTTTAAACCG
ACTCCGTCAGTCTCTGGAACCCCACCGCTCTGGGACAATTCCAGTACATCTCTACTATCAGAGGGCGGAT
GCACGCGCGCGGTTGCGTTTTGGCGCGACGTGGCGTGTCTCTCCGAGCGATCGTTTATTAAACGATCTCC
GTGGCCTCATTGGTTCGGAGCAGGTGGAACTGGAGTTTGACTAATACAGGAATACTATGAGTCTGAATTT
CCTTGATTTTGAACAGCCGATTGCAGAGCTGGAAGCGAAAATCGATTCTCTGACTGCGGTTAGCCGTCAG
GATGAGAAACTGGATATTAACATCGATGAAGAAGTGCATCGTCTGCGTGAAAAAAGCGTAGAACTGACAC
GTAAAATCTTCGCCGATCTCGGTGCATGGCAGATTGCGCAACTGGCACGCCATCCACAGCGTCCTTATAC
CCTGGATTACGTTCGCCTGGCATTTGATGAATTTGACGAACTGGCTGGCGACCGCGCGTATGCAGACGAT
AAAGCTATCGTCGGTGGTATCGCCCGTCTCGATGGTCGTCCGGTGATGATCATTGGTCATCAAAAAGGTC
GTGAAACCAAAGAAAAAATTCGCCGTAACTTTGGTATGCCAGCGCCAGAAGGTTACCGCAAAGCACTGCG
TCTGATGCAAATGGCTGAACGCTTTAAGATGCCTATCATCACCTTTATCGACACCCCGGGGGCTTATCCT
GGCGTGGGCGCAGAAGAGCGTGGTCAGTCTGAAGCCATTGCACGCAACCTGCGTGAAATGTCTCGCCTCG
GCGTACCGGTAGTTTGTACGGTTATCGGTGAAGGTGGTTCTGGCGGTGCGCTGGCGATTGGCGTGGGCGA
TAAAGTGAATATGCTGCAATACAGCACCTATTCCGTTATCTCGCCGGAAGGTTGTGCGTCCATTCTGTGG

AAGAGCGCCGACAAAGCGCCGCTGGCGGCTGAAGCGATGGGTATCATTGCTCCGCGTCTGAAAGAACTGA
AACTGATCGACTCCATCATCCCGGAACCACTGGGTGG
TGCTCACCGTAACCCGGAAGCGATGGCGGCATCGTTGAAAGCGCAACTGCTGGCGGATCTGGCCGATCTC
GACGTGTTAAGCACTGAAGATTTAAAAAATCGTCGTTATCAGCGCCTGATGAGCTACGGTTACGCGTAAT
TCGCAAAAGTTCTGAAAAAGGGTCACTTCGGTGGCCCTTTTTTATCGCCACGGTTTGAGCAGGCTATGAT
TAAGGAAGGATTTTCCAGGAGGAACACATGAACATCATTGCCATTATGGGACCGCATGGCGTCTTTTATA
AAGATGAGCCCATCAAAGAACTGGAGTCGGCGCTGGTGGCG**CAAGGCTTT**

Fragment 4 (5kb)

GCGATTATCTGGCCACAAAACAGCGTTGATTTGCTGAAATTTATCGAGCATAACCCTCGAATTTGCGGCG
TGATTTTTGACTGGGATGAGTACAGTCTCGATTTATGTAGCGATATCAATCAGCTTAATGAATATCTCCC
GCTTTATGCCTTCATCAACACCCACTCGACGATGGATGTCAGCGTGCAGGATATGCGGATGGCGCTCTGG
TTTTTTGAATATGCGCTGGGGCAGGCGGAAGATATCGCCATTCGTATGCGTCAGTACACCGACGAATATC
TTGATAACATTACACCGCCGTTCACGAAAGCCTTGTTTACCTACGTCAAAGAGCGGAAGTACACCTTTTG
TACGCCGGGGCATATGGGCGGCACCGCATATCAAAAAAGCCCGGTTGGCTGTCTGTTTTATGATTTTTTC
GGCGGGAATACTCTTAAGGCTGATGTCTCTATTTCGGTCACCGAGCTTGGTTCGTTGCTCGACCACACCG
GGCCACACCTGGAAGCGGAAGAGTACATCGCGCGGACTTTTGGCGCGGAACAGAGTTATATCGTTACCAA
CGGAACATCGACGTCGAACAAAATTGTGGGTATGTACGCCGCGCCATCCGGCAGTACGCTGTTGATCGAC
CGCAATTGTCATAAATCGCTGGCGCATCTGTTGATGATGAACGATGTAGTGCCAGTCTGGCTGAAACCGA
CGCGTAATGCGTTGGGGATTCTTGGTGGGATCCCGCGCCGTGAATTTACTCGCGACAGCATCGAAGAGAA
AGTCGCTGCTACCCACGCAAGCACAATGGCCGGTTCATGCGGTGATCACCAACTCCACCTATGATGGCTTG
CTCTACAACACCGACTGGATCAAACAGACGCTGGATGTCCCGTCGATTCACTTCGATTCTGCCTGGGTGC
CGTACACCCATTTTCATCCGATCTACCAGGGTAAAAGTGGTATGAGCGGCGAGCGTGTTGCGGGAAAAGT
GATCTTCGAAACGCAATCGACCCACAAAATGCTGGCGGCGTTATCGCAGGCTTCGCTGATCCACATTAAA
GGCGAGTATGACGAAGAGGCCTTTAACGAAGCCTTTATGATGCATACCACCACCTCGCCCAGTTATCCCA
TTGTTGCTTCGGTTGAGACGGCGGCGGCGATGCTGCGTGGTAATCCGGGCAAACGGCTGATTAACCGTTC
AGTAGAACGAGCTCTGCATTTTCGCAAAGAGGTCCAGCGGCTGCGGGAAGAGTCTGACGGTTGGTTTTTC
GATATCTGGCAACCGCCGCAGGTGGATGAAGCCGAATGCTGGCCCGTTGCGCCTGGCGAACAGTGGCACG
GCTTTAACGATGCGGATGCCGATCATATGTTTCTCGATCCGGTTAAAGTCACTATTTTGACACCGGGGAT
GGACGAGCAGGGCAATATGAGCGAGGAGGGGATCCCGGCGGCGCTGGTAGCAAAATTCCTCGACGAACGT
GGGATCGTAGTAGAGAAAACCGGCCCTTATAACCTGCTGTTTCTCTTTAGTATTGGCATCGATAAAACCA
AAGCAATGGGATTATTGCGTGGGTTGACGGAATTCAAACGCTCTTACGATCTCAACCTGCGGATCAAAAA
TATGCTACCCGATCTCTATGCAGAAGATCCCGATTTCTACCGCAATATGCGTATTCAGGATCTGGCACAA
GGGATCCATAAGCTGATTCGTAAACACGATCTTCCCGGTTTGATGTTGCGGGCATTCGATACTTTGCCGG
AGATGATCATGACGCCACATCAGGCATGGCAACGACAAATTAAAGGCGAAGTAGAAACCATTGCGCTGGA
ACAACTGGTCGGTAGAGTATCGGCAAATATGATCCTGCCTTATCCACCGGGCGTACCGCTGTTGATGCCT
GGAGAAATGCTGACCAAAGAGAGCCGCACAGTACTCGATTTTCTACTGATGCTTTGTTCCGTCGGGCAAC
ATTACCCCGGTTTTGAAACGGATATTCACGGCGCGAAACAGGACGAAGACGGCGTTTACCGCGTACGAGT
CCTAAAAATGGCGGGATAACTTGCCAGAGCGGCTTCCGGGCGAGTAACGTGCTGTTAACAAATAAAGGAG
ACGTTATGCTGGGTTTAAAACAGGTTCACCATATTGCGATTATTGCGACGGATTATGCGGTGAGCAAAGC
TTTCTACTGCGATATTCTTGGTTTCACGCTGCAAAGCGAAGTCTATCGCGAAGCGCGCGACTCATGGAAA
GGGGATTTGGCGCTTAATGGGCAATATGTGATTGAGCTTTTCTCATTTCCGTTCCCGCCGGAACGACCCA
GCCGACCGGAAGCTTGCGGTCTGCGTCATCTGGCTTTTAGCGTTGATGACATCGATGCGGCAGTGGCGCA
CCTTGAAAGCCATAACGTGAAGTGTGAAACCATCCGTGTCGATCCATACACGCAAAAACGCTTCACCTTC
TTTAACGATCCGGACGGGCTGCCGTTGGAACTGTATGAGCAGTAAGGCTTGTCATCGCCGCATTTGCCCG
GTAACGTGCCGGGCATTGCTACTGTAAAATCGCACCATCATGACACTCACGCTCAATAGACAACTTCTCA
CCTCACGCCAGATTCTGGTGGCCTTTAGCGGCGGGCTTGACTCCACCGTTCTGCTGCATCAGTTGGTGCA
GTGGCGGACGGAAAATCCGGGTGTCGCTCTGCGCGCTATTCATGTGCATCACGGTTTAAGTGCCAATGCC
GATGCCTGGGTTACGCATTGCGAAAACGTCTGCCAACAGTGGCAGGTGCCGCTGGTGGTCGAACGCGTAC
AACTTGCGCAAGAAGGACTGGGCATTGAGGCCCAGGCGCGGCAGGCACGTTATCAGGCATTTGCCCGCAC
CTTGTTGCCCGGTGAAGTGCTGGTCACCGCGCAACATCTCGACGATCAATGTGAAACCTTTCTGCTGGCG

CTAAAACGCGGCAGTGGCCCTGCCGGGCTTTCGGCTATGGCGGAAGTCTCGGAGTTTGCCGGAACGCGGC
TTATTCGCCCGTTGCTCGCCCGCACGCGGGGGGAACTGGTGCAGTGGGCGCGTCAGTATGATTTACGCTG
GATTGAAGACGAAAGTAATCAGGACGACTCATACGATCGTAACTTTCTGCGCCTGCGCGTAGTGCCGTTA
TTGCAGCAGCGTTGGCCGCATTTTGCCGAAGCAACGGCCCGCAGCGCCGCACTTTGTGCTGAACAAGAGA
GCCTGCTGGATGAACTGCTGGCAGATGATTTAGCACACTGTCAATCGCCGCAGGGGACGCTGCAGATTGT
GCCAATGCTGGCGATGAGTGATGCCCGCCGCGCGGCGATTATCCGCCGCTGGCTGGCAGGGCAGAATGCA
CCGATGCCTTCCCGCGACGCGTTGGTGAGGATCTGGCAGGAAGTGGCGCTGGCGCGGGAAGATGCCTCAC
CCTGTTTACGTTTGGGCGCGTTTGAAATCCGACGCTATCAGTCGCAACTGTGGTGGATTAAATCCGTCAC
CGGGCAAAGCGAAAACATTGTGCCGTGGCAGACGTGGCTTCAACCGCTGGAATTACCGGCGGGGCTGGGA
AGTGTACAGCTTAATGCGGGAGGCGATATTCGCCCTCCGCGTGCAGACGAAGCGGTCAGCGTGCGTTTCA
AAGCGCCAGGATTGCTGCATATTGTCGGGCGTAACGGCGGACGTAAGCTAAAGAAAATCTGGCAAGAGCT
GGGCGTGCCGCCGTGGCTACGTGACACCACGCCACTGCTGTTTTATGGCGAAACGCTGATTGCGGCGGCA
GGGGTATTTGTGACGCAAGAAGGTGTGGCTGAAGGTGAGAATGGCGTAAGTTTTGTCTGGCAGAAAACGC
TTAGTTAAGTGAAAGCCGGATAAGACGCATCAAACGTCGCATCCGGCGAAAGTCAATCAGGATTCGCTTA
CCACCACCGTACCGATTTCCGGGTGGCTAAAGCTGGTAATTTTATCCAGACGCAGCTCGCGGGTTTCGCC
AGCGGCCTCGACGACCAGGTACTCCACATTTTTGCGGGAGACTAAATCACTGGCTTTTGCCTGCAATTTT
TCGCCATCTTTCAGCTCAAGTGTCAGCATTAAATGATGCTGGCAGGCGAGCTCAAGATTATCGTAATCAT
CACAATTGATTGGTTGATACGTATCATTCATTGACATAATCGCTCACCAGTAAGTTTGCCGCAGCGTATG
CTGCTTTTTCCCTGACAGCCTCAGAAAGGGCGTCGTCGGCAGCCATTTCATTCAGCACTTTCAAAACGCA
GCCCAGCGCGTCCGGAACGTATCCTAAGTCTCCGCTGGCGATTTCCGCGTACCGCTTGCGTATTAACTCA
CAATATTTTTCCACATGCCCTCCTGTCAGCACTCTGACTTAACCGTGGATGCAAGTCTAAGCCTACGAAG
ATAAACTCTGTTTCGCAAGGTGACTATACCACACTCATTTCTGCAATATCAGCGCCGCAACTGCACGTAT
TCCGTTACAATGGCCTCCTGATTCGAAAGGAGTTTTCTTATGGCGCTTAAAGCGACAATTTATAAAGCGA
CGGTTAATGTGGCCGATCTCGACCGCAACCAGTTTCTCGATGCCTCTCTGACGCTGGCGCGCCATCCTTC
AGAAACCCAGGAGCGTATGATGCTGCGCTTGCTGGCGTGGCTGAAATATGCTGATGAACGTCTGCAATTT
ACCCGTGGTTTGTGTGCCGATGATGAGCCGGAAGCGTGGCTGCGTAACGATCACCTGGGCATTGATTTGT
GGATTGAGCTGGGGCTGCCGGATGAGCGGCGGATTAAGAAAGCCTGCACCCAGGCCGCAGAAGTGGCGCT
GTTTACCTATAATAGTCGGGCGGCGCAAATCTGGTGGCAGCAAAATCAGAGCAAATGTGTGCAGTTTGCC
AATCTTTCCGTCTGGTATCTGGACGATGAACAACTGGCGAAAGTAAGCGCCTTTGCCGATCGTACCATGA
CGCTGCAGGCAACGATTCAGGATGGC**GTGATCTGG**

Fragment 5 (5.5kb)

TTATCGGATGATAAGAATAATCTGGAAGTGAACTTAACCGCCTGGCAACAACCTTCATGATTGTGATTTC
CCGACATGTTGCTATTCCCGATGGTGAGCTTGAGATCACCGCCATTCGTGCGCAGGGCGCGGGCGGGCAG
CATGTTAATAAGACCTCAACGGCTATTCATCTGCGTTTTGACATTCGGGCGTCCAGCCTGCCAGAGTATT
ACAAAGAGCGTCTGCTCGCCGCCAGCCATCATTTGATCAGCAGTGATGGCGTGATTGTCATTAAGGCACA
GGAATACCGCAGTCAGGAACTGAACCGCGAAGCAGCTCTGGCCCGGCTGGTGGCTATGATTAAAGAATTA
ACAACAGAAAAAAAAGCCCGACGACCCACGCGGCCCACCCGTGCATCGAAAGAGCGCAGGCTGGCATCGA
AAGCACAAAAATCAAGCGTGAAGGCGATGCGCGGCAAAGTGCGCAGCGGTCGGGAATAAAAAGAAGGAAT
GGATGGTGAAAAAAGCGATAGTGACAGCGATGGCTGTAATCAGCCTCTTTACTCTGATGGGATGTAATAA
TCGGGCCGAAGTCGATACGCTTTCTCCGGCGCAGGCTGCCGAACTGAAACCGATGCCGCAAAGTTGGCGC
GGCGTGCTGCCGTGTGCCGATTGCGAAGGAATCGAAACCTCTCTGTTCCTCGAAAAAGACGGAACATGGG
TGATGAATGAGCGTTATCTCGGTGCTCGTGAAGAACCTTCCTCCTTCGCTTCCTACGGTACATGGGCGCG
AACCGCTGACAAGCTGGTATTAACCGACAGCAAAGGTGAAAAGTCATATTATCGGGCGAAAGGCGATGCG
CTGGAGATGCTCGATCGTGAAGGCAATCCGATTGAATCGCAGTTCAACTATACGCTGGAAGCGGCACAAT
CCAGTTTACCTATGACGCCGATGACCCTGCGGGGCATGTATTTTTATATGGCTGATGCGGCGACCTTCAC
TGATTGCGCGACCGGAAAACGTTTCATGGTAGCGAATAACGCAGAGCTGGAGCGTAGCTACCTGGCTGCG
CGCGGTCACAGTGAAAAACCGGTGTTACTGTCAGTAGAAGGTCACTTTACGCTTGAGGGTAATCCGGATA
CCGGTGCGCCGACTAAAGTATTGGCACCCGATACGGCAGGGAAATTTTACCCCAACCAGGATTGCAGTAG
TTTGGGGCAGTAACCCGTCTTGAGACAGAAACAAACGCAAAACAGGCCAGAAGGATATATTTCAACATTT

TGAATTTGCACGTTTTTTGTAGGCCGGATAAGGCGTTTACGCCGCATCCGGCAATGGTGCTCAACGCCTG
ATGCGACGCTGGCGCGTCTTATCATGCCTACAACCCCCCTCATACCTTAAGGCGGATAAGGCATTTACTT
TATCACCGGGTCAAACCAACAAAGCGACCCGCCTTGATATAAATCCCAGGCTTCAGGTGCCCAACATACT
GTAACTGCGTTTCTGGCTTAAACGCAGAGACATCACCAATACGCATATGCATCAGATCGGCAGGGCTAAT
CCAGCCCGATTGCGCCAGTGTAAGTGGATGCCCGGCTTTGGCAAAGGCATCCGTGACAAATTCCGAACAA
AACCACGACTTTTTGTCTCCTTCGCCCACACTGCTTAACTGCGCTTTCGCCAGGCCGCTGACGCACTGTT
GGCGAAAATCCTCGGAGAACGGATTCAGTGAGCACATCTGGCGAGTCACCATAAAGGGAATAAATTCGAC
AATGCCGCGATAGTTATAACCGCTATCTTTGATTTTATTGGCAAACGCGGTGATATCTGTGGCTTGTTGC
GGGGTAAGATCCGGGACTCGTAAGACGAAAAGCTTATCACTATGCTTCATCGCTTTTTTAAGGGAAACGA
TCTGGACGCCAGCGCCTGTCGCTTCTGCAACGTTATTATCACCCAGAAAGATTGCAACGTGACTCACAGA
GGAAGTGCTGAAGACGCGGATTCCAAATGAGGTTACCCCAAGGCTTGAGGAGAACAGCAAATCGCCGGGT
TTGAGATCAGGTGCCGTTATTTCTTTTATTGATTGTTCGGTGAAAGAGCTTTGATGCTGGAATTTGACAG
CCCATGTTTTTGCCTCGGCATCTACTGCTGTGGCTGATGGGTCTGGCTGGCTGATATCAACGGTACAAGC
CGAAAGTAAAAGAAAGCTGGGGAGAAACAGGCGGCAGTACGCCTTTGGTTTATCCATTTTATACAATCCA
TGTAAAAAAAGGGCCCTGAAATTCAGGACCCTTTCTGGCATCAGCCTTTAATCTGTTTCACCAGATATTC
GACGATGTCACCAGTCTTAATTAACTGTTTCTCGCCGTTGCGACGATATTTATATTCGATATCGTCGTTG
TCGAGGTTACGGTCGCCCAGCACAATAGTGTGCGGAATACCGATCAGTTCCATATCAGCAAACATCACGC
CCGGACGCTCTTTGCGGTCATCCAGCAGCACTTCGATACCTTGTGCACGCAGTTCGCTGTACAGTTTCTC
AGCAAGCTCTTGTACGCGGAAGGATTTGTGCATGTTCATCGGCAGAATCGCCACCTGGAACGGCGCGATA
GCGTCAGGCCATACGATGCCTCGTTCGTCGTAGTTCTGCTCAATCGCCGCAGCTACCACACGCGTTACCC
CGATACCGTAGCAACCCATCGTCAGGATTTGGTTACGGCCATCTTCACCCTGTACGGAGGCTTTCAGTGC
TTCGGAGTACTTGGTACCCAGCTGGAAGATGTGACCAACTTCGATACCACGTTTGATCAGCAGCCTACCC
TGGCCATCCGGGCTTGGATCGCCAGCCACCACGTTACGGATATCTGCAACTTCCGGGGTAGCGACATCGC
GATCCCAGTTGATGCCGAAGTAGTGTTTACCATCGATGTTAGCACCAGCAGCGAAATCACTCATCGCCGC
AACGGTACGGTCAATCACCACCGGAATCGGCATGTTTACCGGACCCAGTGAACCCGGACCGGCTTTAACC
ACGGCACGAATTTCTTCTTCGGTCGCGAAAGTCAGCGGGCTTGCAACCTGCGGCAGTTTTTCTGCTTTAA
CTTCGTTCAGCTCGTGATCACCGCGCACCAGCAGCGCAACCTGCGGGAAGCTGCTGCCTTCAACCGCTTT
AACCAGCAGAGTCTTAACCGTTTTCTCAATCGGCAGATTGAACTGTTCAACCAGTTCCGCGATGGTTTTC
GCGTTCGGCGTATCAACCAGCGTCATTTCCTGGGTAGCAGCAGCGCGCGGTTCTTTCGGCGCGATAGCTT
CTGCCAGTTCAATGTTCGCTGCATAGTCAGAGGTGTCGGAGAAGACCACATCGTCTTCACCGCTCTGCGC
CAGCACCTGGAATTCGTGAGAGGCGCTGCCGCCGATAGAACCGGTGTCGGCTTGTACGGCGCGGAAATCC
AGCCCCATGCGGCTGAAGATTTTGCTGTAGGCCGCATACATTGCATCGTAGGTTTCCTGCAGGGATTCCT
GAGAAGTATGGAAAGAGTAAGCATCTTTCATCAGGAATTCGCGGGAACGCATGACGCCGAAACGCGGACG
CACTTCGTCGCGGAACTTGGTCTGGATCTGATAGAAGTTCAGCGGCAGCTGTTTGTAAGAGCTAAGCTCG
TTACGAATCAGGTCAGTGATAACTTCTTCATGAGTTGGGCCGAGTACGAACGGACGCTCGCCACGGTCAA
CAAAACGCAGCAGTTCCGGACCGTACTGTTCCCAACGACCACTCTCTTGCCACAAATCGGCTGGCTGAAC
CACCGGCATCGACACCTCGATCGCACCGGCGTTGTTCATCTCTTCACGCACGATGTTTTCGACTTTTTTC
AGAACGCGCACGCCGGTCGGCAGCCAGGTATATAACCCGGAGGCCAGCTTGCGGATCATCCCGGCGCGCA
GCATCAGCTGATGGCTGATCACCTCGGCGTCGGCAGGTGTCTCCTTGAGAGTGGAGAGCAGGTATTGGCT
AGTACGCATGTTGTTACGGTTCCAGTTGGAAGGTAGAACAGGCTCAAGGCGAGCCTGGGACAAAAAAAAG
TGATTTAGTTTACCAGTGCAAAAGAAATGTCAAAAGAGAAGGGCGTGAATTTAACGCGGTTCCAGCGCAA
AGACTTCAAAACCTGCGTCGGTGACGCGCCAGCGAACGTTAAAATCATGTAGCCAGACGGCATAGGTTTT
GCCCGTTTCCTCACCTTTACGATAGGCCGGGCGCGGGTCCTGCGCCAGTACTTCGCGGATAAACAGCGTT
AACTGCGGATAACGCTTCTCCAGCGTCAAAAGCTGCTTTTCGACCTCTGCGGTAAAACTCACCGCCATCT
CTGCAGCTGGCGCACTTTGCGCATAGCTGGCACTGGCATCGGGAAGCGATTCGGCAAAGGGGAGATACGG
TTTGATATCCACTACCGGCGTACCATCGACCAGATCCAGACTGCCGAGCTTCAGAATCACGCTGTCTTTA
TGGCAAACAACCTCTTTCAGCTCTACCAGCGACATGCCAATTGGGTTAGGGCGGAAAGTAGAGCGTGTTG
CGAAAACCCCCATTCTGGCGTTACCGCCGAGGCGCGGTGGACGCACAGTCGGACGCCAGCCGCCTTCCAT
CGTTTGATGAAAGACGAAAAGGATCCATAAATGGCTGAACGCTTCCAGGCCGCGAACGGCGTCGGCCTGG
TTGTAGGGAGCAATGAGATGCAGTTCGCCGTTGGCGCTTTTTACCAGACCTGGCTGGCGCGGAACGGCGA
ACTTTTCTTTATAGGGCGAGCGAATAACGCCTATTTGCTCGAACTGGAAACTGCTCATTTCGCCGTAATG
TTAAGCGCAGAACCGATACATACAGCCTGACGATAGCAGCCTGGCGTACCGCTGGTGACTTCGCAGCTAT

GCAGTAATACAGCATTGGCTTTCATTTTAGAGGCGTTGATTTGCATCCGCTTACGTGCGGTTGGAATGCT
CGGCGGAGAGTCCTGATTAGAGGCCTGGCAAGAGTCGCCACTGACTTCACCGAGATCGCGGAACGGTTTG
CCGACTAATTCTTCTGCATTGGTATAAATTCGGACCGGCGTGGCGCGCGGCGCTTTCGGTTTTGCAGGCT
CCGCTTTCGGCTGGGGTGCAGTGCTTTGAACGGGTTCGACAGGGGATCTGCTTAACATGGAACAGCCGCT
TAGCATGAGTGCTACTAAACAGATCGGTAAAGCACGCATAGTATTTCCTCAATGTATGATCAAAACGTCA
ATATTGAATCAGGAGCTTGTAAAAATGACAAGACGGGCAAGCGCCCGTCCTGAATGATATTACAAATTGT
GGAAACAGCCTAAAAATTACCAGCCTTTAACAGCTCCGCCGTTAAACACTTTGTTTGCTGCTTCGTAAAC
TTCGTCAGACTGATAAGCCTGGACGAATTTCTTCACGTTCTCGGCGTCTTTGTTATCTTCACGCGTCACG
ATCAGGTTTACGTACGGGGACTCTTTATCTTCAACAAAGATACCGTCTTTCGCCGGAGTCAGGCCAATCT
GGCTGGCATAGGTGGTATTGATAACTGCCAGAGCGATTT**GCGCGTCGTC**

Fragment 6 (4kb)

CAGAGAACGCGGCAGTTGCGGTGCTTCCAGTTCAACAATTTTCAGATTTTTGGGGTTCTCAACAACATCA
AGAACGGTCGGCAGCAGGCCAACGCCATCTTTCAGTTTGATCAAGCCCACTTTTTGCAGCAGCAGCAGTG
AACGACCAAGGTTAGTTGGGTCGTTTGGCACGGCAACCTGCGAACCATCCTGCAGTTCATCCAGTGATTT
GATTTTCTTGGAGTAACCAGCAATCGGATAAACAAAAGTGTTGCCTACTGCGACCAGTTTGTAGCCACGA
TCTTTCAGTTGCTGATCAAGGTACGGTTTATGCTGGAAGGCGTTGGCGTCGATATCGCCTTTGCTCAATG
CTTCGTTTGGCAGAACATAGTCGTTGAAGGTTACCAGCTCAACGTCCAGGCCATATTTGTCTTTCGCAAC
TTTCTGCGCGACTTCTGCAACCTGCTGTTCGGCACCAACAATCACGCCGACTTTAATGTGGTTTGGATCT
TTTTCATCCTGACCGCAGCCTACCAGTGCCAGTGATCCGATCAGGGCTCCCACTGCCGCAAAGGTTTTGA
ATTTGAACGCCATACCTTATTCCTTCTTCAATTATTTATGTTGTGTTGAACGTTACTTGCGAGTGACAGC
CCGGACGATGCGGTCGCCTGCGAACTGAATTAAATAAACCAGAATGACCAGCAATACCAGTACCGTATTC
ATCACCGTCGCGTTATAGCCGATGTAGCCATACTGATAGCCAATCTGACCTAAACCACCGGCACCGACTG
CACCACCCATCGCGGAATAACCGACCAGGGTAATCAGGGTGATAGTTGCCGCATTCACCAGACCCGGCAG
CGCTTCCGGTAACAGCACCTTACGGACGATCTGCATCGGCGTGGCACCCATTGCGCGGGAAGCTTCAATT
AACCCGGTTGGGATCTCCAGCAGAGCGTTCTCGACCATACGGGCAATAAACGGTGCTGCACCAACGGTTA
ACGGAACAATCGCTGCCTGCAAACCAATCGATGTACCGACAATAACGCGGGTAAACGGAATCATCCATAC
AAGCAAGATAATGAACGGGATGGAACGGAAAATGTTCACAATCGCAGAAACGGTACGATACAGCTTCGCG
TTAGCAATAATTTGCCCCGGACGCGTGACATAAAGCAGAACGCCAACCGGCAGACCAATCACAAAGCCAA
AAAAACCGGATACGAAGGTCATTGCCAGCGTTTCCCATACGCCACGAACCAGCAGCCACATCATCGGCTC
AGACATAACCCAGTACCTCTACTTTTACATGGTGTTCCTGCAGCCAGGCAATGGCGGCTTGCGTATCTTG
TTGTGTGCCGTGCATTTCAGTCAGCATGATGCCGAACTTAACGCCACCGGCGTAATCCATCTGCGCGCTA
ATAATGTTGTTGTTGACGTTGAAACGACGCGCGGTTTCAGAAAGCAGTGGGGCATCGACCGATTGACCGG
TAAACTCCAGACGCAGCATCGGCACGCAGTCAGTAAATGGCTCCGCTTGCAGACGTTCCTGGTAATCTTC
CGGGATATCCAGATGCAGGGTCGACTGAATAAACTTCTGCGCCAGCGGCGTTTTCGGATGCGAGAACACT
TCACTTACCGTGTCCTGCTCGATCAGTTCTCCATTGCTGATGACCGCCACGCAATCACAAATGCGCTTCA
CAACGTCCATTTCGTGGGTGATCAACAGAATCGTCAACCCCAGACGGCGGTTGATGTCTTTCAGCAGTTC
GAGAATAGAACGTGTCGTTGCCGGGTCCAGCGCGCTGGTGGCTTCATCACACAGCAATACTTTGGGATTG
CTGGCTAACGCACGGGCAATTGCCACACGTTGTTTCTGCCCACCGGAAAGATTCGACGGGTAGCTATCAT
GCTTATCGCCAAGACCAACCAATGACAGCAATTCCGTCACGCGACGTTTGACCTCGTCTTTCGGTGTGTT
GTCCAGCTCCAGCGGCAGAGCCACGTTGCCAAAAACAGTACGCGAAGAGAGCAGGTTAAAATGCTGGAAA
ATCATACCAATCTGGCGGCGAGCTTTGGTCAACTCGGATTCTGACAGCGTGGTCAGTTCCTGGCCATCGA
CCAGCACGCTACCCTCGGTTGGGCGCTCCAGCAGGTTTACACAACGTATAAGCGTACTCTTACCCGCGCC
TGAGGCACCGATAACGCCATAAATTTGTCCAGCTGGCACATGCAGGCTGACGTTGTTCAACGCCTGGATG
GTGCGGGTGCCCTGGTGGAACACTTTGGTGATATTCGAAAGTTTTATCATTGATTATTTATTATCGTCAT
TAAGTTAGTCGTGGCATCTCGAATGCCTGAAACGGGCAACGCCGTCAATGAAATGGATGTTAAGGCATCC
AGACGTCTAAATCAATCAGGTTTATGCGAAGAGCACTTTCTTGCAGGTCGAAACATGCGATACTAGCGTC
ACATGCCTTATTAAGGAGCTATAAAAGGTGGCGAAGAGCGTACCCGCAATTTTTCTTGACCGTGATGGCA
CCATTAATGTCGATCACGGCTATGTCCATGAGATCGACAACTTTGAATTTATCGACGGTGTTATTGACGC
CATGCGCGAGCTAAAAAAAATGGGCTTTGCGCTGGTGGTAGTAACCAACCAGTCTGGCATTGCTCGCGGT

AAATTTACCGAAGCACAGTTTGAAACGCTGACCGAGTGGATGGACTGGTCGCTGGCGGACCGAGATGTCG
ATCTGGATGGTATCTATTATTGCCCGCATCATCCGCAGGGTAGTGTTGAAGAGTTTCGCCAGGTCTGCGA
TTGCCGCAAACCACATCCGGGGATGCTTTTGTCAGCACGCGATTATTTGCATATTGATATGGCCGCTTCT
TATATGGTGGGCGATAAATTAGAAGATATGCAGGCAGCGGTTGCGGCGAACGTGGGAACAAAAGTGCTGG
TGCGTACGGGTAAACCTATTACGCCTGAAGCAGAAAACGCGGCGGATTGGGTGTTAAATAGCCTGGCAGA
CCTGCCGCAAGCGATAAAAAAGCAGCAAAAACCGGCACAATGATTAAAAGATGAGCGGTTGAAATAAAAA
TGCATTTTTCCGCTTGTCTTCCTGAGCCGACTCCCTATAATGCGCCTCCATCGACACGGCGGATGTGAAT
CACTTCACACAAACAGCCGGTTCGGTTGAAGAGAAAAATCCTGAAATTCAGGGTTGACTCTGAAAGAGGA
AAGCGTAATATACGCCACCTCGCGACAGTGAGCTGAAAGCCGCGTCGCAACTGCTCTTTAACAATTTATC
AGACAATCTGTGTGGGCACTCGAAGATACGGATTCTTAACGTCGCAAGACGAAAAATGAATACCAAGTCT
CAAGAGTGAACACGTAATTCATTACAAAGTTTAATTCTTTGAGCATCAAACTTTTAAATTGAAGAGTTTG
ATCATGGCTCAGATTGAACGCTGGCGGCAGGCCTAACACATGCAAGTCGAACGGTAACAGGAAGAAGCTT
GCTTCTTTGCTGACGAGTGGCGGACGGGTGAGTAATGTCTGGGAAACTGCCTGATGGAGGGGGATAACTA
CTGGAAACGGTAGCTAATACCGCATAACGTCGCAAGACCAAAGAGGGGGACCTTCGGGCCTCTTGCCATC
GGATGTGCCCAGATGGGATTAGCTAGTAGGTGGGGTAACGGCTCACCTAGGCGACGATCCCTAGCTGGTC
TGAGAGGATGACCAGCCACACTGGAACTGAGACACGGTCCAGACTCCTACGGGAGGCAGCAGTGGGGAAT
ATTGCACAATGGGCGCAAGCCTGATGCAGCCATGCCGCGTGTATGAAGAAGGCCTTCGGGTTGTAAAGTA
CTTTCAGCGGGGAGGAAGGGAGTAAAGTTAATACCTTTGCTCATTGACGTTACCCGCAGAAGAAGCACCG
GCTAACTCCGTGCCAGCAGCCGCGGTAATACGGAGGGTGCAAGCGTTAATCGGAATTACTGGGCGTAAAG
CGCACGCAGGCGGTTTGTTAA**GTCAGATGTG**

Fragment 7 (6kb)

AAATCCCCGGGCTCAACCTGGGAACTGCATCTGATACTGGCAAGCTTGAGTCTCGTAGAGGGGGGTAGAA
TTCCAGGTGTAGCGGTGAAATGCGTAGAGATCTGGAGGAATACCGGTGGCGAAGGCGGCCCCCTGGACGA
AGACTGACGCTCAGGTGCGAAAGCGTGGGGAGCAAACAGGATTAGATACCCTGGTAGTCCACGCCGTAAA
CGATGTCGACTTGGAGGTTGTGCCCTTGAGGCGTGGCTTCCGGAGCTAACGCGTTAAGTCGACCGCCTGG
GGAGTACGGCCGCAAGGTTAAAACTCAAATGAATTGACGGGGGCCCGCACAAGCGGTGGAGCATGTGGTT
TAATTCGATGCAACGCGAAGAACCTTACCTGGTCTTGACATCCACAGAACTTTCCAGAGATGGATTGGTG
CCTTCGGGAACTGTGAGACAGGTGCTGCATGGCTGTCGTCAGCTCGTGTTGTGAAATGTTGGGTTAAGTC
CCGCAACGAGCGCAACCCTTATCTTTTGTTGCCAGCGGTCCGGCCGGGAACTCAAAGGAGACTGCCAGTG
ATAAACTGGAGGAAGGTGGGGATGACGTCAAGTCATCATGGCCCTTACGACCAGGGCTACACACGTGCTA
CAATGGCGCATACAAAGAGAAGCGACCTCGCGAGAGCAAGCGGACCTCATAAAGTGCGTCGTAGTCCGGA
TTGGAGTCTGCAACTCGACTCCATGAAGTCGGAATCGCTAGTAATCGTGGATCAGAATGCCACGGTGAAT
ACGTTCCCGGGCCTTGTACACACCGCCCGTCACACCATGGGAGTGGGTTGCAAAAGAAGTAGGTAGCTTA
ACCTTCGGGAGGGCGCTTACCACTTTGTGATTCATGACTGGGGTGAAGTCGTAACAAGGTAACCGTAGGG
GAACCTGCGGTTGGATCACCTCCTTACCTTAAAGAAGCGTACTTTGCAGTGCTCACACAGATTGTCTGAT
GAAAATGAGCAGTAAAACCTCTACAGGCTTGTAGCTCAGGTGGTTAGAGCGCACCCCTGATAAGGGTGAG
GTCGGTGGTTCAAGTCCACTCAGGCCTACCAAATTTGCACGGCAAATTTGAAGAGGTTTTAACTACATGT
TATGGGGCTATAGCTCAGCTGGGAGAGCGCCTGCTTTGCACGCAGGAGGTCTGCGGTTCGATCCCGCATA
GCTCCACCATCTCTGTAGTGGTTAAATAAAAAATACTTCAGAGTGTACCTGCAAAGGTTCACTGCGAAGT
TTTGCTCTTTAAAAATCTGGATCAAGCTGAAAATTGAAACACTGAACAATGAAAGTTGTTCGTGAGTCTC
TCAAATTTTCGCAACACGATGATGGATCGCAAGAAACATCTTCGGGTTGTGAGGTTAAGCGACTAAGCGT
ACACGGTGGATGCCCTGGCAGTCAGAGGCGATGAAGGACGTGCTAATCTGCGATAAGCGTCGGTAAGGTG
ATATGAACCGTTATAACCGGCGATTTCCGAATGGGGAAACCCAGTGTGTTTCGACACACTATCATTAACT
GAATCCATAGGTTAATGAGGCGAACCGGGGGAACTGAAACATCTAAGTACCCCGAGGAAAAGAAATCAAC
CGAGATTCCCCCAGTAGCGGCGAGCGAACGGGGAGGAGCCCAGAGCCTGAATCAGTGTGTGTGTTAGTGG
AAGCGTCTGGAAAGGCGCGCGATACAGGGTGACAGCCCCGTACACAAAAATGCACATGCTGTGAGCTCGA
TGAGTAGGGCGGGACACGTGGTATCCTGTCTGAATATGGGGGGACCATCCTCCAAGGCTAAATACTCCTG
ACTGACCGATAGTGAACCAGTACCGTGAGGGAAAGGCGAAAAGAACCCCGGCGAGGGGAGTGAAAAAGAA
CCTGAAACCGTGTACGTACAAGCAGTGGGAGCATGCTTAGGCGTGTGACTGCGTACCTTTTGTATAATGG

GTCAGCGACTTATATTCTGTAGCAAGGTTAACCGAATAGGGGAGCCGAAGGGAAACCGAGTCTTAACTGG
GCGTTAAGTTGCAGGGTATAGACCCGAAACCCGGTGATCTAGCCATGGGCAGGTTGAAGGTTGGGTAACA
CTAACTGGAGGACCGAACCGACTAATGTTGAAAAATTAGCGGATGACTTGTGGCTGGGGGTGAAAGGCCA
ATCAAACCGGGAGATAGCTGGTTCTCCCCGAAAGCTATTTAGGTAGCGCCTCGTGAACTCATCTCCGGGG
GTAGAGCACTGTTTCGGCAAGGGGGTCATCCCGACTTACCAACCCGATGCAAACTGCGAATACCGGAGAA
TGTTATCACGGGAGACACACGGCGGGTGCTAACGTCCGTCGTGAAGAGGGAAACAACCCAGACCGCCAGC
TAAGGTCCCAAAGTCATGGTTAAGTGGGAAACGATGTGGGAAGGCCCAGACAGCCAGGATGTTGGCTTAG
AAGCAGCCATCATTTAAAGAAAGCGTAATAGCTCACTGGTCGAGTCGGCCTGCGCGGAAGATGTAACGGG
GCTAAACCATGCACCGAAGCTGCGGCAGCGACGCTTATGCGTTGTTGGGTAGGGGAGCGTTCTGTAAGCC
TGTGAAGGTGTACTGTGAGGTATGCTGGAGGTATCAGAAGTGCGAATGCTGACATAAGTAACGATAAAGC
GGGTGAAAAGCCCGCTCGCCGGAAGACCAAGGGTTCCTGTCCAACGTTAATCGGGGCAGGGTGAGTCGAC
CCCTAAGGCGAGGCCGAAAGGCGTAGTCGATGGGAAACAGGTTAATATTCCTGTACTTGGTGTTACTGCG
AAGGGGGGACGGAGAAGGCTATGTTGGCCGGGCGACGGTTGTCCCGGTTTAAGCGTGTAGGCTGGTTTTC
CAGGCAAATCCGGAAAATCAAGGCTGAGGCGTGATGACGAGGCACTACGGTGCTGAAGCAACAAATGCCC
TGCTTCCAGGAAAAGCCTCTAAGCATCAGGTAACATCAAATCGTACCCCAAACCGACACAGGTGGTCAGG
TAGAGAATACCAAGGCGCTTGAGAGAACTCGGGTGAAGGAACTAGGCAAAATGGTGCCGTAACTTCGGGA
GAAGGCACGCTGATATGTAGGTGAAGCGACTTGCTCGTGGAGCTGAAATCAGTCGAAGATACCAGCTGGC
TGCAACTGTTTATTAAAAACACAGCACTGTGCAAACACGAAAGTGGACGTATACGGTGTGACGCCTGCCC
GGTGCCGGAAGGTTAATTGATGGGGTTAGCGCAAGCGAAGCTCTTGATCGAAGCCCCGGTAAACGGCGGC
CGTAACTATAACGGTCCTAAGGTAGCGAAATTCCTTGTCGGGTAAGTTCCGACCTGCACGAATGGCGTAA
TGATGGCCAGGCTGTCTCCACCCGAGACTCAGTGAAATTGAACTCGCTGTGAAGATGCAGTGTACCCGCG
GCAAGACGGAAAGACCCCGTGAACCTTTACTATAGCTTGACACTGAACATTGAGCCTTGATGTGTAGGAT
AGGTGGGAGGCTTTGAAGTGTGGACGCCAGTCTGCATGGAGCCGACCTTGAAATACCACCCTTTAATGTT
TGATGTTCTAACGTTGACCCGTAATCCGGGTTGCGGACAGTGTCTGGTGGGTAGTTTGACTGGGGCGGTC
TCCTCCTAAAGAGTAACGGAGGAGCACGAAGGTTGGCTAATCCTGGTCGGACATCAGGAGGTTAGTGCAA
TGGCATAAGCCAGCTTGACTGCGAGCGTGACGGCGCGAGCAGGTGCGAAAGCAGGTCATAGTGATCCGGT
GGTTCTGAATGGAAGGGCCATCGCTCAACGGATAAAAGGTACTCCGGGGATAACAGGCTGATACCGCCCA
AGAGTTCATATCGACGGCGGTGTTTGGCACCTCGATGTCGGCTCATCACATCCTGGGGCTGAAGTAGGTC
CCAAGGGTATGGCTGTTCGCCATTTAAAGTGGTACGCGAGCTGGGTTTAGAACGTCGTGAGACAGTTCGG
TCCCTATCTGCCGTGGGCGCTGGAGAACTGAGGGGGGCTGCTCCTAGTACGAGAGGACCGGAGTGGACGC
ATCACTGGTGTTCGGGTTGTCATGCCAATGGCACTGCCCGGTAGCTAAATGCGGAAGAGATAAGTGCTGA
AAGCATCTAAGCACGAAACTTGCCCCGAGATGAGTTCTCCCTGACTCCTTGAGAGTCCTGAAGGAACGTT
GAAGACGACGACGTTGATAGGCCGGGTGTGTAAGCGCAGCGATGCGTTGAGCTAACCGGTACTAATGAAC
CGTGAGGCTTAACCTTACAACGCCGAAGCTGTTTTGGCGGATTGAGAGAAGATTTTCAGCCTGATACAGA
TTAAATCAGAACGCAGAAGCGGTCTGATAAAACAGAATTTGCCTGGCGGCCGTAGCGCGGTGGTCCCACC
TGACCCCATGCCGAACTCAGAAGTGAAACGCCGTAGCGCCGATGGTAGTGTGGGGTCTCCCCATGCGAGA
GTAGGGAACTGCCAGGCATCAAATTAAGCAGTAAGCCGGTCATAAAACCGGTGGTTGTAAAAGAATTCGG
TGGAGCGGTAGTTCAGTCGGTTAGAATACCTGCCTGTCACGCAGGGGGTCGCGGGTTCGAGTCCCGTCCG
TTCCGCCACTTATTAAGAAGCCTCGAGTTAACGCTCGAGGTTTTTTTTCGTCTGTATATCTATTATTGCC
AGAATCGCAAAAATCCTCTGCATTTTACGCTCTTTTTCCTCAACAGTCTGAAGCCCATAATCACCTCAGT
TAACGAAAATAGCATTAAAAGAGGCATATTATGGCTATCCCTGCATTTGGTTTAGGTACTTTCCGTCTGA
AAGACGACGTTGTTATTTCATCTGTGATAACGGCGCTTGAACTTGGTTATCGCGCAATTGATACCGCACA
AATCTATGATAACGAAGCCGCAGTAGGTCAGGCGATTGCAGAAAGTGGCGTGCCACGTCATGAACTCTAC
ATCACCACTAAAATCTGGATTGAAAATCTCAGCAAAGACAAATTGATCCCAAGTCTGAAAGAGAGCCTGC
AAAAATTGCGTACCGATTATGTTGATCTGACGCTAATCCACTGGCCGTCACCAAACGATGAAGTCTCTGT
TGAAGAGTTTATGCAGGCGCTGCTGGAAGCCAAAAAACAAGGGCTGACGCGTGAGATCGGTATTTCCAAC
TTCACGATCCCGTTGATGGAAAAAGCGATTGCTGCTGTTGGTGCTGAAAACATCGCTACTAACCAGATTG
AACTCTCTCCTTATCTGCAAAACCGTAAAGTGGTTGCCTGGGCTAAACAGCACGGCATCCATATTACTTC
CTATATGACGCTGGCGTATGGTAAGGCCCTGAAAGATGAGGTTATTGCTCGTATCGCAGCTAAACACAAT
GCGACTCCGGCACAAGTGATTCTGGCGTGGGCTATGGGGGAAGGTTACTCAGTAATTCCTTCTTCTACTA
AACGTAAAAACCTGGAAAGTAATCTTAAGGCACAAAATTTACAGCTTGATGCCGAAGATAAAAAAGCGAT
CGCCGCACTGGATTGCAACGACCGCCTGGTTAGCCCGGAAGGTCTGGCTCCTGAATGGGATTAAGCCTCT

CTGACAGCTCCTCCGGGAGCTGTTTTTACATGCTCGCTAAGGAAATCGATAAAAGCCCGGATGCGCGTAC
TTACCGCACGGTCGCTGTAATAGACGGCACTGAATGGCATTTCCACTGGCAACACTTTATCTGCCATTAA
CTCCACCAATTCTCCGCGAGCGATTTCTTTGTCGATCATGTAGTCGGACAAACACGCAATCCCGTTGCCA
CTCAGGCAAAGCTGTTTCAGTGTTTCCCCACTATTGGATGACAAACCGTACTTCACCTCATGTAATTGTC
CATCGCTACGGGCTATCGGCCAGGTATTGAGGGAAGCGGGTTCAGTGAATCCCAGGCAAATATGTTGCTT
TAAATCGTCGATCGTTTCTGGCTTCCCGTAGCGGGAAATATAATCGGGGGAGGCGATAATTTTTCGATAA
CTGTTAAATAAC**GGCCTGGC**

Fragment 8 (5kb)

ACGTAAGCTGGAATCCGTTAACGTACCAGCGCGTATCGCGACATCCACTTTTCTTTCGATCAAATTAATA
ATCGTTTCGGAGGAGACTAGCGACAAAGTGACTTCCGGATAGCGTTCACGGAAAGGCTTAATTAACGGCA
TCAGAAAGTGCAGCACCACTGGAGTTGCGGCATCGATCCGTAACAGTCCACGCGGTGTATTACGCGTCTC
CATAATTTCTGATTCTGCCGCTGCCATCTCCTGCAAAATTGACTGTACGCGACGAAAATAACGCTCGCCT
TCTTCCGTCAGGCTAAGTTGTCGCGTGGTCCGATTAAGCAGGCTAACGCCAAGTTTCATCTCCAGCTTTT
TCACCGCCCGGCTTACCGCTGAGTTTGCTTGCCCTAATTGTTCCGCTGCCCGGCTAAAGCTGCCGCTTTC
GACGACCGAAACAAAAATGGCGAGTTCTTCCGACGTGGCTTTCATTTTTGCTCCTGTTGCAAAATAGAAG
AGATATTTTGAATTTATTTGTCATTAAACCATCAGGATGCGTGATATGTCATGCGATTTAATGTTCTCCA
TAATGAGCAAAATTCTGACCGGTGTAAGCACTTGCTTACATAACAATATACAATTGCTCGTTGAAAGAGT
GAGCTAAAATCCCTATAACAGTAGAACCCTCCCGAGTGCGGAAGGGTTGACGTAATAGAGGTTTCAAAGT
CAAAAGTGCGAAAAAACACCTATGCCATGCGCTATGTTGCCGGACAACCTGCGGAAAGGATCTTACCGCC
GGGGTCTTTTGCGAGCATCGGCCAGGCATTACCACCTGGGGAACCGTTAAGTACCGAAGAGCGTATTCGG
ATCCTGGTGTGGAACATATACAAACAGCAACGCGCTGAATGGTTGTCGGTATTAAAGAACTACGGCAAAG
ATGCACATCTGGTGTTATTGCAGGAAGCGCAGACAACGCCAGAGTTAGTACAGTTTGCGACCGCTAACTA
TCTTGCCGCCGATCAGGTACCCGCTTTCGTGCTGCCACAACATCCTTCTGGCGTAATGACCCTTTCGGCG
GCACATCCAGTGTATTGCTGCCCGTTACGTGAACGAGAACCCATTTTGCGTCTGGCGAAGTCGGCACTGG
TGACGGTCTATCCATTACCTGACACCCGCCTGTTGATGGTGGTTAATATACACGCCGTCAACTTCAGTCT
GGGCGTGGATGTCTATAGTAAGCAGTTACTTCCTATTGGCGATCAGATAGCTCACCACAGCGGCCCGGTC
ATTATGGCGGGAGATTTCAATGCCTGGAGCCGTAGAAGGATGAACGCGTTATATCGCTTTGCGCGGGAAA
TGTCGCTGCGCCAGGTGCGTTTTACCGATGATCAGCGCCGTCGGGCGTTTGGTCGCCCGCTCGATTTTGT
TTTCTACCGTGGTCTGAACGTCAGTGAAGCTTCTGTACTGGTTACGCGCGCTTCCGATCACAATCCGCTA
CTCGTTGAATTCAGTCCCGGCAAGCCTGATAAATAAGGTATGTCAGGTCTGCCACAGGGCAGACCAACGT
TTGGCGCTGCGCAAAACGTGAGCGCGGTGGTGGCGTATGACTTATCTGCCCACATGCTGGATGTCGTGGC
ACAAGCTGCCGAAGCCCGGCAACTGAAAAATATCACCACCCGCCAGGGATATGCCGAAAGTCTGCCATTT
GCCGATAACGCATTTGATATTGTTATCAGCCGTTATTCTGCCCATCACTGGCATGATGTTGGTGCAGCAC
TGCGAGAAGTGAATAGGATATTGAAACCTGGCGGTAGGCTGATTGTGATGGACGTAATGTCTCCGGGTCA
CCCAGTGCGCGACATCTGGTTACAGACGGTAGAAGCATTACGCGATACCTCTCACGTACGAAACTACGCC
AGCGGTGAGTGGTTGACGTTAATCAATGAAGCCAATCTGATAGTTGATAATTTAATTACAGATAAGTTAC
CGCTGGAATTTTCTTCATGGGTCGCGAGAATGCGTACGCCAGAAGCGTTAGTAGACGCTATTCGCATTTA
CCAACAGAGCGCATCGACAGAGGTGAGAACGTATTTTGCCTTGCAGAATGATGGCTTTTTCACCAGTGAT
ATCATCATGGTAGATGCACATAAAGCGGCATAAATAAAAAAGGCACCGGGGGAATCGGTGCCTTTTTATT
ATCTGGTTTGTCAGGAATCTGGCATGTTGTTGTTTTTCACAAACAACGTCAGCTTATCGCCTGGTTGCAG
ATTCGCAGTGTCGCTGTTCCAGCGCATCACATCTTTGATGTTCACGCCGTGGCGTTTAGCAATGCTTGAA
AGCGAATCGCCTTTGCGCACACGATACGTAATGCTATCGCTGTTGTTTGCCAGTCGCTGTGCGCTACTGC
CTGCACCAATCGTCAAACTTTGGCCTGGCTTCAGCTTAGATCCGCGCAGTTTGTTCCACTGCTGCAAATC
TTTGGTGCTTACGCCGAGACGTGAAGCGATACTTGAAAGCGTGTCGCCAGAGCGTACGGTGTAAACACGG
CTGTTAAGCGGCGTATTGTCGGCAACCAGCGTCGACTGTACAGCAGCAATTTCGCCTGAAGCCAGAGATT
CACGCAGTTGATCTGCATGCTTCTTTGGCACCATCACGTACTGCGGGCCACTTGCGCCCAGCGTGGAGCC
TTTTACGCCAGCGTTGAATGTCTTCAGCTTGCTGACGGAAATCCCCGCCATATCTGCTACCTTCGCCATT
TCAACCGGGCTGCTCAGGTGCACACGCGCCAGAGCACGGCTTTCATCGGTCGTTGGCAGACGTACGCCAT
AACGCTTGCTGTTTTTGAGAATATCACTCAATGCCAGCATTTTAGGCACGTACTGCTTCGTTTCCTGCGG

CAACGGTAACGACCAGAAGTCCGTGGATTTCCCACGCGCTTTGTTCGTTTTAATTGCCTTCATGACCCGA
CCTTCGCCGCTGTTATAAGCCGCTACGGTCAGAAGCCAGTCGCCGTCAAACATTTTGTTCAGACGCTGCA
TCATATTCAGCGCGGCAGTTGTTGAAGCAACAACATCGCGACGCGCGTCATAATTGCGGGTCTGTTTCAA
ACCATAATTGCGCCCCGTGCTCGGAATGATCTGCCAGATGCCTGCGGCATTGGCGCCAGACGTTGCGTGA
GGATCAAAAGCGCTCTCCACTATGGGTAGTAGTACCAGTTCCATAGGCATGTTACGTTTTTTAACTTGCC
CTGCTATCCAGTACATATACGGCTCTGCCCGTAAAGTTACATCGTGGAGATAGCTCTTATTGCGTAAATA
TTTCTGTTTCTGTTCGCGAATCCGGTCATTTTCCGGAATTCCCATCTTTAGCTCGTCGCCAATGAAAGCC
CACAAGTCACCATCTGGCGCGATAGACGTCCCATCGTCCATCCATCGTGCCTGACTTGTAAACTTTGCTG
CTTCCCCTTGACCAGCTGCAGAAAGGCTCTGTGCGTGCTGTTGAACGTTGCCGGTACTCTGGCAACCCAC
GAGCAGGACAGAGGCGAGTAATATCGCTTTTGCCTTCATGTGTGTGTCAATAGTTGCTTAAAAGACGACC
GATCATAACGGCGAACGGAGCCGATGACAAGAAAGTTTTATCAGAACCTATCTTTCTTTGACCTTAACCA
TGCAAAACGCTCTTCAGGTTGTTGCAATAATGTTTCTTCATTAATTACATTAATTAAATCAATATCTTCC
GTTCTTAAAAAAACATTAATTTGCCGCTCATTTTTCAGAATTACGGGTAGTGTTATTTGATTTTTTGCCC
GTAACTCCTTAACTTTACGATAATAATCATTTATGGACAAATCGTGCGGAAGAATACTCAAAGCAAACTT
CATATTTGATAAGGTATATTCATGAGCACAACATACCAATGTATCGTCAGGTAACGCACTTAACTTTTTA
AGTGATTGATACATTTGTGATGCTGTCCCTTCAAACAACCGACCACACCCACCAGAAAACAGTGTGTCGC
CGCAAAATAGATAAGGTTTACTGAAGTAACAGATATGTCCTAAAGTGTGACCCGGCGTAGCAATTACACT
AAATTCATGCCCCAAAACGAAGGCAGTTTCGCCATCTTTGACTACCTGTGTTGTTCCCTTATCTTGTGTC
TCTTGTGGACCATACACCACAATTTGTGGAAACTTTTCCACCAGTTCTTTTACGCCGCCAACGTGATCGT
GGTGATGGTGGGTGAGAAATATGGCCTCCGGTTGCCAGTTATTGGCGGCAATGGCGTTTAATACTGGCTC
TGCGTCTCCGGGATCGACAATCAGGCAGCGACCTGCTTCATCATTCAAAACCCAGATGTAATTGTCATCA
AAGGCGGGAATACTGTTAAGATTCATAGATTACCTCTCAGTGTGAAACGGAAGGTTGTGATGAAACCGGC
AAGAGTCCCTCAAACTGTCGTGGCTCCTGATTGCTGGGGCGATTTGCCCTGGGGAAAGCTTTATCGCAAG
GCGCTGGAGCGCCAGCTCAACCCGTGGTTCACTAAAATGTATGGTTTTCATCTGCTTAAGATTGGCAATT
TAAGCGCAGAAATCAATTGCGAAGCGTGCGCGGTTTCTCATCAAGTGAATGTTTCTGCGCAAGGAATGCC
CGTCCAGGTACAGGCGGACCCACTTCATCTTCCTTTTGCCGATAAATCCGTTGATGTTTGTCTACTGGCA
CATACATTGCCGTGGTGCACCGATCCGCATCGTTTATTGCGTGAAGCCGATCGGGTATTGATTGATGATG
GCTGGCTGGTCATTAGTGGCTTCAATCCCATCAGTTTTATGGGATTACGCAAACTTGTGCCGGTATTGCG
CAAAACCTCGCCCTATAACAGCCGGATGTTTACTCTGATGCGGCAGCTGGACTGGCTCTCTTTGTTGAAT
TTTGAAGTGCTACACGCCAGCCGTTTCCACGTTCTCCCGTGGAACAAACACGGAGGAAAACTATTGAATG
CGCATATTCCTGCGCTTGGTTGCTTACAACTTATTGTTGCCCGGAAACGGACTATTCCTTTAACGCTAAA
TCCGATGAAACAGAGTAAAAACAAGCCACGAATTCGCCAGGCGGTT**GGAGCCACC**

Fragment 9 (5kb)

CGGCAATGTCGTAAACCACAGGCTTAAACTTCAACTTGGTAGCCTGTATCTTCCAGTGTGGGATTCATCG
CCGCGGCACGAGCCAGTTCATCACAGCGTTCGTTTTCCGGGTGTCCGGCATGGCCTTTAACCCATTCCCA
TTTGATTTGATGCTGCCCCAATGCAGCATCAAGACGTTGCCAGAGATCGACATTTTTTACTGGTTTTTTG
TCTGCGGTTTTCCAGCCACGTTTTTTCCAGTTATGGATCCACTGGGTGATACCCTGGCGGACATACTGGC
TGTCGGTACTCAAAATGACTTCGCAATGTTCTTTTAACGCCTCCAGCGCGACAATAGCGGCCATCAACTC
CATACGGTTGTTGGTGGTGCGGGTGTAGCCAGCGCTAAAGGTTTTCTCGCGTCCGCGATAGCGTAAAATA
GCGCCGTAACCCCCAGGTCCTGGATTGCCCAGACACGAACCATCGGTGAAAATTTCTACCTGTTTAAGCA
TCTCTGGTAGACTTCCTGTAATTGAATCGAACTGTAAAACGACAAGTCTGACATAAATGACCGCTATGAG
CACTGCAATTACACGCCAGATCGTTCTCGATACCGAAACCACCGGTATGAACCAGATTGGTGCGCACTAT
GAAGGCCACAAGATCATTGAGATTGGTGCCGTTGAAGTGGTGAACCGTCGCCTGACGGGCAATAACTTCC
ATGTTTATCTCAAACCCGATCGGCTGGTGGATCCGGAAGCCTTTGGCGTACATGGTATTGCCGATGAATT
TTTGCTCGATAAGCCCACGTTTGCCGAAGTAGCCGATGAGTTCATGGACTATATTCGCGGCGCGGAGTTG
GTGATCCATAACGCAGCGTTCGATATCGGCTTTATGGACTACGAGTTTTCGTTGCTTAAGCGCGATATTC
CGAAGACCAATACTTTCTGTAAGGTCACCGATAGCCTTGCGGTGGCGAGGAAAATGTTTCCCGGTAAGCG
CAACAGCCTCGATGCGTTATGTGCTCGCTACGAAATAGATAACAGTAAACGAACGCTGCACGGGGCATTA
CTCGATGCCCAGATCCTTGCGGAAGTTTATCTGGCGATGACCGGTGGTCAAACGTCGATGGCTTTTGCGA

TGGAAGGAGAGACACAACAGCAACAAGGTGAAGCAACAATTCAGCGCATTGTACGTCAGGCAAGTAAGTT
ACGCGTTGTTTTTGCGACAGATGAAGAGATTGCAGCTCATGAAGCCCGTCTCGATCTGGTGCAGAAGAAA
GGCGGAAGTTGCCTCTGGCGAGCATAAATACCTGTGAAAGGCGCTAAAAATAGCGACTTGGGCGATTTTT
GCAGCAAACGATTCAAAAGATGAGAAAAACCGTTGACGAAGGTCGAGGCAATCCGTAATATTCGCCTCGT
TCCCAACGGAACACAACGCGGAGCGGTAGTTCAGTCGGTTAGAATACCTGCCTGTCACGCAGGGGGTCGC
GGGTTCGAGTCCCGTCCGTTCCGCCACTATTCACTCATGAAAATGAGTTCAGAGAGCCGCAAGATTTTTA
ATTTTGCGGTTTTTTTGTATTTGAATTCCACCATTTCTCTGTTCAATGATTTTACTCTGGCGTAGGTGCG
TGATTCTCGCTTGTTGTCTCATTCATTAAAATTCAATAATGATATCGAACCATTCAGCTTAAATATATTT
CTAGAGAATAAATTTATATTGATTAAATGAATGTATATTTCAAATTGATTTTGTTTGTTATTATTTAAGT
GAGGTATATAATTAGAGTCCGTTATCAATGCTAAATATTCTAATCATTATGACAGGCGAGGGAGTGTCCA
ATTATGAATTCAAAAAAGCTTTGTTGCATATGTGTGTTATTCTCGCTGCTTGCAGGATGTGCCTCTGAAT
CTTCTATTGATGAAAAGAAGAAAAAAGCTCAAGTCACACAAAGTAATATTAATAAAAACACTCCCCAGCA
ACTGACAGACAAAGATTTATTCGGTAATGAAACCACTCTGGCCGTATCCGAAGAGGATATTCAAGCTGCG
CTTGATGGAGATGAGTTCCGCGTTCCCCTTAATTCTCCTGTAATACTTGTTCAATCCGGCAACCGCGCAC
CGGAAACCATTATGCAGGAGGAGATGCGAAAATATTATACTGTTTCCACATTCTCTGGTATCCCGGACAG
GCAAAAGCCTCTGACTTGTAACAAAAACAAAGATAAAAATGAGAACGAGGATGTTGCCAGTGCTGAGAAT
ATGAACTGGATGCAGGCACTGCGTTTTGTGGCTGCAAAAGGACATCAGAAAGCGATTATTGTTTACCAGG
ATATGTTGCAGACAGGAAAATATGACTCTGCGCTGAAATCAACAGTATGGTCCGACTATAAAAATGACAA
ACTCACTGACGCTATCTCCCTGCGCTACCTGGTACGTTTCACGCTGGTGGATGTGGCAACAGGTGAGTGG
GCTACCTGGTCGCCGGTGAATTATGAATATAAAGTGCTTCCACCATTGCCCGACAAGAATGAAGCCAGTA
CGACTGATATGACAGAGCAGCAAATCATGCAACTTAAGCAAAAGACCTATAAAGCGATGGTAAAAGATTT
GGTTAACCGCTATCAATAATAAATTATATCTGCCGCCAGGAATTTCTGGCGGCAATAATACAAAATTCTT
TGCATAAGAAATTTTTCTTGACAAAGACAGTGCAAGAAAGGACATGGAATCCGGAGTTAAAAACTGTTTG
TTTTAGAATATTTAATTATTTAACTGCTGAATCTTCCTTGCGAGAAATATATCCATTATTACATTTCATG
CCATTTTTAATATAGATTGCTCCTGGGAAAATTCCCGTATTATTACCGCAGGGGTGATAATTAGTATTGA
CATATCCTGTGACAAAAGGAGCTATTAAAGGTGCTATTACGATAGCTATTAGTAAAAATATAAGAGTTAG
CTGTATTGTTATGTCTGTGGCGAAATTGACTACCTTCGTTTTTTTGATTAAGAATGATTTTATTATCGTA
AGTAAAATTACATGAATATTTAAAAAGGAAAACGACATGAAACCGAAGCACAGAATCAACATTCTCCAAT
CATAAAATATTTCCGTGGAGCATTTTATTATTGAATATAGAGGTTTAACTCCGGTAAAAAACAAAGAAGC
ATTGAATGCAGGGAAAAATAATATGGCCATAAAAAACATCGAAAGAAACTCTTTTAATTTAACATGTAAA
CGCATGGTTAATCCTCATATCACGGGTGGAGTGTTAAGAACATACATAAATGGAGTCATGTTTTCCCTTT
TCCATTTATCAAGTTCCTGTTGCCGTTTTAGTCCATCTCTAATTGCATATTTTAATTTTTCTGATAAATG
GCATTGAGCATCGATTTCATTTAAAACAACTGTACACACTATCCCTGTCAGTAAAATTGCTCCTCCGACA
AGAATTACTGGAAAGCCAAAGCTAACACAGGCAAAAGAAGCCAGACTTCCAATAGCGGTTGCAACACCAC
CGGCTATAACTGCTTTGGCCACATCCATTGAAAAATCGCCAAGAAAATTAACCAGATCTCGCTCTGAACT
CATAATAAATTCTATGGTTCTGCTGATTTAACCGCAGAGAAGTTCGCCACAGCAATCCGAAACCACTGGC
ACGTGGAGAATAAGCTGCACAGGCGTCTGGACGTGGTAATGAATGAAGACGACTACAAAATAAGAAGAGG
AAACGCAGCAGAATTATTTTCAGGGATACGGCACATTGCTATTAATATTTTGACGAATGAGAAGGTATTC
AAGGCAGGGTTAAGACGTAAGATGCGAAAAGCAGCCATGGACAGAAACTACCTGGCGTCAGTCCTTGCGG
GGAGCGGGCTTTCGTAGTAATACCCCGACTCTCCCCGTCCTTAAACACAACCCCCACTCACCACAACCTA
AACTCATCCGCATCCTGCCATGCCGGAAACTTTTCTCTATATTCCCGCAATGCTGCCATCGACAGCTCCG
CATCAATGCGCGTTGCCTGATGCGCGTCGGCAGTAGCGATAATCTCTCCTTGCGGATTAATCACCCGGCT
GTCACCGCGATAATGGCAGCCGTTGCCATCGCTGCCGACGCGATTGCATCCCGCCACATACGCCTGATTC
TCAATCGCGCGGGCCGTCAGCAATGCCTGCCAGTGCAGAGAGCGCGGAGCAGGCCAGTTGGCGACGTACA
GGGCGAGGTCATAATCGTTGAGATTGCGCGACCACACAGGAAAACGTAAGTCGTAGCACACCAGCGGCAA
AATACGCCAGCCGCGCCATTCCACAATCACTCGCGCATTGCCCGCTTTATAATGTAGATGCTCATCTGCC
ATGCGGAACAGATGACGCTTATCATAAAAATGTACCGTGCCGCCCGGCTCAACCAGCAAAAAGCGGTTAA
CCGAACCAGACTCCGTTTGTAATGCAACACTGCCTGCAATCAGCGCATTGCACTGCTGCGCCTTAGCTGT
CATCCAGTTCACTACGTCATCTTGTGCTAGCGACGAAGCTGCCGCTTCCATGGCAAAGCCGCTGGTAAAC
ATCTCCGGTAGAACGATCACATCGCGCCCGGTAATACCTTCCAGTTGACGATCAAAATGACGCAGGTTGG
CAGGACCATCCATCCACACCAGTGGTTGCTGCAAAAGCGTAATCTTCAAACCAGGCACGGTGTACAACTC
CTTTATGCGAAGGGTTTTATAACTTTAACACCTTATCAGGCAGTTGCCTTAGCGCAGAATAAATTGATAA

CAAATGCTGATATTGGAAATATCTGATTTGCAAATTATCGTGTTATCGCCAGGCTTTAGGAGGTTAATAA
CATGGGCAGGATAAGCTCGGGAGGAATGATGTTTAAGGCAATAACGACAGTCGCCGCTCTGGTCATCGCC
ACCAGTGCAATGGCGCAGGATGATTTAACCATTAGCAGCCTTGCAAAGGGCGAAACCACCAAAGCTGCAT
TTAATCAGATGGTACAAGGGCATAAGCTGCCTGCCTGGGTGATGAAAGGCGGTACTTATACTCCCGCACA
AACCGTAACGTTGGGAGATGAGACGTATCA**GGTGATGAGC**

Fragment 10 (6kb)

GCGTGCAAACCGCATGACTGTGGCTCGCAACGTATCGCTGTGATGTGGTCCGAGAAATCTAATCAGATGA
CGGGGCTGTTCTCGACTATTGATGAGAAAACGTCGCAAGAGAAACTCACCTGGCTGAATGTGAACGATGC
GCTTTCGATTGATGGTAAAACGGTGTTGTTCGCGGCGTTGACCGGCAGCCTGGAAAACCATCCGGATGGC
TTTAATTTTAAATAATTAGCGGATAAAGAAACGGAGCCTTTCGGCTCCGTTATTCATTTACGCGGCTTCA
ACTTTCCGCACTTTCTCCGGCAACTTTACCGGCTTCGTCGCCAGCTCTTCCGGATCAAAGTCATCAACGT
TAATACTGCGCAGACGGCTTTCTTCAGCTTTCACCAGAATAGCGGCTTCATCTTTATCAATCAGCCCCTT
CACCAGCGCGTTGTGCGCCAGTTCATCCAGACGGGTAAACGGCAGGTTTTTACCCAGCTCTTTACAGATC
CGCTGATGAATTGGGTCGGCGGCAATCACATCCACCAGCGCCTCTTCCAGCAAGCCAACCGGATTATGCT
CGCTCGGCGTCAGGTACTGACCGCGACCAATGCGGGAACGGGTGGCGTTCGGCACTTGTAAAATCTTCGC
CACTTTATGATCCAGCTTGTCAGAAGGTGCCAGATAATGACGTCCGGTCGGGAAGATCACCACATTCAGC
AGCCCGGCAACCACGCGGTTCGGGAAGTTTTGCAGTAAATCATCCATCGCCTGTTCAGCCTGATACAGCG
CATCTTGTACGCCCCAGTGCACCAGCGGCAGGTCGGCTTCATTACGGCCTTCGTCGTCATAACGCTTCAG
CACGGCAGAGGCGAGGTAGAGCTGGCTTAAAATATCCCCCAGACGGGCCGAGATGCGCTCGCGACGTTTC
AGGCTGCCGCCCAGCACTGCCATCGAGACATCAGAAAGCAGGGCGAGGTTGGCGCTCAGGCGGTTCAGGT
GCTGATAGTAGCGTTTAGTGGCATCGCCGGTTGGCGTGCTGCTGGTTAAACCGCGCGTCAGGCCCAGCCA
GAAGCTGCGAACTTTGTTGCTACCGACGTGACCGATATGTTTGAACAACAGTTTATCGAACGCGTTGACG
TCATTGTTCTTCGCCGCTTCCATCTCTTCCAGCACGTACGGATGGCAACGAATCGCTCCTTGTCCGAAGA
TCATCATGCTGCGGGTCAGAATGTTAGCCCCTTCAACGGTGATGGCAATCGGTGCGCCCTGGTAAGCACG
CGCCAGGAAGTTGCTTTGCCCGAGCATAATGCCTTTACCGCCGGTAATATCCATCGCATCAATAATCGAC
TGCTGCCCGCGGTGGGTACAGTGATACTTAACGATAGCCGACAGCACGGCAGGTTTTTCGCCGAGCATAA
TGCCGTAGGTAATCAGCGATGCCGCAGCATCCATCACGTAGGCATTACCGGCAATACGCGCCAGCGGCTC
TTCAATCCCTTCCATCTTACCAATAGAGATTTTGAACTGACGGCGAATGTGAGCATACGCGCCGGTTGCC
AGCGCTACCGATTTCACGCCGCCGGTTGAGTTGGAAGGCAGGGTGATGCCGCGGCCTACCGAGAGGCACT
CCACCAGCATCCGCCAGCCTTGCCCGGCCATTTTCGGCCCGCCGATGATGTAATCGATCGGCACGAAGAC
ATCTTTACCGCGCGTCGGTCCGTTCTGGAACGGTACGTTCAGCGGGAAGTGGCGACGACCAATTTCCACG
CCCGGCGTGGTGGTTGGGATCAGCGCACAGGTAATGCCTAAATCTTCTGCACCGCCGAGTAATTTTTCCG
GGTCGGAGAGTTTAAACGCCAGCCCAAGCACGGTCGCAATCGGTGCCAGCGTAATGTAGCGTTTGTTCCA
GGTCAGACGCATCCCCAGCACCTGCTGGCCCTGCCATTCGCCCATGCAGACAATCCCGGTGTCCGGAATC
GCGCCCGCATCGGAACCCGCTTCCGGGCTGGTCAGTGCAAAGCAGGGGATCTCCTGACCACGCGCCAGAC
GCGGCAGATAGTGATCTTTCTGCTCGTCAGTGCCGTAATGTTGCAACAGTTCGCCCGGGCCTAATGAGTT
TGGCACGCCGACGGTAATCGCCAGGATCCCGCTCACGCCGGAGAGTTTTTGCAGCACGCGAGACTGGGCA
TAAGCCGAGAACTCCAGCCCGCCGTACTCTTTTTTGATGATCATCGCGAAGAAACGATGCTCTTTAAGGT
ACGCCCACAACTCCGGCGGCAGATCCGCCAGCTCATGGGTGATCTGGAAATCATTCGCCATCCGGCAGGC
TTCTTCTACCGGGCCGTCGAGAAACGCTTGCTCTTCGGCGGTCAGGCGCGGCTGCGGATAGTTATGCAGC
TTTTTCCAGTCCGGCTTGCCCTGGAACAAGTCGCCCTCCCACCAGGTGGTGCCCGCATCAATCGCTTCTT
TCTCAGTGCGCGACATCGGCGGCATCACCTTACGGAAACCGCGAAATACCGGCGCGGAAATCATCGACTT
ACGCATAGGCGCAAAGTTAAATGGCACGAGGATAATGGCCAGAGGCACCAGTACCCACGCCGACCACAGA
CCAGCAACGCCGAGGGCGGCTGTCCAGGCGAGCAAAATCAGACTGCTGATAAATAAGCTCACGCGGTGAT
AGAACAACGCGCCGAGCAGGACAACCGTAGCGAGAATACTCAAAATCATCATAACGAAAAGCCCCTTACT
TGTAGGAGGTCTGACCACTTGTGATGATATGGTTGTAGTGGATGTAAAAACATTTAGCAATATGTTTACA
ATATAATTACAACAAAGCTCACATTGTTGCTGTTTTTATCCGCACTTCAGGTCAAAAAGTCCTGGTCATA
GCACCTGCCCGTACTTCTCGCTTTTGGCGGTATCCGGTACACTGCATTTTGTCTATTACATTTATGCTGA
AGGATATCCTCATGTACCAGGATCTTATTCGTAACGAACTGAACGAAGCGGCGGAAACGCTGGCTAACTT

TTTAAAAGATGACGCCAATATTCACGCCATTCAGCGCGGCGGTCCTGTTAGCAGACAGCTTTAAAGCC
GGTGGCAAAGTGCTTTCCTGCGGCAACGGCGGTTCCCATTGCGACGCTATGCACTTTGCCGAAGAGTTGA
CCGGTCGCTACCGTGAAAACCGTCCGGGCTACCCGGCGATTGCTATTTCTGACGTTAGTCATATTTCCTG
CGTCGGTAATGATTTCGGTTTCAATGATATTTTCTCCCGCTACGTTGAAGCGGTAGGTCGCGAAGGCGAT
GTACTGCTGGGGATCTCCACCTCCGGTAACTCTGCAAACGTGATCAAAGCGATCGCAGCGGCGCGTGAGA
AGGGAATGAAAGTGATCACCCTGACCGGTAAAGACGGCGGCAAAATGGCTGGCACGGCGGATATCGAAAT
TCGCGTACCGCACTTTGGTTATGCCGACCGCATTCAGGAGATTCACATTAAAGTGATCCATATCCTGATC
CAGTTGATTGAAAAAGAGATGGTTAAGTAAGTCTGGCGTAGGCCGGATAAGACGTTTACGCCGCATCCGG
CATTTGTGCGCTGATGCCTGATGCGACGCTGACGCGTCTTATCATGCCTACAAATCTGTACGCGAACCGT
AGGCCGAATAATGCGTTCACGCCGCATCCGACCTGAAAATTCTTAAATCAATCTTCGCCGGGGGCCATGC
GCTCCCGCTGTTGTGGAGGTTACCCATGTGCGAATTGCTCGGGATGAGCGCCAACGTCCCTACCGATATC
TGCTTTAGTTTCACCGGGCTTGTACAGCGTGGTGGTGGAACCGGGCCACATAAAGATGGCTGGGGCATTA
CCTTTTACGAAGGTAAAGGCTGTCGCACATTTAAAGATCCACAACCCAGCTTTAATTCCCCCATCGCCAA
ACTTGTCCAGGACTACCCGATAAAATCCTGTTCGGTGGTGGCTCATATTCGCCAGGCTAATCGGGGCGAG
GTGGCGCTGGAAAATACTCACCCATTTACCCGCGAGTTATGGGGGCGTAACTGGACTTATGCCCATAACG
GACAACTGACGGGCTACAAATCACTGGAAACCGGCAACTTCCGCCCGGTAGGCGAAACCGACAGCGAAAA
AGCCTTCTGCTGGCTCCTGCATAAATTAACGCAGCGTTACCCGCGCACACCGGGCAACATGGCGGCGGTA
TTTAAATATATCGCCTCACTGGCGGATGAACTGCGGCAGAAGGGCGTTTTCAACATGCTGCTTTCGGACG
GGCGCTATGTAATGGCGTATTGCTCGACTAATTTACACTGGATCACCCGCCGCGCGCCGTTTGGCGTGGC
AACGTTGCTGGATCAGGATGTGGAAATCGACTTCAGCTCGCAGACCACACCGAATGATGTGGTCACGGTG
ATTGCAACACAGCCGCTGACGGGCAATGAAACCTGGCAAAAGATTATGCCAGGCGAATGGCGCTTATTTT
GCCTCGGGGAGCGTGTAGTTTGATGCCAGTTGTGGCTGCACAACTTCGTGGCTTAACGGCTTGCTGACCA
CGTAACGACCATTGACCACAGAAACGGTTGGTGGCTTACGGGTTTGCTCAAAGTAGTCGTAACCCGGCTT
CAGTTGCTCCCAGAAGTCCTTAAAGTTGGAATATTTATGGCGCTTCATATTGGCGTCGGTCATGCGGAAC
GGGTAAATACTCACTTGCACGCTCGGCTGACCAAACACCAGCGCACCAGTAACGAACTGGAATATCTCAT
CAATACCCTGATTGGTCATTGCGTAGCAGCCGATGGAAACACAATCGCCGTGGATCATCAGGTATTTCCC
TTCATAACCATGCGCACGGTCATAGGCATTGGGGAAACCAATATTAATCGCTTTGTAGTAACGGCTGTCT
GGTTTTAACTGATTACGCTGGACGCTATAAAACCCTTCCGGGCTTTTGAAATCGCCCTGACGCTGTTTTG
GCCCTAAGCCGCCGGAATATTTACAGATTTTATAGCTGTCGAGCAGTTGATATTGCTCGCCCATTTTGAC
GTAGAGATCGAGCGTACGTTCTTCCTTGAAGATCTGGATGTAGACAGGGGATCCCATCAACTGCTGCTTA
TACTCTTTGCTCACAGGCGTGGTAGAGCTACTGCTGCCCAGCAAACCGGCAAATGAAACGCACGGGATCA
ACAACATCGCAAGAATTAATGCGATTTTACGCATACTGCTTATTCCTTGATAAAACGGTTACACACGCCA
GGACGGCAAAATGGATCCCAAATCGGAATAGTCTGGATTTGGAAGGCTCACATTATCACCAAAAGAGTTT
TACGCAAGCCTGTCGGCGCAGGGTTTACAAATTTCATCGGAAGCGGGCTTTACCCAAAGAGCGCCGCGTG
AGTTCCAGTTCTCTCAAATCGTAAAAGTTTATCGGTAAGTTTGTAAATCAGGATCCAGTCCGGTTCGACA
TGAGCATCGCGATAACCTTTCCATGAACCTTGCAGCGGGTGGTCTTTATAAACAGCTGGAAGCGGTAAAG
TATTATTGATAAGAAGCGTCATAAGATATTTCAATTTATTCATATCCTTATGACGCTTTTGTGCAAGTTT
TACATCCTTTGAATATTGTCCCGAGTATTCAATATCCCTTTGAATCATATTTAAATTCCTAATTTATCAA
ATAAATCATCGGCGTCTTTGGCCTTATGAACATCAATGCCAGCTTCGCTGTTTTTGATTGATTGAATGGT
TAATTGATTAGGCTCGCGTAAATCAAACGGCAATGCCTTTTCACGCGCGACCTTTGTGAGGGTTATGCGA
ACCAGGTCAGAGATGGTCAGCCCCATCCCGGCCAGTACGTCCGCTGCCTGATTCTTCAGATCTTCATCGA
TTCGGGCGCGAACAAACGCGTTAGCAGCCATTGGGGTATCTCCTGTGCTGTATATTTATTCAGCTTGAAT
TGTAGCTCAAATGAGCAACAGCGACAATCCATGAGTAATTTAATAAACGGATGTTTTTACATAATTAACT
GAAATATATATGTTAATTTTATAATAATGGTTTATTAAGTTTGTTCAGAAACGATCCGGGATACACTGCA
ACATTAAAGCATGACCAGCCATTATTGAGTATGTCCTTGCCGTCGATTCCATCCTTTGTATTGTCGGGAT
TACTGTTGATTTGTTTGCCGTTTTCTTCATTT**GCCAGCGCC**
